# Grain-Boundary-Rich Interphases for Rechargeable Batteries

**DOI:** 10.1021/jacs.4c10650

**Published:** 2024-11-08

**Authors:** Qidi Wang, Chenglong Zhao, Xia Hu, Jianlin Wang, Swapna Ganapathy, Stephen Eustace, Xuedong Bai, Baohua Li, Hong Li, Doron Aurbach, Marnix Wagemaker

**Affiliations:** † Department of Radiation Science and Technology, Delft University of Technology, Delft 2629 JB, The Netherlands; ‡ Shenzhen Key Laboratory on Power Battery Safety and Shenzhen Geim Graphene Center, Shenzhen International Graduate School, Tsinghua University, Guangdong 518055, China; § State Key Laboratory for Surface Physics, Institute of Physics, Chinese Academy of Sciences, Beijing 100190, China; ∥ Department of Biotechnology, Delft University of Technology, Delft 2629 HZ, The Netherlands; ⊥ Key Laboratory for Renewable Energy, Institute of Physics, Chinese Academy of Sciences, Beijing 100190, China; # Chemistry Department, BINA-BIU Center for Nanotechnology & Advanced Materials, Bar-Ilan University, Ramat Gan 5290002, Israel

## Abstract

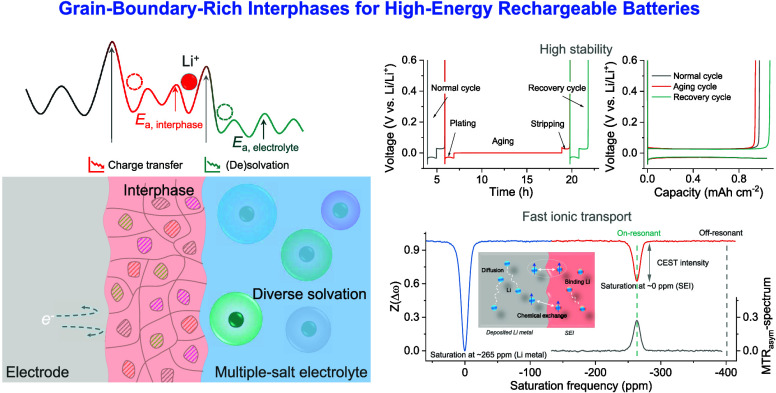

The formation of stable interphases on the electrodes is crucial for rechargeable lithium (Li) batteries. However, next-generation high-energy batteries face challenges in controlling interphase formation due to the high reactivity and structural changes of electrodes, leading to reduced stability and slow ion transport, which accelerate battery degradation. Here, we report an approach to address these issues by introducing multicomponent grain-boundary-rich interphase that boosts the rapid transport of ions and enhances passivation toward prolonged lifespan. This is guided by fundamental principles of solid-state ionics and geological crystallization differentiation theory, achieved through improved solvation chemistry. Demonstrations showcase how the introduction of the interphase substantially impacts the Li-ion transport across the interphase and the electrode–electrolyte compatibility in cost-effective electrolyte solutions optimized with multiple Li salts. The resulting interphases feature microstructures rich in inorganic grain boundaries with a diverse array of nanosized grains, presenting enhanced Li-ion transport. Comprehensive analyses revealed that this realizes remarkable electrochemical stability over extended cycling periods by inhibiting electrode corrosion, thus holding promise for high-capacity thin-Li-metal, Si-based anodes, and even Li-free anodes when paired with high-capacity oxide cathodes. This work opens new avenues to customize protective interphases on high-capacity electrodes, promoting the development of batteries with the highest energy density using cost-effective electrolytes.

## Introduction

The pursuit of high-energy-density lithium-ion batteries stand as a critical imperative in contemporary energy research, driven by the increasing demand for efficient and sustainable energy storage solutions.^1,2^ This evolution necessitates batteries with enhanced energy density, calling for electrodes with higher specific capacities, such as transition-metal oxide cathodes^3,4^ combined with Li-metal/silicon-based anodes.^5,6^ However, the application of high-capacity electrodes, that naturally are based on highly reactive active materials, has introduced formidable challenges in controlling the formation of protective and stable electrode–electrolyte interphases, emphasizing issues of side reactions, slow interfacial ion transport, increased interfacial resistance, and electrode structural degradation, all of which hasten the deterioration of battery life.^7^ Consequently, achieving effective passivating interphases is paramount, which requires the ability to prevent continuous electrolyte decomposition, by avoiding electron transfer while facilitating Li-ion transport with uniform current distribution.^7−12^ This has been a long-standing challenge since the exploration on the Li||TiS_2_ battery,^13^ hindering the development of high-performance batteries and fundamental scientific understanding for several decades.

The surface films formed naturally on the electrodes behave like solid electrolyte interphases (SEIs), as was initially described by Peled et al.,^7^ which typically consist of mixed organics (e.g., Li alkoxides, Li-alkyl carbonates, and polymers) and inorganics (e.g., metal oxides, carbonates, and fluorides).^14−16^ Despite simulation models have been proposed to understand interphase properties and their ion-transport mechanisms, underscoring the significance of grain boundaries,^17−19^ the rational design and translation into practical applications has not been achieved.^20,21^ In solid-state batteries, hybrid solid electrolytes, integrating both organic and inorganic phases, represent an emerging family that holds potential advantages from both components.^22,23^ Incorporating nanostructured inorganic fillers into organic polymer electrolytes has demonstrated the capability to enhance ionic transport by introducing abundant inorganic–polymer grain boundaries. This not only boosts the ionic conductivity within the polymer electrolytes but also promotes compatibility between the electrode and electrolyte, ensuring mechanical stability.^24,25^ Therefore, a burgeoning field is emerging to explore the prospect of engineering well-balanced hybrid interphases between electrodes and electrolytes, which could show promise for advancements in batteries.

**Figure 1:**
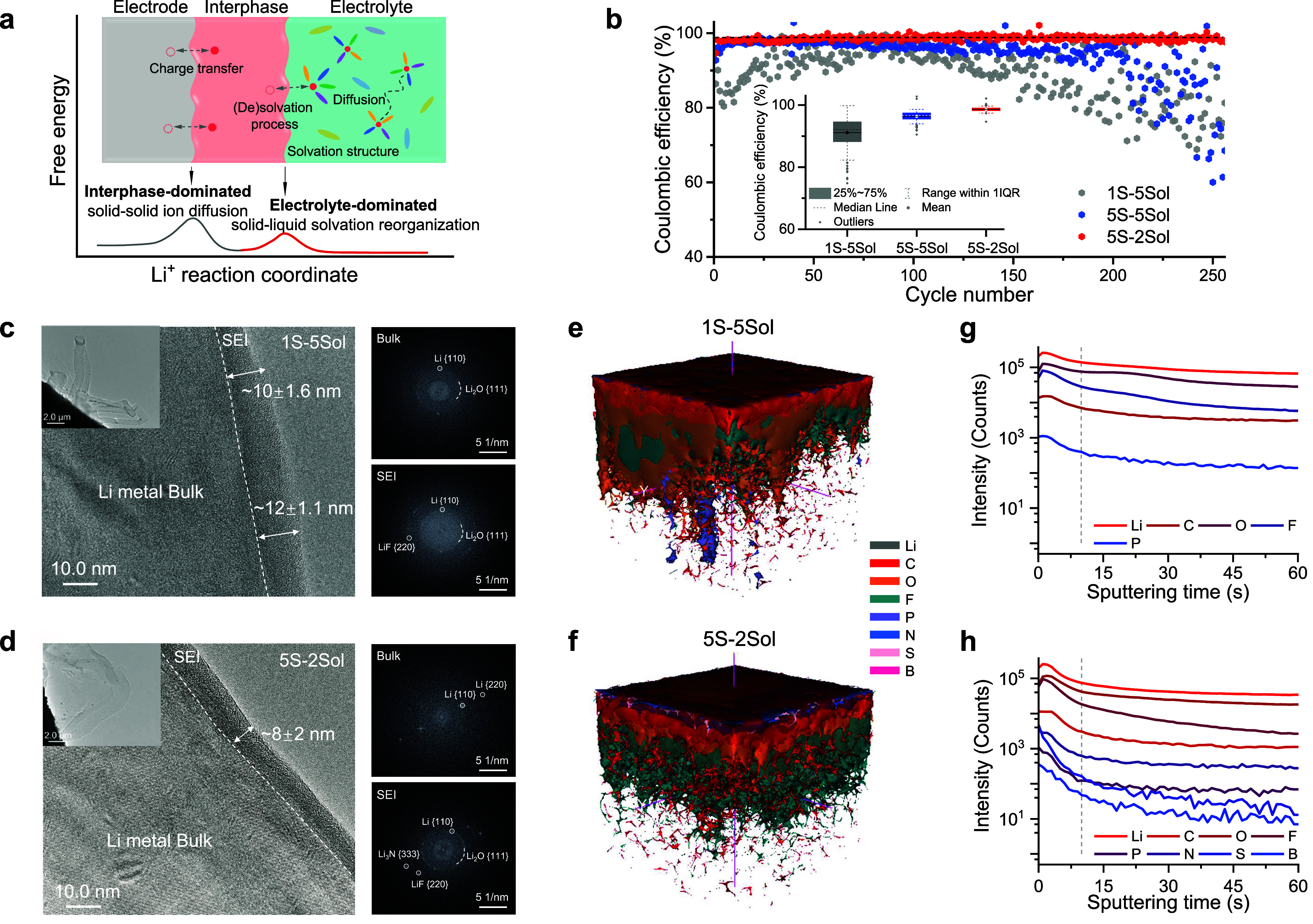
Electrode–electrolyte interphase and compatibility with Li-metal anodes. (a) Schematic diagram of Li-ion transfer between electrode and electrolyte. The process involves two mechanisms: interphase-dominated, based on solid–solid ion diffusion between electrode–electrolyte interphases, and electrolyte-dominated, arising from solid–liquid ion solvation reorganization. (b) CEs of Li||Cu cells. The statistical analysis of CEs in Li||Cu cells with box plots showing the median, 25 and 75% quantiles, whiskers indicating the range of ±1 × IQR (interquartile range), and outlying points plotted individually. (c,d) Cryo-TEM micrographs displaying the microstructure of deposited Li metal and SEI. Insets provide low magnification morphology. Corresponding selected-area electron-diffraction (SAED) patterns are collected in the SEI and Li metal bulk regions, respectively. The enlarged figures are shown in Figures S18 and S19. (e,f) Time-of-flight secondary ion mass spectrometry (TOF-SIMS) analysis of Li metal deposits after 20 cycles at 0.5 mA cm^–2^ for 2 h in different electrolytes. (g,h) Depth profiles of various chemical species over time for the 1S-5Sol and 5S-2Sol electrolytes.

In this work, we present an approach centered on engineering of electrode–electrolyte interphases to improve their ionic transport properties and enhance their chemical and mechanical stabilities for high-energy batteries. Through maximizing heterogeneous structure on the submicrometric level, uniform abundance of grain boundaries can be integrated within the interphases. Taking inspiration from the crystallization differentiation theory in geology,^26^ these interphases are successfully transformed onto the highly active electrodes using electrolytes made up of multiple salts, that lead to abundant boundaries between polymer and inorganic compounds. Multiscale characterizations reveal how the complex electrolyte-solution formulations affect the interphase structure and composition, influencing stability and interphase kinetics as well as charge transfer (Figure 1a). Through the introduction of a proof-of-concept multisalt electrolyte, it shows the capability to finely adjust solvation structures, leading to a substantial improvement of the ion/charge transfer processes within batteries, promoting the stable cycling of the high-energy batteries. These findings unveil a compelling pathway to tackle interfacial challenges for developing next-generation high-energy-density batteries using highly cost-effective electrolytes.

## Results and Discussion

### Electrolytes and Their Use in Li-Metal Anodes

The formation of interphases on electrodes is similar to the crystallization of igneous rock from the parent magma. The complex composition evolvement of the magma as minerals crystallize out in sequence determines the varying mineral compositions and textures in the resultant rocks, including the particle size and the arrangement of different mineral particles, which is known as crystallization differentiation.^26,27^ Inspired by this, a systematic study of multicomponent electrolytes is conducted to explore the possibility of transforming the interphases in high-energy batteries. In this context, commercially available salts of LiPF_6_, LiFSI, LiTFSI, LiNO_3,_ LiDFOB, and alkyl carbonate solvents, including EC, DEC, PC, DMC, and EMC, were used to prepare promising electrolytes, owing to their low cost and high accessibility. Importantly, this investigation can effectively demonstrate the influence of the interphase properties independent from the presence of the fancy electrolyte components. The prototype study involves a baseline electrolyte 1S-2Sol (1 M LiPF_6_ in EC/DEC, 1:1 by volume), a multisolvent electrolyte 1S-5Sol (1 M LiPF_6_ in EC/DEC/PC/DMC/EMC, equal volume ratios), a multisalt electrolyte 5S-2Sol (0.225 M LiFSI, 0.225 M LiPF_6_, 0.225 M LiDFOB, 0.225 M LiTFSI, and 0.1 M LiNO_3_ in EC/DEC, 1:1 by volume), and a multisalt/multisolvent electrolyte 5S-5Sol using the same five salts combined with the five solvents. The properties of these electrolytes were investigated and are described in the Supporting Information Note 1 and Figures S1–S10, where the results indicate that adding various salts allows tuning the reduction potentials of these multicomponent electrolytes, thus affecting the battery performance.

Coulombic efficiency (CE) and overpotentials are studied in Li||Cu cells (Figure 1b). Increasing the number of salt species appears more favorable than increasing the number of solvent species as the best performance is obtained for the 5S-2Sol multisalt electrolyte, which shows a higher plating/stripping reversibility with an average CE of ∼99.1% and a lower overpotential of ∼13 mV compared to ∼91.5% and ∼27 mV for the single-salt multisolvent 1S-5Sol electrolyte (Figure S11). This difference in cycling reversibility and overpotential was also observed in symmetric Li-metal cells, especially at higher current densities of 8.0 mA cm^–2^ (Figures S12–S14). Raising both the number of salt and solvent species in the 5S-5Sol electrolyte also increased the average CE, albeit to a lesser extent than that in the 5S-2Sol electrolyte, resulting in more capacity fading after 200 cycles (Figures 1b and S11). This may be a consequence of more solvent-rich coordination sheaths around the Li-ions (Figures S2–S5), which are expected to lower the interphase stability and Li-ion transport due to the formation of more oligomeric and organic species. The corresponding Li-metal plating/stripping morphologies within different electrolytes and Li-metal losses upon cycling, including the formation of residual dead Li and SEI, are systematically investigated using scanning electron microscopy (SEM) measurements and operando solid-state ^7^Li nuclear magnetic resonance (NMR) measurements (Supporting Information Note 2, Figures S15–S17). Results reveal that the Li-metal deposits in 5S-2Sol electrolyte are more compact and well-connected to the Cu substrate, leading to a lower fraction of dead Li-metal and Li species in the SEI. Since the Li-metal plating/stripping involves Li ions migration through the SEI that covers the Li-metal, the above results imply that SEI formation and its steady-state structure are decisive factors in determining the deposited Li-metal morphology and species that are formed by side reactions, which in turn depends on the electrolyte compositions.

### Microstructure and Composition of the Interphases

Electrochemical measurements show the advantage of using electrolytes containing multiple salts regarding the improved reversibility and kinetic behavior of Li anodes, while the impact of using a variety of solvents appears to be not significant. To reveal the impact of the electrolyte chemistry on the microstructure of the interphases, cryogenic transmission electron microscopy (cryo-TEM) was utilized.^28,29^ In the low-magnification cryo-TEM images (Figures 1c,d, and S18 and S19), whisker-like Li-metal deposits with a lateral diameter of around 0.6 to 1.5 μm were observed using the 1S-5Sol electrolyte. These deposits were covered with an uneven SEI layer, resulting in a porous structure and a relatively rough surface. In contrast, the Li deposits in the 5S-2Sol electrolyte exhibited isotropic morphologies with a larger average diameter and a smoother surface.

High-resolution images revealed that a thin and compact SEI layer of approximately 8 nm was formed on metallic Li deposits in the 5S-2Sol electrolyte (Figure 1d). In contrast, a relatively thicker and nonuniform SEI layer of around 10 to 14 nm was observed for Li deposits in the 1S-5Sol and 1S-2Sol electrolytes (Figures 1c and S20), consistent with the results from operando NMR and SEM that indicated the presence of more Li loss in the SEI. SAED measurements show that the SEI layer in the 5S-2Sol electrolyte was primarily composed of many small polycrystalline inorganic components. This indicates that the reduction of various anionic groups from the multisalts participates in the SEI formation, resulting in a homogeneous distribution of diverse nanosized inorganic crystalline grains embedded in an organic matrix (formed by the solvent-molecule reduction) (Figures 1d and S18 and S19), introducing more grain boundaries. In contrast, the SEI layers formed in the multisolvents 1S-5Sol electrolyte were dominated by organic components with a relatively small number of randomly dispersed crystalline inorganic domains. The amorphous matrices observed likely represent organic species from solvents decomposition, with much less crystalline compounds embedded in the SEI matrices formed on the electrodes in the 1S-5Sol electrolyte.^28^

The chemical composition of the interphases was analyzed using TOF-SIMS and X-ray photoelectron spectroscopy (XPS) to gain more insight into the elemental spatial distribution and bonding states. For the SEI formed in the 5S-2Sol electrolyte, O and F are dominantly distributed (Figures 1f,h and S21) while for the SEI formed in the 1S-5Sol electrolyte, C and O are the dominant elements, and more C is observed extending into the bulk of the electrodes (Figures 1e,g and S21). For the SEI formed in the 5S-2Sol electrolyte, the S, N, and B elements were also detected in the near-surface region, indicating the heterogeneous SEI structure. XPS measurements were consistent with the TOF-SIMS results (Figures S22–S25). The deconvoluted C 1s and O 1s profiles revealed larger fractions of C–O, C=O species in the 1S-5Sol electrolyte, indicating solvent-dominated interphase formation.^30,31^ In the 5S-2Sol electrolyte, C–SO_*x*_ and Poly(CO_3_^2–^) species are observed in addition to organic components, suggesting that the decomposition of FSI^–^, TFSI^–^, and DFOB^–^ anions participates in the SEI formation process^32^ (Figure S22). The diverse composition of the multisalt-derived SEI in the 5S-2Sol electrolyte is further supported by the presence of P–F, B–O, B–F, N–O, SO_*x*_ species, and more Li-containing inorganic components, including Li_2_O, Li_*x*_N, and LiF (Figures S23–S25). In the F 1s spectra, Li–F and P–F species are detected in both electrolytes; however, in the 5S-2Sol electrolyte, the C–F species may also come from other anionic groups^33^ indicated by an increased amount of Li–F species based on Li 1s spectra (Figure S23). The observed element distributions can be related to the intrinsic characteristics of the different electrolytes, where the multisalt 5S-2Sol electrolyte results in various anion-derived interphase components, contributing to the formation of a multicomponent grain-boundary-rich interphase. This interphase structure is held responsible for inducing fast Li-ion transport through the interphase, which is desirable for the operation of passivated electrodes.

### Li-Ions Transport across the Interphases

**Figure 2:**
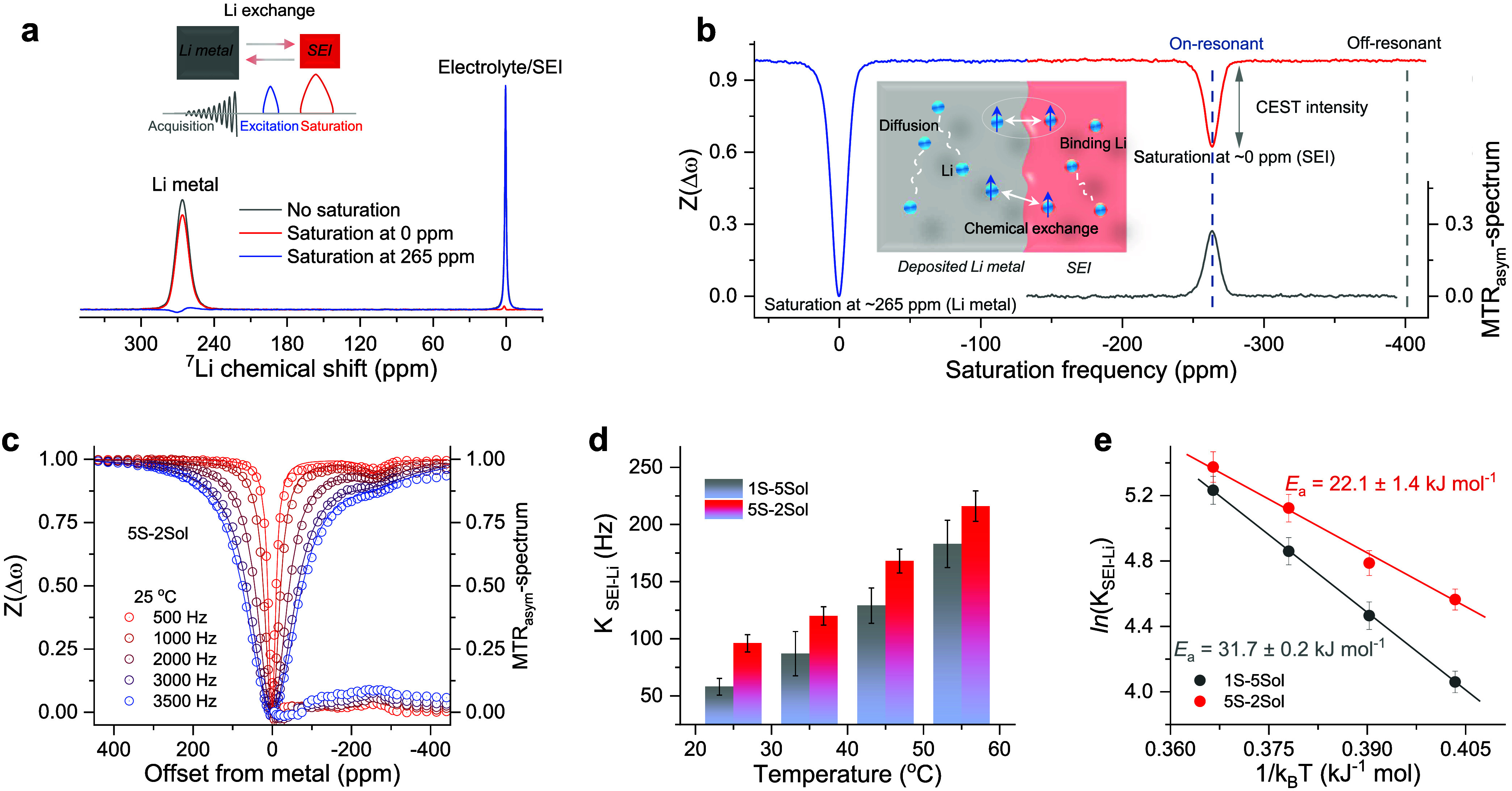
Interphase Li-ion kinetics. (a) Illustration of CEST approach. ^7^Li NMR spectra of Li-metal deposits immersed in an electrolyte acquired without saturation (gray) and acquired with a saturation pulse of 0.2 s and 3500 Hz at the SEI resonance (∼0 ppm, red) and the deposited Li resonance (∼265 ppm, blue). The inset demonstrates Li exchange during a CEST pulse sequence, where a saturation pulse is applied to the SEI site followed by an excitation on the resonance of Li metal. (b) Z-spectra obtained from Li-metal deposits as a function of saturation frequencies and MTR_asym_ quantitative analysis. Δω represents the applied frequency away from the Li-metal peak. The inset presents the schematic diagram of the CEST effect on the “deposited Li metal and SEI” two-pool system. Through applying a soft saturation pulse on the SEI resonance (∼0 ppm, pool I) at a certain cross-relaxation, Li exchange occurs between SEI and deposited Li (∼265 ppm, pool II.), resulting in the decrease of the deposited Li signal (CEST intensity). (c) Z-spectra obtained from Li-metal deposits with a saturation time of 0.2 s at 25 °C with various saturation powers. (d) Exchange rates of Li metal deposits via fitting the Z-spectra from the two-pool Bloch–McConnell (BMC) equation. (e) Li exchange rates and activation energy as a function of temperature. The activation barrier was obtained by fitting the Arrhenius equation.

Li-ions transport through the SEI is anticipated to depend on the composition/structure of the interphase, which was seldom studied because it is very challenging to directly probe the local ionic diffusivity. A powerful method to investigate the ion transport across the SEI in its native state (see Materials and Methods above) is NMR chemical exchange saturation transfer (CEST), commonly used in high-resolution ^1^H NMR and magnetic resonance imaging to quantify exchange rates between different chemical environments.^34−36^ The exchange between SEI and Li-metal deposits can be considered as the exchange between two pools of Li, where the Li metal presents a large pool and the SEI a small pool of exchangeable Li ions.^36^ A scheme of the CEST experiments is shown in Figure 2a. The CEST experiments apply a long saturation pulse to the small pool, during which continuous exchange will lead to the accumulation of transferred saturation, and then the resonance of the large pool will be detected.^37,38^ Applying a saturation pulse to the Li-metal frequency can lead to substantial signal reduction due to direct saturation. However, when the exchangeable pool is saturated via selective radio frequency (RF) irradiation at the SEI frequency, the saturation is transferred to the Li metal via chemical exchange, thus decreasing the signal of the metal peak. To quantitatively analyze the Li exchange between Li metal and the SEI, the normalized Li-metal signal intensity is monitored against the frequency of the off-resonance saturation: the so-called Z-spectrum^39^ (Figures 2b and S26). The magnetization transfer ratio asymmetry (MTR_asym_) signal derived from Z-spectra (see Materials and Methods) can be used to qualitatively compare the exchange rate in different SEI-Li-metal systems.^40^ For determining the exchange rate between SEI and Li metal in different electrolytes, Z-spectra with various RF saturation amplitudes were collected at temperatures from 25 to 55 °C for the Li-metal-SEI formed in the different electrolytes (Figures 2c and S27). In all cases, the CEST effect increases with the saturation amplitude, *B*_1_ and temperature.

A qualitative comparison suggests that the SEI formed in the 5S-2Sol electrolyte exhibits a larger CEST than the SEI formed in the 1S-5Sol electrolyte under the same conditions, indicating much better and effective Li-ion exchange between the SEI and the Li-metal when the surface films on Li were formed in the 5S-2Sol electrolyte system. To quantify these differences, we employed the two-pool BMC differential equation and fitted the Z-spectra acquired with multiple *B*_1_ simultaneously (see Materials and Methods and Table S1).^41−43^ The resulting interphase exchange rates (Figure 2d and Table S2) increase almost linearly with temperature in both cases, with the SEI formed in the 5S-2Sol electrolyte showing a higher exchange rate than the SEI formed in the 1S-5Sol electrolyte. This suggests that the diverse inorganic and grain-boundary-rich interphase formed in the 5S-2Sol electrolyte facilitates a faster Li-ion transport, contributing to a more uniform Li deposition (Figure S16) and to a better cycling performance. Furthermore, the activation energy for Li-ion transport across the surface films on the Li electrodes (i.e., Li-ion migration through the SEI) was determined using variable temperature measurements (Figure 2e). The direct determination of the energy barrier (via analysis of Arrhenius plots) showed that the SEI formed in the 5S-2Sol electrolyte system has a lower migration barrier for Li-ions exchange (around 22.1 kJ mol^–1^) than that of the SEI formed in the 1S-5Sol electrolyte solution (around 31.7 kJ mol^–1^), reflecting the higher Li-ions permeability through the SEI formed in the former electrolyte solution. The interphase resistance measured by EIS and analyzed by the distribution of relaxation times after cycling (Figures S28 and S29) showed that the overall impedance of cells using the 5S-2Sol electrolyte solution is lower than the impedance measured with cells containing the 1S-5Sol solution. For the impedance of the SEI corresponding to the time scale of 10^–4^ to 10^–2^ s,^44^ the cells with the 5S-2Sol electrolyte exhibit a value of approximately 13 Ω, which is significantly lower than the 27 Ω observed for cells with the 1S-5Sol electrolyte. These studies indicated much more effective Li-ions transport between the interphase and the electrode in the 5S-2Sol electrolyte, suggesting that the grain-boundary-rich interphase with diverse inorganic components formed in the multisalt electrolyte facilitates a faster Li-ion transport, contributing to improvements of the batteries.

### Stability of the Interphases

**Figure 3:**
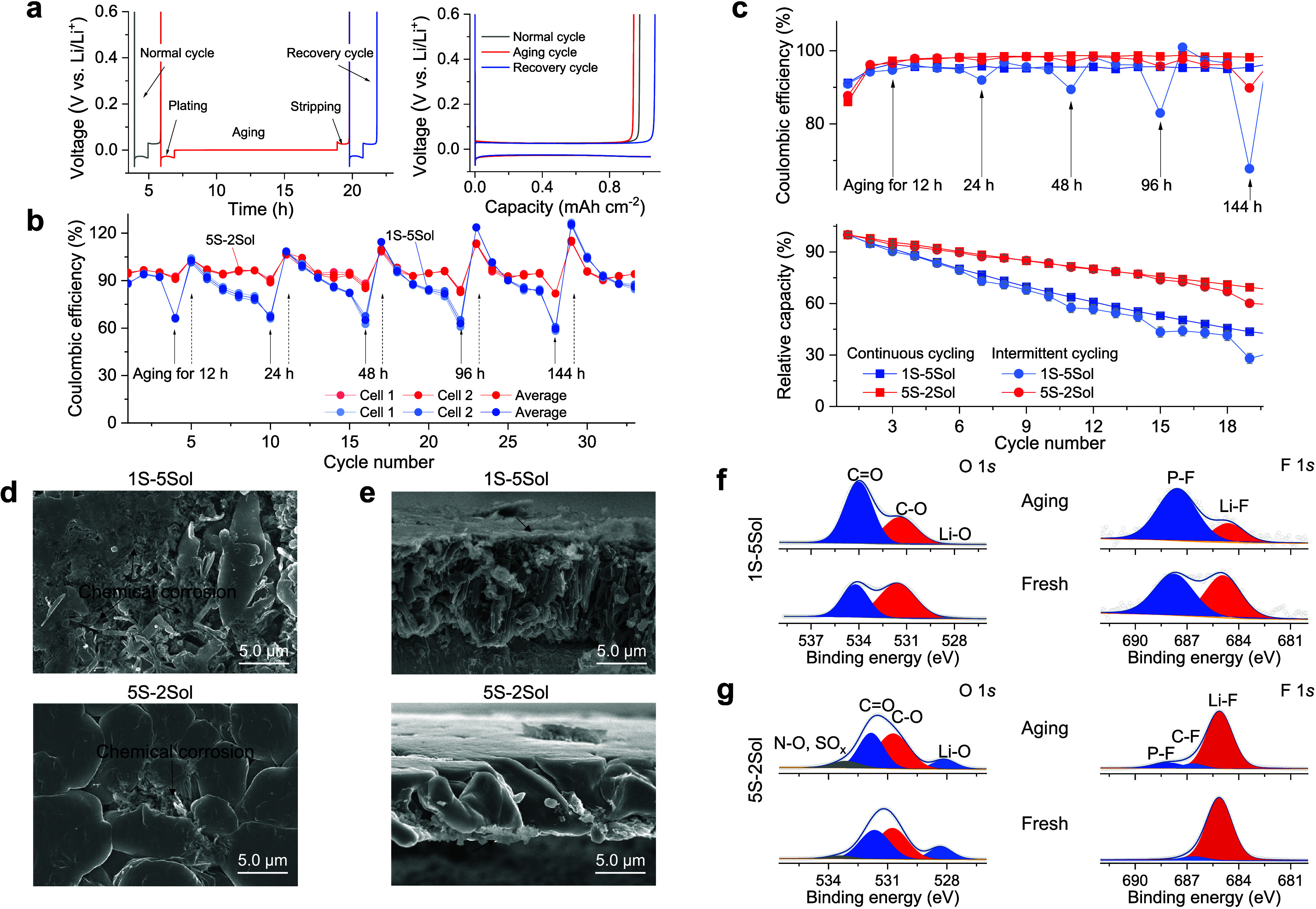
Interphase stability. (a) Illustration of the intermittent cycling protocol. Li||Cu cells undergo three cycles of continuous cycling at 1.0 mA cm^–2^ for 1 h to represent the normal cycle. Subsequently, Li metal is plated at 1.0 mA cm^–2^ for 1 h and aged for various periods before being stripped at 1.0 mA cm^–2^ to the cutoff voltage of 1.0 V, representing the aging cycle. Following the aging cycle, Li-metal is plated and stripped at the same current density, representing the recovery cycle. (b) Intermittent CE of the electrolytes for different aging times. (c) Relative discharge capacity retention and the corresponding CE of Cu||LiFePO_4_ cells cycled at C/5 with different electrolytes using continuous and intermittent electrochemical cycling protocols. Cu||LiFePO_4_ cells undergo two cycles of continuous charge–discharge at C/5, followed by an intermittent cycle with various aging periods and then three continuous cycles using the same current density. (d) SEM images of deposited Li after aging 120 h. Cells were cycled at 1.0 mA cm^–2^ for 1 h (1.0 mA h cm^–2^), underwent 20 cycles, and then Li was plated for 1 h. (e) Images of deposited Li metal on Cu foil from a cross-sectional view. (f,g) XPS data related to O 1s and F 1s spectra measured from electrodes after cycling in the electrolytes (as marked therein). Fresh samples represent electrodes measured after 20 cycles, while aged samples were taken from cells aged for 120 h.

Interphase stability is a crucial factor influencing electrode performance but is less directly studied. Here, an intermittent electrochemical test protocol, including a calendar aging step (Figure 3a), was introduced to assess the interphase stability. This method can account for chemical corrosion, thermodynamic driving forces toward interphase degradation, and the electrolyte/electrode consumption during storage beyond continuous electrochemical cycling.^45^ In addition, the cycling efficiency measured for the recovery cycles can reflect whether Li is lost as dead Li-metal or through side reactions that form surface species precipitating on the surface of the Li electrodes. Intermittent cycling measurements of Li||Cu cells with aging times ranging from 12 to 144 h were performed for different electrolytes (Figures 3b and S30–S32). During these intermittent cycling experiments, the cells containing the 5S-2Sol and the 5S-5Sol solutions displayed small CE fluctuations between the aging and recovery cycles, along with higher average CE values, while the cells containing 1S-5Sol electrolyte exhibited significant CE fluctuations and a lower average CE value (Figures 3b, S30 and S31). Analysis of the CE statistics for different aging times (Figure S32) suggests two capacity-loss mechanisms: (1) the dead Li-metal formation and (2) the SEI growth during aging. First, with all the three electrolytes studied herein, dead Li-metal form during each aging step, but it can be partially recovered in the next cycle for cells containing the multisalt electrolyte, as reflected by CE values larger than 100%. Second, a larger capacity loss during cycles was observed for cells containing the 1S-5Sol electrolyte, indicating irreversible loss of active Li to the SEI formation. By comparison, the Li-metal electrodes cycled in the multisalt (5S-2Sol and 5S-5Sol) electrolytes showed a smaller capacity loss, as more “lost” capacity (per cycle) could be recovered upon the charging processes in consecutive cycling (Figures 3b, S31 and S32). The cells using the 5S-2Sol electrolyte exhibited a higher capacity retention and higher average CE during continuous electrochemical cycling (Figure 3c). Similarly, intermittent electrochemical cycling of Cu||LiFePO_4_ cells (Figures 3c and S33) revealed smaller CE fluctuations and capacity loss on aging using 5S-2Sol electrolyte compared with 1S-5Sol electrolyte.

To investigate the underlying principle responsible for the observed electrochemical properties, the morphologies and surface chemistry of the deposited Li were examined after aging. Figure 3d illustrates that Li-metal deposits formed in the 1S-5Sol electrolyte underwent severe chemical corrosion during aging, resulting in the formation of extensive side-reaction products on the surface compared to the fresh samples (Figure S17). In contrast, the Li-metal deposits formed in the 5S-2Sol electrolyte maintained a cleaner and smoother surface. SEM energy-dispersive spectroscopy (EDS) mapping (Figures S34 and S35) indicated that the corrosion products mainly consist of C and O containing compounds, derived from the solvents. The SEM images of electrodes’ surfaces and cross sections (Figure 3d,e, respectively) demonstrate different corrosion behaviors of the Li deposited on Cu in the 1S-5Sol and 5S-2Sol electrolytes. For the Li deposits formed in the 1S-5Sol electrolyte, the decomposition products mainly form on the top of corroded Li-metal, while in the 5S-2Sol electrolyte, the corrosion is observed near the current collector. Thus, the observed corrosion processes in these two electrolytes can be attributed to different categories: top corrosion, associated with irreversibly formed Li-containing products, and root corrosion, linked to dead Li formation.

Further analysis of the chemical species in the interphases formed during aging was conducted using XPS measurements (Figures 3f,g, and S36–S41). Compared to the fresh deposits measured immediately after cycling, the C 1s, O 1s, Li 1s, and F 1s spectra change significantly during the aging period of the electrodes in the 1S-5Sol electrolyte, while the interphase components are well-preserved in the interphase formed in the 5S-2Sol electrolyte, showing only slight changes in the spectra related to Li surfaces after aging (Figure S37). This suggests accumulation of organic surface species with C=O groups as reflected by the O 1s spectra, on the surface of the Li-metal deposits, consistent with the EDS mapping results, reflecting dominance of solvents’ reduction products in the interphase formed in the 1S-5Sol electrolyte. In contrast, the XPS spectra of Li deposits in the 5S-2Sol electrolyte reflect a relatively stable surface chemistry. The spectra measured before and after aging are similar, with slight increases in peaks reflecting N–O, SO_*x*_, B–O, and S–N bonds, related to salt anions reduction. This indicates higher stability of the interphase formed on Li-metal deposits in the multisalt electrolytes. The above results demonstrate that the multicomponent inorganic-rich interphase formed on Li-metal anodes in the multisalt (5S-2Sol) electrolytes exhibits superior electrochemical/chemical stability, effectively slowing down corrosion.

### Performance of High-Energy Full Batteries

**Figure 4:**
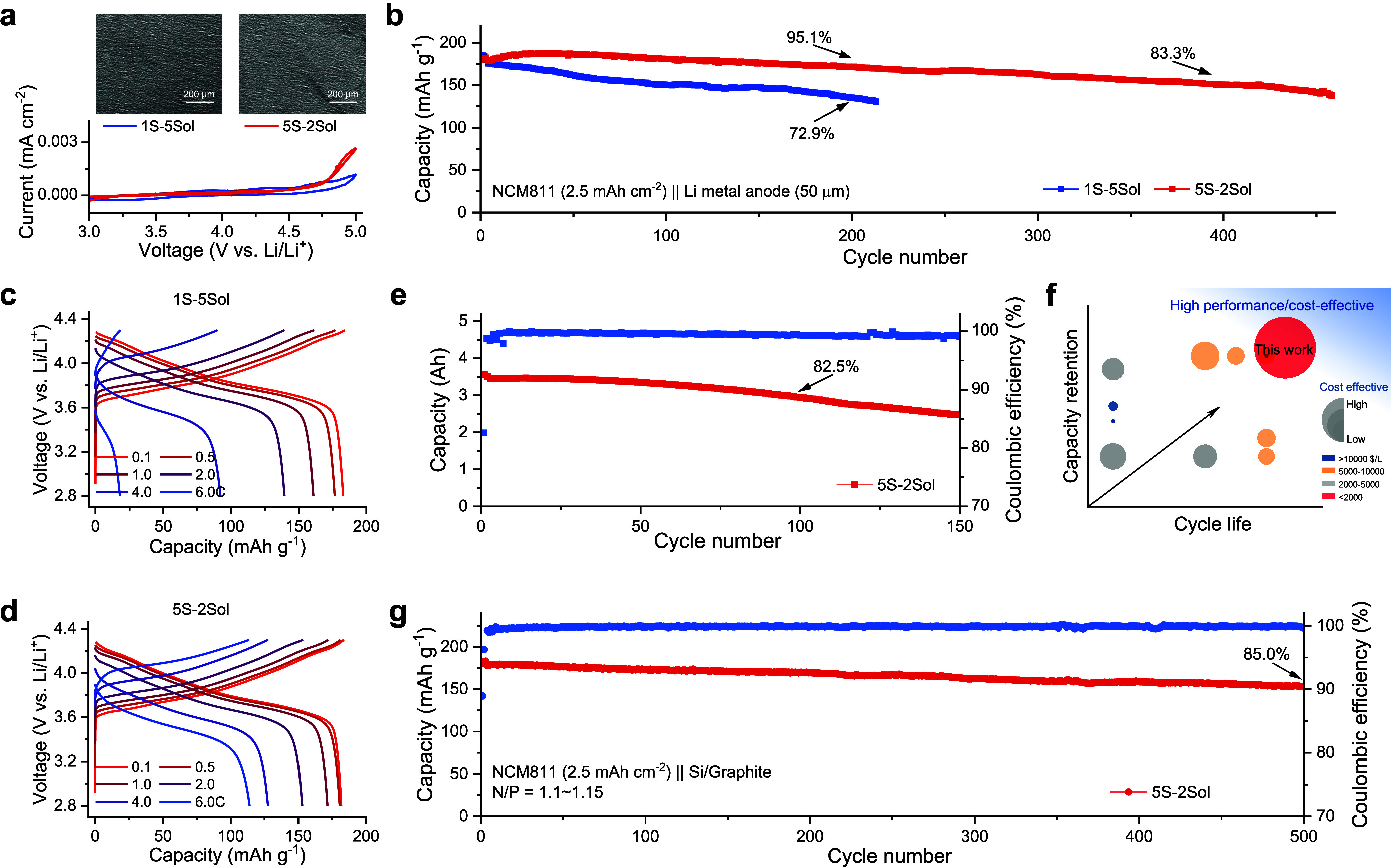
Electrochemical performance. (a) CV curves of Li||Al cells with a scan rate of 0.8 mV s^–1^ from 3.0 to 5.0 V. The insets show the morphologies of the Al current collector after corrosion test. (b) Capacity retention of Li||NCM811 cells that contained different electrolytes cycled between 2.8 and 4.3 V at 0.1 C rate for three cycles and then at 1.0 C rate for the following cycles. (c,d) Galvanostatic charge/discharge curves of Li||NCM811 cells cycled between 2.8 and 4.3 V under various current densities in different electrolytes. (e) Cycling performance of anode-free 3.5 A h pouch cells. Cells were tested with double-side coated NCM811 cathodes and bare Cu foil anodes cycled between 2.6 and 4.4 V at a 0.1 C rate for three cycles and then at a 0.5 C rate for the following cycles. (f) Comparative analysis of electrolyte cost and battery performance related to anode-free cells. The data are based on the representative electrolytes in Table S4. The color scale indicates various cost levels according to Table S7. Bubble size reflects the cost effectiveness, determined by the total cost of each electrolyte relative to the highest cost among all the electrolytes considered. (g) Capacity retention of NCM811||Si/graphite cells cycled between 2.6 and 4.3 V at a 0.1 C rate for the first three cycles and then at a 1.0 C rate for the following cycles. The capacity ratio of the negative over positive electrode was 1.1–1.15. Si/graphite composite anode has a specific capacity of 450 mA h g^–1^.

The interphase properties in combination with high-capacity cathode materials are further evaluated. The oxidation stability of the 5S-2Sol and 1S-5Sol electrolytes is first examined by CV measurements in Li||Al cells. The multisalt 5S-2Sol electrolyte presented an electrochemical stability window up to ∼4.7 V vs Li/Li^+^, as shown in Figure 4a. In addition, the potentiostatic polarization measurements show that the oxidation leakage current of Al current collector in the 5S-2Sol electrolyte reached a minimum value of ∼0.27 μA cm^–2^ after being held at 4.3 V for 8 h, which is lower than ∼0.48 μA cm^–2^ measured for the 1S-5Sol electrolyte (Figure S42). The corrosion of the Al collector in the multicomponent electrolytes was also tested, and both electrolytes demonstrated smooth morphologies of Al foils after polarization, indicating potential compatibility with nickel-rich layered cathodes upon charging (Figure 4a). Subsequently, the electrolytes were evaluated in cells consisting of Li-metal anodes and LiNi_0.8_Co_0.1_Mn_0.1_O_2_ cathodes (Li||NCM811) in the voltage range 2.8–4.3 V, whereas the areal capacity of the NCM811 cathode was 2.5 mA h cm^–2^ and the Li-metal anode was 50 μm thick, close to practical loading. During the initial cycles at a rate of 0.1 C, the cells containing either electrolyte present a similar discharge capacity. However, during long-term cycling, cells with the 5S-2Sol electrolyte showed significantly better stability, resulting in a capacity retention of around 95% after 200 cycles upon cycling at a rate of 1.0 C, and a more than 83% capacity was maintained after 400 cycles (Figure 4b). This outperformed the cells containing the 1S-5Sol electrolyte that exhibited average capacity retention of around 73% after 200 cycles. It is well-known that these Ni-rich NCM type cathodes are also covered by SEI type surface films, namely the cathode electrolyte interphases (CEI) layer that affect pronouncedly their stability. The results show that the 5S-2Sol electrolyte has a better compatibility with the high-voltage cathodes and promotes formation of passivating CEI layer on them. Additionally, the cells with the 5S-2Sol electrolyte show promising rate performance, delivering capacity retentions of approximately 94%, 84.5%, 70%, and 60% at rates of 1.0, 2.0, 4.0, and 6.0 C, respectively, which is larger than the capacity retention of around 90%, 77%, 50%, and 10.5% for the cells containing the 1S-5Sol electrolyte cycled at the same rates (Figures 4c,d and S43). The enhanced performance of the cells containing the 5S-2Sol electrolyte should be attributed to the formation of electrode–electrolyte interphases with enhanced properties, including higher ion-transport kinetics and higher stability.

To further assess the compatibility of the interphase formed in the 5S-2Sol electrolyte upon prolonged cycling, more demanding anode-free pouch cells were assembled and cycled under harsh conditions, with zero excess Li-metal, a high cathode loading of 3.5 mA h cm^–2^, and a relatively small amount of electrolyte with an electrolyte-weight/cathode-capacity ratio (E/C) of ∼2 g (A h)^−1^ (Figures 4e and Figure S44, and Table S3). Even under these challenging conditions, the prototype anode-free pouch cell maintains good cycling stability with a capacity retention of around 82.5% after 100 cycles at a rate of 0.5 C, indicating that these designed electrode–electrolyte interphases hold the key to achieve long-cycling and high-stability high-energy Li batteries, even when employing low-cost electrolytes (Figure 4f and Tables S4–S7). This competitive performance based on commercial carbonate solvent mixtures, under aggressive cycling conditions, further confirms the promising properties of the interphases induced by multisalt electrolytes. To verify its application in high-energy-density Li-ion cells, the electrochemical performance of NCM811||Si/graphite full cells was evaluated using the 5S-2Sol electrolyte. Figure 4g shows the cycling performance of full cells cycled between 2.6 and 4.3 V at a rate of 0.1 C for the first three cycles and 1.0 C for the following cycles. The full cells using the 5S-2Sol electrolyte exhibited a capacity retention of around 94%, 88%, and 85% after 200, 400, and 500 cycles, respectively, with an average CE of ∼99.9%. The rate performance test of the NCM811||Si/graphite full cells results in a capacity retention of around 98%, 86%, 70%, and 52.6% at rates of 0.3, 1.0, 3.0, and 5.0 C, respectively (Figures S45 and S46).

The interfacial structure of the NCM811 cathodes and their surface chemistry in the two types of solutions was studied by microscopic and spectroscopic tools, as described in the Supporting Information Note 3. The CEI layer formed in the 5S-2Sol electrolyte was found to be compact, avoiding detrimental dissolutions of transition metal cations from the cathode side (Figures S47–S50), as described therein. These results of the full cells studies demonstrate that the use of multisalt 5S-2Sol electrolyte dramatically improve the performance (stability, cycling efficiency, and rate capability) of all relevant electrodes of secondary Li batteries (Li-metal, Si/graphite anodes, Ni-rich NCM cathodes), through formation of highly passivating, stable CEI layer on the electrode surface.

## Conclusions

**Figure 5:**
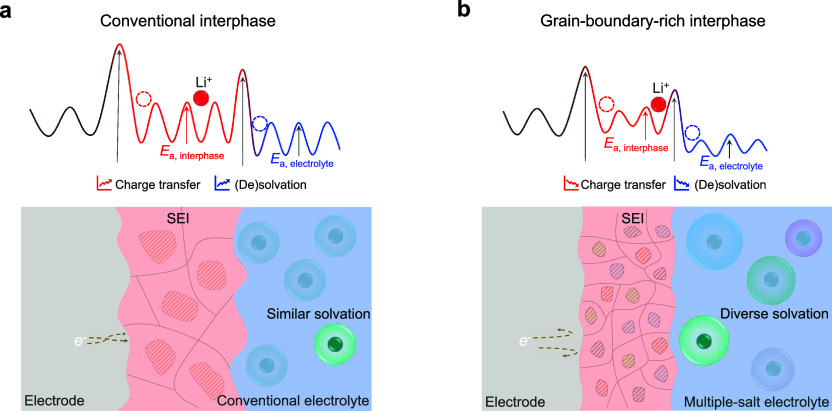
Schematics of Li-ion transport in batteries. The transport of Li-ions across the electrode/electrolyte interphase contributes to the overall battery performance, which is determined by the activation energy in various dominating processes. Li-ion diffusion barrier in the electrolyte, *E*_a,electrolyte_, can be effectively reduced by increasing the diversity in the solvation structure, facilitating liquid-phase Li-ion transport and desolvation at the electrolyte/interphase interface. Li-ion diffusion barrier in solid interphase, *E*_a,interphase_, is influenced by the interphase structure/composition. Unlike conventional interphases (a) formed in typical electrolytes, this new interphase (b) resulting from multianion electrolytes primarily composed of many small polycrystalline inorganic components, which maximizes the percolating pathway, lowering the energy barrier, facilitating desolvation, and enhancing ion transport through the grain-boundary-rich hybrid interphase. Meanwhile, this interphase significantly improves interphase stability against electrochemical/chemical reactions.

In summary, we have developed an approach aimed at creating transformative electrode–electrolyte interphases for advanced batteries. Comprehensive analysis of interphases across various length and time scales revealed that these interphases feature multicomponent, grain-boundary-rich composite microstructures, which result in the remarkably enhanced stability of high-energy-density rechargeable Li battery as well as high Li-ion conductivity throughout the cells (including electrolyte and interphase). Highly effective, protective, and stable interphase can be formed on the reactive battery electrodes through elaboration of an electrolyte system which includes a combination of multiple commercially available salts in conventional alkyl carbonate solvents. The diversity of salts induces finer inorganic grains, homogeneously embedded in the interphase, forming grain-boundary-rich heterogeneous microstructures, effectively facilitating solid–solid ions transport between the electrodes and the formed interphases, as well as solid–liquid ions solvation reorganization between the interphases and the electrolytes (Figure 5a,b). Furthermore, the inorganic-dominated hybrid interphases exhibited higher stability against electrochemical/chemical side reactions with components of the electrolytes, ensuring a prolonged cycle life for anode-free batteries. These findings strongly indicate that our well-designed grain-boundary-rich electrode–electrolyte interphases hold the key to achieving long-cycling and high-stability high-energy Li batteries, even when employing low-cost electrolytes. Therefore, this advancement marks a substantial step toward enhancing the performance and sustainability of high-energy-density secondary batteries, which presents a promising perspective for future developments in the field and beyond.

## Supporting Information Available

Materials, experimental procedures, characterization methods, electrochemical performance, EIS, NMR, cryo-TEM, XPS and XRD characterizations, as well as properties of electrolytes (PDF)

## References

[ref1] Goodenough J. B.; Kim Y. Challenges for Rechargeable Li Batteries. Chem. Mater. 2010, 22 (3), 587–603. 10.1021/cm901452z.

[ref2] Dunn B.; Kamath H.; Tarascon J.-M. Electrical Energy Storage for the Grid: A Battery of Choices. Science 2011, 334 (6058), 928–935. 10.1126/science.1212741. 22096188

[ref3] Li W.; Song B.; Manthiram A. High-voltage positive electrode materials for lithium-ion batteries. Chem. Soc. Rev. 2017, 46 (10), 3006–3059. 10.1039/C6CS00875E. 28440379

[ref4] Wang Q.; Yao Z.; Wang J.; Guo H.; Li C.; Zhou D.; Bai X.; Li H.; Li B.; Wagemaker M.; Zhao C. Chemical short-range disorder in lithium oxide cathodes. Nature 2024, 629 (8011), 341–347. 10.1038/s41586-024-07362-8. 38720041

[ref5] Obrovac M. N.; Christensen L. Structural Changes in Silicon Anodes during Lithium Insertion/Extraction. Electrochem. Solid-State Lett. 2004, 7 (5), A93 10.1149/1.1652421.

[ref6] Wang Q.; Yao Z.; Zhao C.; Verhallen T.; Tabor D. P.; Liu M.; Ooms F.; Kang F.; Aspuru-Guzik A.; Hu Y.-S.; Wagemaker M.; Li B. Interface chemistry of an amide electrolyte for highly reversible lithium metal batteries. Nat. Commun. 2020, 11 (1), 4188 10.1038/s41467-020-17976-x. 32826904 PMC7442789

[ref7] Peled E. The Electrochemical Behavior of Alkali and Alkaline Earth Metals in Nonaqueous Battery Systems—The Solid Electrolyte Interphase Model. J. Electrochem. Soc. 1979, 126 (12), 2047–2051. 10.1149/1.2128859.

[ref8] Peled E.; Menkin S. Review—SEI: Past, Present and Future. J. Electrochem. Soc. 2017, 164 (7), A1703–A1719. 10.1149/2.1441707jes.

[ref9] Wang Q.; Zhao C.; Wang J.; Yao Z.; Wang S.; Kumar S. G. H.; Ganapathy S.; Eustace S.; Bai X.; Li B.; Wagemaker M. High entropy liquid electrolytes for lithium batteries. Nat. Commun. 2023, 14 (1), 440 10.1038/s41467-023-36075-1. 36765083 PMC9918526

[ref10] Langdon J.; Cui Z.; Manthiram A. Role of Electrolyte in Overcoming the Challenges of LiNiO2 Cathode in Lithium Batteries. ACS Energy Lett. 2021, 6 (11), 3809–3816. 10.1021/acsenergylett.1c01714.

[ref11] Markevich E.; Salitra G.; Talyosef Y.; Kim U.-H.; Ryu H.-H.; Sun Y.-K.; Aurbach D. High-Performance LiNiO_2_ Cathodes with Practical Loading Cycled with Li metal Anodes in Fluoroethylene Carbonate-Based Electrolyte Solution. ACS Appl. Energy Mater. 2018, 1 (6), 2600–2607. 10.1021/acsaem.8b00304. 29787244

[ref12] Wang Q.; Wang J.; Heringa J. R.; Bai X.; Wagemaker M. High-Entropy Electrolytes for Lithium-Ion Batteries. ACS Energy Lett. 2024, 9 (8), 3796–3806. 10.1021/acsenergylett.4c01358. 39144807 PMC11320655

[ref13] Whittingham M. S. Electrical Energy Storage and Intercalation Chemistry. Science 1976, 192 (4244), 1126–1127. 10.1126/science.192.4244.1126. 17748676

[ref14] Lu P.; Harris S. J. Lithium transport within the solid electrolyte interphase. Electrochem. Commun. 2011, 13 (10), 1035–1037. 10.1016/j.elecom.2011.06.026.

[ref15] Xu K. Nonaqueous Liquid Electrolytes for Lithium-Based Rechargeable Batteries. Chem. Rev. 2004, 104 (10), 4303–4418. 10.1021/cr030203g. 15669157

[ref16] Aurbach D. Review of selected electrode–solution interactions which determine the performance of Li and Li ion batteries. J. Power Sources 2000, 89 (2), 206–218. 10.1016/S0378-7753(00)00431-6.

[ref17] Peled E.; Golodnitsky D.; Ardel G. Advanced Model for Solid Electrolyte Interphase Electrodes in Liquid and Polymer Electrolytes. J. Electrochem. Soc. 1997, 144 (8), L208–L210. 10.1149/1.1837858.

[ref18] Christensen J.; Newman J. A Mathematical Model for the Lithium-Ion Negative Electrode Solid Electrolyte Interphase. J. Electrochem. Soc. 2004, 151 (11), A1977 10.1149/1.1804812.

[ref19] Raguette L.; Jorn R. Ion Solvation and Dynamics at Solid Electrolyte Interphases: A Long Way from Bulk?. J. Phys. Chem. C 2018, 122 (6), 3219–3232. 10.1021/acs.jpcc.7b11472.

[ref20] Zhang Q.; Pan J.; Lu P.; Liu Z.; Verbrugge M. W.; Sheldon B. W.; Cheng Y.-T.; Qi Y.; Xiao X. Synergetic Effects of Inorganic Components in Solid Electrolyte Interphase on High Cycle Efficiency of Lithium Ion Batteries. Nano Lett. 2016, 16 (3), 2011–2016. 10.1021/acs.nanolett.5b05283. 26889564

[ref21] Wang A.; Kadam S.; Li H.; Shi S.; Qi Y. Review on modeling of the anode solid electrolyte interphase (SEI) for lithium-ion batteries. npj Comput. Mater. 2018, 4 (1), 15 10.1038/s41524-018-0064-0.

[ref22] Janek J.; Zeier W. G. A solid future for battery development. Nat. Energy 2016, 1 (9), 16141 10.1038/nenergy.2016.141.

[ref23] Gurevitch I.; Buonsanti R.; Teran A. A.; Gludovatz B.; Ritchie R. O.; Cabana J.; Balsara N. P. Nanocomposites of Titanium Dioxide and Polystyrene-Poly(ethylene oxide) Block Copolymer as Solid-State Electrolytes for Lithium Metal Batteries. J. Electrochem. Soc. 2013, 160 (9), A1611–A1617. 10.1149/2.117309jes.

[ref24] Zheng J.; Tang M.; Hu Y.-Y. Lithium Ion Pathway within Li_7_La_3_Zr_2_O_12_-Polyethylene Oxide Composite Electrolytes. Angew. Chem., Int. Ed. 2016, 55 (40), 12538–12542. 10.1002/anie.201607539. 27611222

[ref25] Fergus J. W. Ceramic and polymeric solid electrolytes for lithium-ion batteries. J. Power Sources 2010, 195 (15), 4554–4569. 10.1016/j.jpowsour.2010.01.076.

[ref26] Bateman P. C.; Chappell B. W. Crystallization, fractionation, and solidification of the Tuolumne Intrusive Series, Yosemite National Park, California. Geol. Soc. Am. Bull. 1979, 90 (5), 465–482. 10.1130/0016-7606(1979)90<465:CFASOT>2.0.CO;2.

[ref27] Jaupart C.; Tait S. Dynamics of differentiation in magma reservoirs. J. Geophys. Res.: Solid Earth 1995, 100 (B9), 17615–17636. 10.1029/95JB01239.

[ref28] Wang X.; Li Y.; Meng Y. S. Cryogenic Electron Microscopy for Characterizing and Diagnosing Batteries. Joule 2018, 2 (11), 2225–2234. 10.1016/j.joule.2018.10.005.

[ref29] Wang Q.; Zhao C.; Wang S.; Wang J.; Liu M.; Ganapathy S.; Bai X.; Li B.; Wagemaker M. Clarifying the Relationship between the Lithium Deposition Coverage and Microstructure in Lithium Metal Batteries. J. Am. Chem. Soc. 2022, 144 (48), 21961–21971. 10.1021/jacs.2c08849. 36416753 PMC9732870

[ref30] Song J.-H.; Yeon J.-T.; Jang J.-Y.; Han J.-G.; Lee S.-M.; Choi N.-S. Effect of Fluoroethylene Carbonate on Electrochemical Performances of Lithium Electrodes and Lithium-Sulfur Batteries. J. Electrochem. Soc. 2013, 160 (6), A873–A881. 10.1149/2.101306jes.

[ref31] Wang Q.; Zhao C.; Yao Z.; Wang J.; Wu F.; Kumar S. G. H.; Ganapathy S.; Eustace S.; Bai X.; Li B.; Lu J.; Wagemaker M. Entropy-Driven Liquid Electrolytes for Lithium Batteries. Adv. Mater. 2023, 35 (17), 2210677 10.1002/adma.202210677. 36718916

[ref32] Jiao S.; Ren X.; Cao R.; Engelhard M. H.; Liu Y.; Hu D.; Mei D.; Zheng J.; Zhao W.; Li Q.; Liu N.; Adams B. D.; Ma C.; Liu J.; Zhang J.-G.; Xu W. Stable cycling of high-voltage lithium metal batteries in ether electrolytes. Nat. Energy 2018, 3 (9), 739–746. 10.1038/s41560-018-0199-8.

[ref33] Leroy S.; Martinez H.; Dedryvère R.; Lemordant D.; Gonbeau D. Influence of the lithium salt nature over the surface film formation on a graphite electrode in Li-ion batteries: An XPS study. Appl. Surf. Sci. 2007, 253 (11), 4895–4905. 10.1016/j.apsusc.2006.10.071.

[ref34] Forsén S.; Hoffman R. A. Study of Moderately Rapid Chemical Exchange Reactions by Means of Nuclear Magnetic Double Resonance. J. Chem. Phys. 1963, 39, 2892–2901. 10.1063/1.1734121.

[ref35] Ward K. M.; Aletras A. H.; Balaban R. S. A New Class of Contrast Agents for MRI Based on Proton Chemical Exchange Dependent Saturation Transfer (CEST). J. Magn. Reson. 2000, 143 (1), 79–87. 10.1006/jmre.1999.1956. 10698648

[ref36] Columbus D.; Arunachalam V.; Glang F.; Avram L.; Haber S.; Zohar A.; Zaiss M.; Leskes M. Direct Detection of Lithium Exchange across the Solid Electrolyte Interphase by ^7^Li Chemical Exchange Saturation Transfer. J. Am. Chem. Soc. 2022, 144 (22), 9836–9844. 10.1021/jacs.2c02494. 35635564 PMC9185740

[ref37] Zaiss M.; Bachert P. Chemical exchange saturation transfer (CEST) and MR Z-spectroscopy in vivo: a review of theoretical approaches and methods. Phys. Med. Biol. 2013, 58 (22), R221–R269. 10.1088/0031-9155/58/22/R221. 24201125

[ref38] Vinogradov E.; Sherry A. D.; Lenkinski R. E. CEST: From basic principles to applications, challenges and opportunities. J. Magn. Reson. 2013, 229, 155–172. 10.1016/j.jmr.2012.11.024. 23273841 PMC3602140

[ref39] Bryant R. G. The dynamics of water-protein interactions. Annu. Rev. Biophys. Biomol. Struct. 1996, 25 (1), 29–53. 10.1146/annurev.bb.25.060196.000333. 8800463

[ref40] Guivel-Scharen V.; Sinnwell T.; Wolff S. D.; Balaban R. S. Detection of Proton Chemical Exchange between Metabolites and Water in Biological Tissues. J. Magn. Reson. 1998, 133 (1), 36–45. 10.1006/jmre.1998.1440. 9654466

[ref41] McConnell H. M. Reaction Rates by Nuclear Magnetic Resonance. J. Chem. Phys. 1958, 28 (3), 430–431. 10.1063/1.1744152.

[ref42] Woessner D. E.; Zhang S.; Merritt M. E.; Sherry A. D. Numerical solution of the Bloch equations provides insights into the optimum design of PARACEST agents for MRI. Magn. Reson. Med. 2005, 53 (4), 790–799. 10.1002/mrm.20408. 15799055

[ref43] Zaiss M.; Bachert P. Exchange-dependent relaxation in the rotating frame for slow and intermediate exchange – modeling off-resonant spin-lock and chemical exchange saturation transfer. NMR Biomed. 2013, 26 (5), 507–518. 10.1002/nbm.2887. 23281186

[ref44] Lu Y.; Zhao C.-Z.; Huang J.-Q.; Zhang Q. The timescale identification decoupling complicated kinetic processes in lithium batteries. Joule 2022, 6 (6), 1172–1198. 10.1016/j.joule.2022.05.005.

[ref45] Keil P.; Schuster S. F.; Wilhelm J.; Travi J.; Hauser A.; Karl R. C.; Jossen A. Calendar Aging of Lithium-Ion Batteries. J. Electrochem. Soc. 2016, 163 (9), A1872–A1880. 10.1149/2.0411609jes.

