# Supplementary material for: Grain-Boundary-Rich Interphases for Rechargeable Batteries
Source: J Am Chem Soc. 2024 Nov 8;146(46):31778–87. doi: 10.1021/jacs.4c10650 (PMC11583303; doi:10.1021/jacs.4c10650)
Supplement: Supplementary file 1 — ja4c10650_si_001.pdf [file ja4c10650_si_001.pdf]

Supporting Information

Grain-Boundary-Rich Interphases for Rechargeable Batteries

Qidi Wang[1,†]*, Chenglong Zhao[1,†], Xia Hu[2,†], Jianlin Wang[3,†], Swapna Ganapathy[1], Stephen Eustace[4], Xuedong Bai[3], Baohua Li[2], Hong Li[5], Doron Aurbach[6]*, Marnix Wagemaker[1]*

[1]Department of Radiation Science and Technology, Delft University of Technology, Delft 2629 JB, the Netherlands.

[2]Shenzhen Key Laboratory on Power Battery Safety and Shenzhen Geim Graphene Center, Shenzhen International Graduate School, Tsinghua University, Guangdong 518055, China.

[3]State Key Laboratory for Surface Physics, Institute of Physics, Chinese Academy of Sciences, Beijing 100190, China.

[4]Department of Biotechnology, Delft University of Technology, Delft 2629 HZ, the Netherlands.

[5]Key Laboratory for Renewable Energy Institute of Physics, Chinese Academy of Sciences, Beijing 100190, China.

[6]Chemistry Department, BINA−BIU center for nanotechnology & advanced materials, Bar−Ilan University, Ramat Gan 5290002, Israel.

[†]Q.W., C.Z., X.H., and J. W. contributed equally to this work.

*Corresponding author. Email: q.wang-11@tudelft.nl; doron.aurbach@biu.ac.il; m.wagemaker@tudelft.nl.

Supplementary Notes

Supplementary Note 1.

Electrolyte Properties.

The properties of these electrolytes were investigated firstly. Liquid ^{7}Li nuclear magnetic resonance (NMR) spectroscopy was employed to study Li-ion solvation environments (Supplementary Fig. 1), in which the chemical shift represents the shielding of the Li-ions as a consequence of solvation interactions. Compared to the baseline 1S−2Sol electrolyte at -0.36 ppm, increasing the number of solvents or salt species resulted in slightly positive chemical shifts at around -0.34 and -0.32 ppm, respectively. This indicates that Li-ions experience weaker shielding due to a decrease in local electron density, implying altered solvation interactions[1-2]. The 5S−5Sol electrolyte exhibits a larger downfield shift, approximately -0.28 ppm, suggesting that increasing both the number of solvents and salt species further alters the solvation interactions. The solvation structures were examined using Raman spectroscopy (Supplementary Figs. 2−5). Increasing the number of salt species (not the total salt concentration) decreases the amount of Li-ion coordinated solvents, as reflected by the weaker coordinated peak. This explains the increase in ionic conductivities from 8.85 mS cm^{-1} for the baseline 1S−2Sol electrolyte to ~9.34 mS cm^{-1} for the 1S−5Sol electrolyte, and ~9.81 mS cm^{-1} for the 5S−2Sol electrolyte (Supplementary Fig. 6). Li-ion diffusion coefficients measured with pulsed field gradient (PFG) NMR also support the improved mobility in the multi−component electrolytes (Supplementary Fig. 7). This improvement in kinetics can be explained by the increased disorder in the complex liquid phases[3-4], where increasing the number of components leads to weaker solvation interaction and a wider distribution in diffusional barriers due to diverse solvation structures, enhancing diffusional channels via the available percolation network. Tafel plots show the change in charge-transfer kinetics at the electrode interface, where an increase in exchange current densities is observed when more salt/solvent components are introduced (Supplementary Fig. 8). However, compared to the influence of solvents, increasing salt species in the 5S−2Sol electrolyte shows the largest exchange current density of ~0.41 mA cm^{-2}, suggesting different interphase properties. This is evaluated using cyclic voltammetry (CV) measurements in Li||Cu cells (Supplementary Figs. 9 and 10), where the increasing reduction peaks at around 1.4 V indicate that adding various salts allows to

tune the reduction potentials of these multi-component electrolytes, thus affecting the interphase composition and structure.

Supplementary Note 2.

Li-metal Plating/Stripping Morphologies and Li-Metal Loss.

The Li-metal plating/stripping morphologies within different electrolyte chemistry were investigated using scanning electron microscopy (SEM) measurements. In contrast to the highly porous and whisker-like Li-metal deposits in the 1S−5Sol and 1S−2Sol electrolytes (Supplementary Figs. 15 and 16), the Li deposits in the 5S−2Sol electrolyte are more compact and well-connected to the Cu substrate, showing particle sizes up to ~12 μm (Supplementary Fig. 16). After stripping, a porous morphology with some residual dendritic Li was observed for the 1S−5Sol electrolyte, whereas much less Li-metal appeared to be left behind for the 5S−2Sol electrolyte, indicating more complete and homogenous stripping (Supplementary Fig. 17). Denser and better-connected Li-metal deposits are beneficial for electron transport, allowing more efficient stripping and suppress the formation of residual dead Li[5]. Additionally, compact deposits minimize the exposed surface area, leading to less electrolyte decomposition and, in turn, suppressing the formation of porous Li morphologies upon subsequent cycling. This phenomenon was also observed in the 5S−5Sol electrolyte (Supplementary Fig. 15).

Furthermore, operando solid-state ^{7}Li NMR was employed, as a non-invasive method to monitor Li plating/stripping dynamics during electrochemical measurements[6-7], for quantitative and temporal insights into Li-metal deposition behavior. The chemical shifts of different Li resonances can be used to differentiate between metallic Li (~260−280 ppm) and diamagnetic Li species in the SEI/electrolyte (~0 ppm) via the Knight shift[7]. Therefore, these measurements allowed us to quantify the amount of dead Li-metal and SEI after stripping (see methods)[8]. Operando ^{7}Li NMR measurements were performed using a Li anode-free Cu||$LiFePO_4$ battery configuration[8] to evaluate the different electrolytes. Before charging the Cu||$LiFePO_4$ cells, the Li-metal resonance at ~275 ppm is absent (Supplementary Fig. 17a). Upon charging, the Li-metal resonance grows, reaches a maximum at the end of charge and then shrinks during discharge (Supplementary Figs. 17b and 1c). At the end of the discharge, the Li-metal resonance remains visible, signifying that some Li-metal remains, which is referred to as dead Li-metal. The

calculated fractions of the reversible Li-metal, dead Li-metal, and Li in the SEI are shown in Supplementary Fig. 17d. The electrodes with 1S−5Sol electrolyte exhibited a higher fraction of dead Li-metal (~11.3%) and Li species in the SEI (~6.9%). This indicates that the accumulation of dead Li is the primary reason for the lower reversibility observed, which is linked to the porous and whisker-like morphologies observed in the SEM images. In contrast, the electrodes in the 5S−2Sol solutions shows less Li loss towards dead Li-metal and the Li accumulated in the SEI during cycling which consequently increases the CE of the Li deposition/dissolution process, and thus improves the electrodes' capacity retention upon cycling. The Li-metal plating/stripping involves Li ions migration through the SEI that covers the Li-metal, which implies that SEI formation and its steady-state structure are decisive factors in determining the deposited Li-metal morphology and species that are formed in side reactions, which in turn depends on the electrolyte compositions. The higher average CE observed here indicates that decent Li-metal plating/stripping reversibility can be achieved in low-cost alkyl carbonates-based electrolytes under typical cycling conditions, without relying on concentrated electrolytes or fluorinated co-solvents (both options are not cost effective).

Supplementary Note 3.

Studies of The Interphase Films Formed on The NCM811 Cathodes.

The interphase chemistry between the cathode and electrolyte was further investigated using SEM and TEM measurements. NCM811 cathodes cycled in the 1S−5Sol electrolyte were covered by uneven surface layers with a thickness of around 8−16 nm (Supplementary Fig. 47). In contrast, uniform surface layers with a thickness of around 10 nm was formed on the surface of the cycled cathodes in cells containing the 5S−2Sol electrolyte solution. The surface layers formed in the 5S−2Sol electrolyte appears to suppress cracks formation and pulverization of cathode secondary particles, unlike the situation using 1S−5Sol electrolyte solution (Supplementary Fig. 48). The suppression of microstructural degradation of the cathode particles probably reduces side reactions, thereby preventing electrolyte consumption. Furthermore, the chemical composition of surface layers was investigated using electron energy loss spectroscopy (EELS) measurements. Comparing the Ni *K*-edge spectra of the surface layers formed on the cycled NCM811 cathodes in both electrolyte solutions, we can observe Ni dissolution from the cathodes in the 1S−5Sol

electrolyte (Supplementary Fig. 49), which plays a vital role in cathode degradation. The dissolved transition metal cations should migrate to the anode side, resulting in obvious damage to the integrity of their passivating surface films, demonstrating a very detrimental cross-over effect[9], which eventually decreases the cycling stability. In contrast, the surface layers formed in the 5S−2Sol electrolyte were found to be F-rich (Supplementary Fig. 50), which can be responsible for the high-voltage stability of the surface layers[5]. These results demonstrate that the use of multi-salt 5S−2Sol electrolyte solution, through formation of the inorganic-rich interphases on the electrodes, contributes to the formation of homogeneous and stable protective surface layers, thereby enhancing the electrochemical performance of full cells.

Materials and Methods

Materials.

The solvents, ethylene carbonate (EC), dimethyl carbonate (DMC), fluoroethylene carbonate (FEC), propylene carbonate (PC), diethyl carbonate (DEC), ethyl methyl carbonate (EMC), were purchased with battery-grade purity, which was dehydrated with a 4 Å molecular sieve (Sigma-Aldrich) to eliminate the trace water. Lithium hexafluorophosphate ($LiPF_6$), lithium bis(fluorosulfonyl)imide (LiFSI), lithium bis(trifluoromethanesulfonyl) imide (LiTFSI), lithium difluoro(oxalato)borate (LiDFOB) were dried in the vacuum oven of glovebox at 80 ^oC overnight. The suppliers are shown in the table S5 and S6. Lithium nitrate ($LiNO_3$, >99.9%) was purchased from Shenzhen Capchem Technology Co., Ltd and used as received. Lithium metal foils (thickness of 250 µm), Cu foils, and Al foils were purchased from MTI Corporation, and lithium metal foils (50 µm) were purchased from China Energy Lithium Co. Ltd. All lithium metal foils were washed 3 times with DMC solvent before use. Cu foils were immersed in diluted acetic acid for several minutes, subsequently washed with deionized water and acetone three times, separately, then quickly dried in the vacuum oven of the glovebox at room temperature. All the electrolyte solutions were prepared by dissolving the specific amount of different lithium salts in solvents in an Ar-filled glovebox (H_2O < 0.1 ppm, O_2 < 0.1 ppm).

The 1.0 mol L^{-1} (M) $LiPF_6$−EC/DEC solution (1S−1Sol) was prepared by dissolving 1.0 M $LiPF_6$ in the EC/DEC mixing solvents in the volume ratio 1:1. The 1.0 mol L^{-1} (M) $LiPF_6$−EC/DEC/PC/DMC/EMC (1S−5Sol) was prepared by dissolving 1.0 M $LiPF_6$ in the mixing solvents with the equal volume ratio. The 5S−2Sol electrolyte was prepared by dissolving 0.225 M LiFSI, 0.225 M $LiPF_6$, 0.225 M LiDFOB, 0.225 M LiTFSI, and 0.1 M $LiNO_3$ in the EC/DEC mixing solvents in volume ratio 1:1, where $LiNO_3$ was first dissolved in EC solvent at 60−80 ^oC with the assistance of a touch mixer machine, and then the other salts were added into this mixture. The 5S−5Sol electrolyte was prepared by dissolving 0.225 M LiFSI, 0.225 M $LiPF_6$, 0.225 M LiDFOB, 0.225 M LiTFSI, and 0.1 M $LiNO_3$ in the EC/DEC/PC/DMC/EMC mixing solvents in the equal volume ratio, where $LiNO_3$ was first dissolved into EC/PC mixing solvents at 60−80 ^oC with the assistance of a touch mixer machine, and then the other salts and solvents were added to this mixture. Finally, 5% FEC in volume was added to all the electrolytes.

$LiFePO_4$ was obtained from Leneng Technology and the cathodes were prepared by mixing $LiFePO_4$ material, poly(vinylidene difluoride) (PVDF, MTI) binder, and Super P (Alfa Aesar) conductive carbon in a weight ratio of 92:4:4. The resulting slurry was cast on the Al foil then dried at 60 °C for 6 h, followed by drying overnight at 120 °C in a vacuum oven.

$LiNi_{0.8}Co_{0.1}Mn_{0.1}O_2$ (NCM811) was synthesized using the coprecipitation method. A certain amount of alkaline aqueous solution (NH_4OH and NaOH) was poured into deionized water (1.5 L) to form the base solution in a tank reactor under continuous stirring. Then, a 2 M solution of $NiSO_4·6H_2O$, $CoSO_4·7H_2O$, and $MnSO_4·H_2O$ with a molar ratio of 8:1:1 and an aqueous solution of 5 M NH_4OH and 10 M NaOH was added to the base solution in the tank reactor at a steady rate of 8 mL min^{-1}. The coprecipitation temperature was controlled at 50 ^oC, and the pH value was maintained at around 11 by NH_4OH with a stirring speed of 500 rpm under a nitrogen atmosphere. The coprecipitated $Ni_{0.8}Co_{0.1}Mn_{0.1}(OH)_2$ precursor was prepared, then subsequently washed with deionized water and ethanol four times and dried in a vacuum at 120 ^oC for 24 h. The apparent and tap density of $Ni_{0.8}Co_{0.1}Mn_{0.1}(OH)_2$ precursors are 1.88 g cm^{-3} and 2.06 g cm^{-3}, respectively. For the preparation of NCM 811 materials, the as-obtained precursor was mixed with $LiOH·H_2O$ at a molar ratio of 1:1.03; then first heated at 500 ^oC for 5 h and subsequently calcined at 780 ^oC for 12 h in an oxygen atmosphere. After cooling naturally, the obtained material was directly put into an Ar-filled glovebox to prevent any moisture exposure. The NCM811 electrodes were prepared by mixing the active material, Super P, and PVDF binder in the mass ratio of 90: 5: 5 in N-methyl-2-pyrrolidone (NMP) solvent and cast on Al foil and then dried at 60 °C for 6 h, followed by drying in a vacuum oven at 120 °C overnight. X-ray diffraction pattern demonstrates the pure phase of the prepared NCM811 material.

Si/Graphite electrode material was purchased from BTR (China) with a capacity of 450 mAh g^{-1}. The electrodes were prepared by mixing the active material, Super P, and PVDF binder in the mass ratio of 94: 3: 3 in NMP solvent and cast on Cu foil, and then dried at 80 °C for 6 h, followed by drying in a vacuum oven at 120 °C overnight.

Materials characterization.

Morphologies of electrodes were measured by a cold field scanning electron microscope (SEM, HITACH-S4800, SU8010). Elemental composition on the surface of the electrodes was analyzed by X-ray photoelectron spectroscopy (XPS, PHI 5000 VersaProbe II) using a

monochromatic Al *K*α X-ray source with X-ray settings of 100 μm 25 W 15 kV. Peaks were fitted using MultiPak software calibrated with respect to carbon (284.8 eV). The above morphology and composition characterization were performed with cells disassembled after specific cycles in an Ar-filled glovebox and rinsed with pure DMC solvent three times to remove residual electrolyte, followed by drying in a glovebox for several hours at room temperature to remove the residual solvent. For the sample characterizations obtained through continuous electrochemical cycling, the samples were collected immediately after the cells were finished. For the sample characterizations obtained through intermittent electrochemical cycling, the samples were collected after the cells aged for a certain time. Then these electrodes were transferred into the vacuum transfer boxes for measurements to avoid air exposure. Raman spectroscopy was measured by a Micro-laser confocal Raman spectrometer (Horiba LabRAM HR800 spectrometer) equipped with an Olympus BX microscope and an argon ion laser (532 nm) at room temperature. All the electrolytes were hermetically sealed in quartz cuvettes in a glovebox before measurement.

Cryo-transmission electron microscopy (cryo-TEM) characterization.

Conventional and cryo-(S)TEM experiments were performed on a scanning transmission electron microscope (STEM) (JEM-ARM300F, JEOL Ltd.) operated at 300 kV with a cold field emission gun and double Cs correctors. During image acquisition, the corresponding electron dose flux (units of number of electrons per square Angström per second, e^{-} $Å^{-2}$ s^{-1}) was recorded. Conventional STEM images were taken with a dose rate of over 1000 e^{-} $Å^{-2}$ s^{-1} with an exposure time for each image of several seconds. Cryo-TEM images were obtained with an exposure time for each image of around 0.3 s with a built−-in drift correction function using the OneView and K2 cameras. Cryo-TEM images were taken with an electron dose rate of 50−500 e^{-} $Å^{-2}$ s^{-1}. Short-exposure single-frame shots were used to estimate the defocus and make it as close as possible to Scherzer defocus. EELS spectra were acquired on a GIF Quantum camera with a dispersion of 1 eV $channel^{-1}$, utilizing the Dual EELS capability to correct for drift in the low-loss centered on the zero-loss peak and core-loss centered on the *C* K-edge. The EELS spectrum images were carried out with a camera length of 20 mm, and a pixel dwell time of 10 ms. Energy drift during spectrum imaging was corrected by centering the zero-loss peak to 0 eV at each pixel. Elemental maps were computed through a two-window method in a pre-edge window fitted to a power-law background and a post-edge window of 50−200 eV on the core-loss signal. Analysis of the spectra has been

performed in Gatan microscopy suite software. For cryo-TEM sample preparation and transfer, cells were disassembled immediately in an argon-filled glovebox after cycling and then both lithium metal anodes and NCM811 cathodes were rinsed with pure DMC three times to remove lithium salts, followed by drying in the glovebox for one hour at room temperature to remove the residual solvent. During the washing procedure, approximately 10 mL DMC was carefully dropped onto each of the electrodes one time to reduce additional artifacts on the electrodes.

For cryo-TEM preparation of lithium metal anode, a lacey carbon TEM grid was put on a Cu foil working electrode and assembled into Li‖Cu cells in an argon-filled glovebox. The cells were discharged at a constant current density of 1.0 mA cm^{-2} for 15 min, after which the TEM grid was taken out by disassembling the cells for measurement. The TEM grid was carefully transferred into the cryo-TEM holder in the glovebox with a specialized shutter to prevent air exposure and ice condensation onto the sample introducing any side reactions. Once the cryo-TEM holder was transferred into the TEM column, the temperature was maintained at around -170 ^{o}C using liquid nitrogen.

^{7}Li chemical exchange saturation transfer (CEST).

The measurements of ^{7}Li CEST were performed on a wide-bore Bruker Ascend 500 system equipped with a NEO console with a magnetic field strength of 11.7 T and Larmor frequency for ^{7}Li of 194.37 MHz. The saturation pulse of the CEST experiment lasted for 0.5 s and the RF amplitude was from 500 Hz to 3500 Hz. The saturation offset frequency ω was in the range of +500 ppm to -500 ppm with respect to the Li-metal resonance, where the probe tuning was stable over the entire range. The recycle delay was set to 8 s in all experiments to avoid the influence of RF heating. The longitudinal and transverse relaxation rates were determined for the lithium dendrites and the electrolyte using inversion-recovery and Carr-Purcell-Meiboom-Gill experiments, respectively. The spectra were initially processed using Bruker Topspin software including phase and baseline corrections. Further processing and analysis of the data were carried out in MATLAB (version 2022a).

The CEST effect was analyzed using a Z-spectrum, generated with the normalized Li-metal signal intensity, $Z(\Delta\omega)$, as a function of the saturation frequency $\Delta\omega$[10-11]. $Z(\Delta\omega)$ is given by the following equation:

$$Z(\Delta\omega) = \frac{S_{\text{metal}}(\Delta\omega)}{S_{\text{metal}}},$$

where $\Delta\omega$ is the offset with respect to the Li-metal frequency ($\Delta\omega = \omega_{\text{sat}} - \omega_{\text{metal}}$), and $S_{\text{metal}}(\Delta\omega)$ is the intensity of the Li-metal signal with saturation on $\Delta\omega$.

When irradiating the Li metal at around 265 ppm, the signal disappears due to direct saturation (DS). This frequency is assigned to 0 ppm in Z-spectra. This DS may interfere with the detection of CEST effects, which is addressed by employing the symmetry of the DS through a so-called magnetization transfer ratio (MTR) asymmetry analysis[12], using the following equation:

$$\text{MTR}_{\text{asym}} = \frac{S_{\text{metal}}(-\Delta\omega) - S_{\text{metal}}(\Delta\omega)}{S_0},$$

where S_0 corresponds to the Li-metal signal without saturation.

The Z-spectra of the four electrolyte systems were analyzed using the two pools exchange model with abundant pool (Li-metal pool) and rare pool (SEI pool), where the f_s corresponds to the ratio between the SEI and Li-metal pool concentrations. Separating the exchange rate from the concentration of the exchanging pools requires the simultaneous fitting of multiple *Z*-spectra[13-15]. Therefore, the Z-spectra acquired with varying saturation amplitude B_1 were fitted with two-pool Bloch-McConnell (BMC) differential equations which are six coupled first-order linear differential equations[16-18]:

$$\frac{d\vec{M}}{dt} = A\vec{M} + \vec{C},$$

$$A = \begin{bmatrix} L_m - f_s K & K \\ f_s K & L_s - K \end{bmatrix},$$

and *m* and *s* stand for Li metal and SEI, and $\vec{M}$ is the magnetization vector described by:

$$\vec{M} = \left(M_{m,x} + M_{m,y} + M_{m,z} + M_{s,x} + M_{s,y} + M_{s,z}\right)^T,$$

$$L_i = \begin{pmatrix} -R_{2i} & -\Delta\omega_i & 0 \\ \Delta\omega_i & -R_{2i} & \omega_1 \\ 0 & -\omega_1 & -R_{1i} \end{pmatrix},$$

$$K = \begin{pmatrix} k_s & 0 & 0 \\ 0 & k_s & 0 \\ 0 & 0 & k_s \end{pmatrix},$$

$$\vec{C} = \left(0,0,R_{1m}M_{m,0},0,0,R_{1s}M_{s,0}\right)^T,$$

with i = m,s, $\omega_1 = \gamma B_1$ where γ is the nuclear gyromagnetic ratio and B_1 the saturation amplitude, $\Delta\omega_i$ is the saturation frequency and $k_s = k_{SEI-metal}$. $\Delta\omega$ represent the frequency offset relative to the Larmor frequency of Li metal.

Finally, the equation was fitted with an analytical solution, and Lorentzian line shape for both pools[18-19], and the fitting parameters and boundaries are shown in table S1. The value of f_S was fixed to 0.01 with all other parameters free and the results of this fit are summarized in table S2.

The samples of deposited Li metal in various electrolytes for Li-exchange detection were prepared in Li||Cu CR2032 coin cells with a PTFE ring (thickness around 0.5mm) on the Cu side and a Celgard 2500 separator on the Li metal side. A current density of 1 mA cm^{-2} and a capacity of 6 mAh were used for Li-metal plating on the Cu current collector. After Li plating, the Li||Cu cells were disassembled, and Li-metal deposition was transferred into a 4 mm rotor together with 50 ul corresponding electrolyte to keep the in-situ situation in an Ar-filled glove box (H_2O < 0.1 ppm, O_2 < 0.1 ppm). Then the rotor was sealed with a Vespel cap and taken for nuclear magnetic resonance (NMR) characterization.

Liquid NMR characterization.

Liquid NMR spectra were recorded with an Agilent 400 MHz DD2 NMR spectrometer with a 5 mm ONE NMR Probe at room temperature, which worked at 155.5 MHz on ^{7}Li. The chemical shift values are given in ppm. ^{7}Li chemical shift was referenced to the standard solution: 1 M LiCl in D_2O for ^{7}Li (0 ppm). The external standard solutions were sealed into WILMAD coaxial insert tubes and inserted into the 5−mm KONTES tubes with electrolytes and sealed with PTFE caps. Mestrelab Research Mnova software was used for data processing.

Solid-state NMR characterization.

Operando solid-state NMR measurements were conducted on a wide-bore Bruker Ascend 500 system equipped with a NEO console in magnetic field strength of 11.7 T and a ^{7}Li resonance frequency of 194.37 MHz using a solenoidal Ag-coated Cu coil. Operando static ^{7}Li NMR measurements were performed using an automatic-tuning-and-matching probe (ATM VT X operando WB NMR probe, NMR Service) at room temperature which can allow for an automatic recalibration of the NMR radio-frequency (RF) circuit during an operando electrochemistry experiment. A highly shielded wire with low-pass filters was attached to the probe for

electrochemical measurement, which could minimize the interferences between NMR and the electrochemistry circuit. Single-pulse with a π/2 pulse of 4 μs and recycle delay of 1.0 s was applied to acquire the 1D static spectrums. A recycle delay of three times T_1 was used each time, where T_1 was determined using saturation recovery experiments. The electrochemical cell was simultaneously controlled by a Maccor battery testing system. A plastic capsule cell made out of polyether ether ketone (PEEK) was used for the operando NMR experiments. The cells were assembled using $LiFePO_4$ cathode (areal capacity is 2.0 mAh cm^{-2}) and Cu foils as working and counter electrodes with both a piece of Celgard and a piece of Glass fiber (Whatman GF/A) as a separator. Before measurements, the assembled cells were rested for 2 h in the glove box. The operando capsule cell was aligned in an Ag-coated Cu coil with $LiFePO_4$ and Cu foil electrodes oriented perpendicular to B_0 and parallel concerning the B_1 field. During the static ^{7}Li NMR measurements, the cells were charged to the capacity of 1 mAh cm^{-2} at a current density of 0.5 mA cm^{-2}. A charge cut-off capacity of 1 mAh cm^{-2} was used for lithium metal plating on Cu foils and a discharge cut-off voltage of 2.0 V for stripping. During the charge-discharge process, NMR spectra were continuously acquired. The chemical shift of ^{7}Li was referenced to 1 M aqueous solution of LiCl at 0 ppm. The spectra were processed using the Bruker Topspin software, with the automatic phase and baseline correction. Mestrelab Research Mnova software was used for data processing and analysis.

The quantification of the capacity is based on the Li-metal integrated intensity ratio and the electrochemical CE[8, 20]. The SEI formation capacity in the first cycle can be estimated from the dead Li by NMR and the Coulombic efficiency (*CE*) from the electrochemistry. The capacity loss (*CL*) in the first cycle is defined as:

$$CL = C_{plating} - C_{stripping}.$$

The coulombic efficiency is calculated as follows:

$$CE = \frac{C_{stripping}}{C_{plating}},$$

where the $C_{plating}$ is the full plating capacity (1mAh cm^{-2} in this work), assuming no side reactions.

Then the capacity loss *CL* in the first cycle can be rewritten as:

$$CL = C_{plating} \times (1 - CE).$$

CL includes the capacity loss from dead Li formation ($C_{Dead\ Li}$) and capacity loss from SEI formation (C_{SEI}) in electrochemical measurements as follows:

$$CL = C_{Dead\ Li} + C_{SEI}.$$

$C_{Dead\ Li}$ is estimated from the following equation:

$$C_{Dead\ Li} = (C_{Plating} - C_{SEI}) \times \frac{I_{stripping}}{I_{plating}},$$

where $\frac{I_{stripping}}{I_{plating}}$ is the ratio of the integrated intensity of the Li metal at the end of the 1st discharge to that measured at the end of the 1st charge. Therefore, CL can be calculated:

$$CL = \left(C_{Plating} - C_{SEI}\right) \times \frac{I_{stripping}}{I_{plating}} + C_{SEI}.$$

Time-of-flight secondary ion mass spectrometry (TOF-SIMS) characterization.

The TOF-SIMS characterization was carried out on PHI nanoTOF II (ULVAC-PHI, Japan), with a Bi^{3++} beam (30 kV, 2 nA) used as the primary beam to detect the samples, and the sputter etching was performed using an Ar^{+} beam (3 kV, 100 nA) to obtain the desired depth profile. The area of analysis was 60 μm × 60 μm, while the sputtering area was 400 μm × 400 μm. In the process of sample transfer, a special transfer vessel is used, which can directly transfer the sample from the glove box to the TOF-SIMS vacuum chamber without being exposed to the ambient air. The analysis chamber is maintained in an ultra-high vacuum with pressures below 2×10^{-9} mbar. The samples were prepared on Li-metal deposits after twenty cycles at 0.5 mA cm^{-2} for 2 h in different electrolytes. After the cycles were complete, the samples were collected immediately in an Ar-filled glovebox and rinsed with pure DMC solvent three times to remove residual electrolyte, followed by drying in a glovebox for several hours at room temperature to remove the residual solvent. TOF-DR software was used for data analysis.

Electrochemical measurements.

Electrochemical cycling tests of all batteries were based on CR2032 coin cells assembled in an Ar-filled glovebox (H_2O < 0.1 ppm, O_2 < 0.1 ppm) with Celgard 2500 separator and tested at room temperature, unless stated otherwise. 70 μL electrolytes were injected into each coin cell for comparison. All coin cells were tested using multi-channel battery testing systems (Land CT2001A or Lanhe G340A) at room temperature. Symmetric Li||Li cells were assembled to study the cycling stability under different current densities with electrolytes. 15.6 mm diameter lithium metal foils

with 250 μm thickness were used as both the working and counter electrodes. For Li‖Cu cells, 14 mm diameter lithium metal foils were used as the reference, while 16 mm Cu foils were used as a working electrode with an effective area for lithium deposition of 1.54 cm^2. During cycles, a capacity of 1 mAh cm^{-2} lithium was deposited on Cu foils at a current density of 0.5 mA cm^{-2} and then stripped to a cut-off voltage of 1.0 V vs. Li/Li^+. The electrochemical cycling performance of NCM811 with an areal capacity of ~2.5 mAh cm^{-2} was tested with lithium metal foils with a thickness of 50 μm as the counter electrode. Full cell electrochemical cycling performance of NCM811||Si/Graphite was tested between 2.6−4.3 V. Cells were cycled under a 0.1C rate for three cycles before cycling at a 1.0C rate. The areal capacity of NCM811 cathode used in full cell is ~2.5 mAh cm^{-2}, and the capacity ratio between the anode (the negative electrode) and cathode (the positive electrode), known as N/P ratio, is around 1.1~1.15.

Cyclic voltammetry (CV) of Li‖Cu cells with various electrolytes was conducted at a scan rate of 0.8 mV s^{-1} from -0.1 to 2.5 V vs. Li/Li^+. CV of Li‖Al cells with various electrolytes was conducted at a scan rate of 0.8 mV s^{-1} from 3.0 to 5.0 V vs. Li/Li^+.

Electrochemical impedance spectra (EIS) of the symmetric cells were collected on an Autolab (PGSTAT302N) in the frequency range of 0.1 Hz–1 MHz with a potential amplitude of 10 mV.

The distribution of relaxation times (DRT) was applied to the investigations of the individual relaxation processes occurring in the system and their corresponding relaxation frequencies based on the EIS data using the expression[21]

$$Z_{DRT}(\omega) = R_0 + \int_{-\infty}^{+\infty} \frac{\gamma}{1+i\omega\tau} d(\ln\tau),$$

where R_0 is the ohmic resistance, γ is distribution function that describes the relaxation time, ω is the angular frequency and τ is the relaxation time.

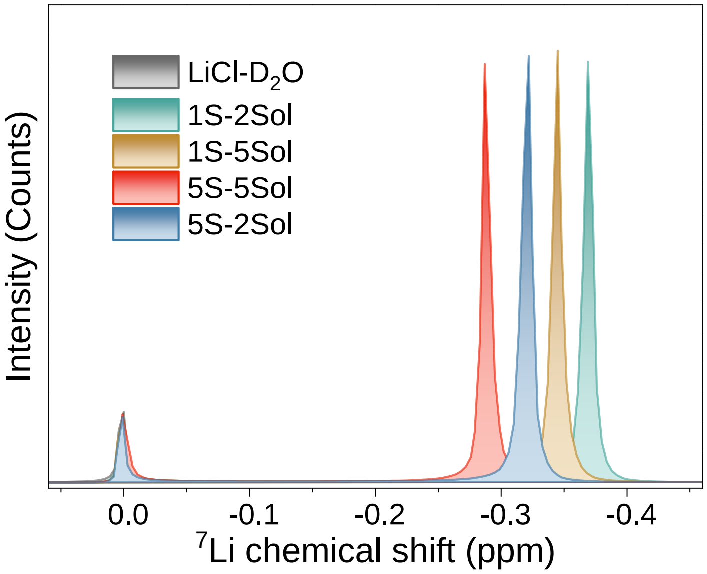

Supplementary Fig. 1. Liquid ^{7}Li nuclear magnetic resonance (NMR) spectra of electrolytes. 1 M LiCl in D_2O is used as a reference.

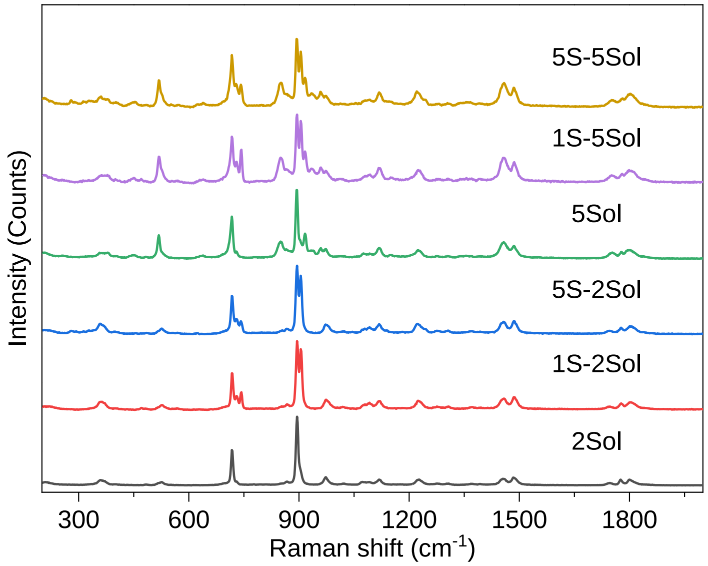

Supplementary Fig. 2. Raman spectra of different electrolytes. The solvent mixtures are shown as a reference.

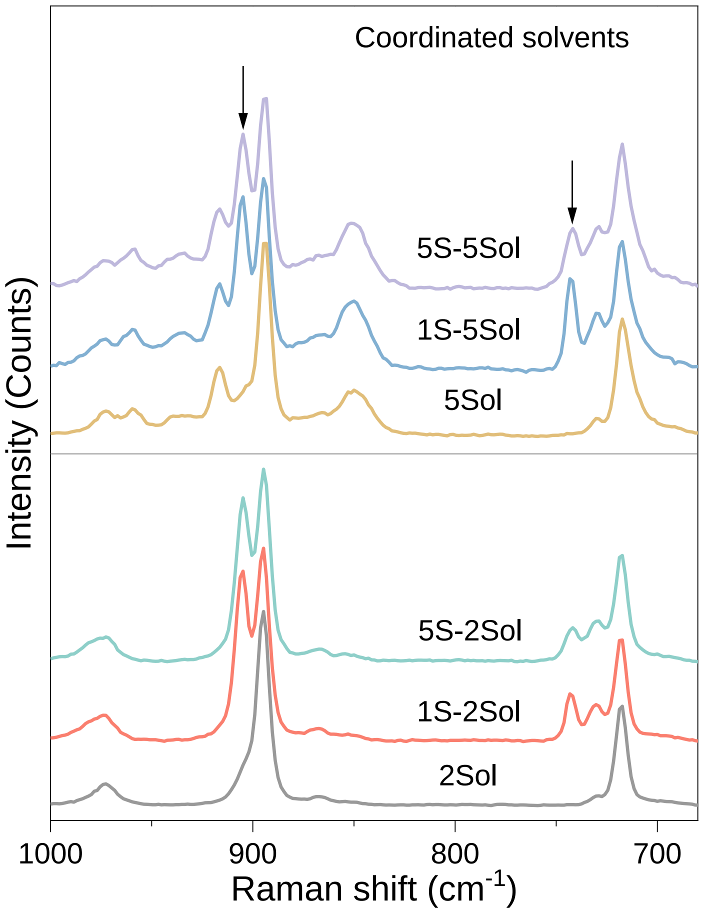

Supplementary Fig. 3. Raman spectra of solvents and electrolyte solutions. The black arrows mark the peaks that represent the Li ions coordinated to solvents.

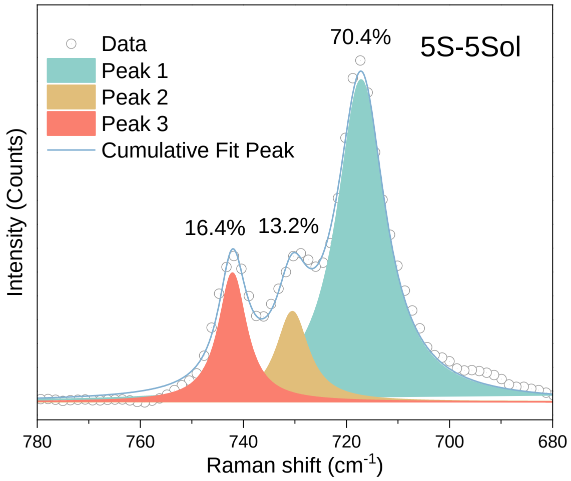

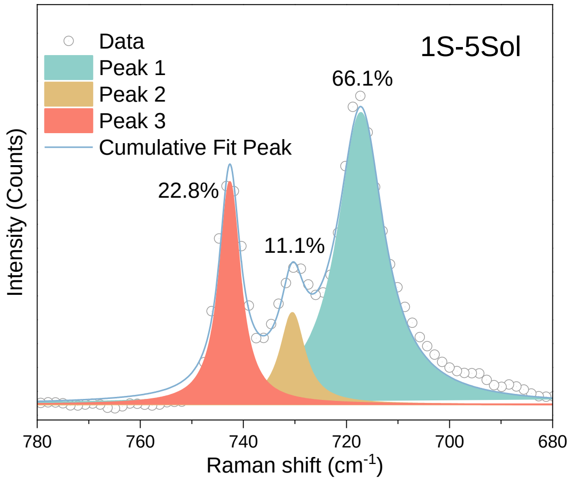

Supplementary Fig. 4. Raman spectra for different electrolyte solutions. The deconvoluted Peak 3 represents the Li-ion coordination to solvents. In the same solvent composition, increasing the variety of salts can decrease the number of coordinated Li-ions to solvents.

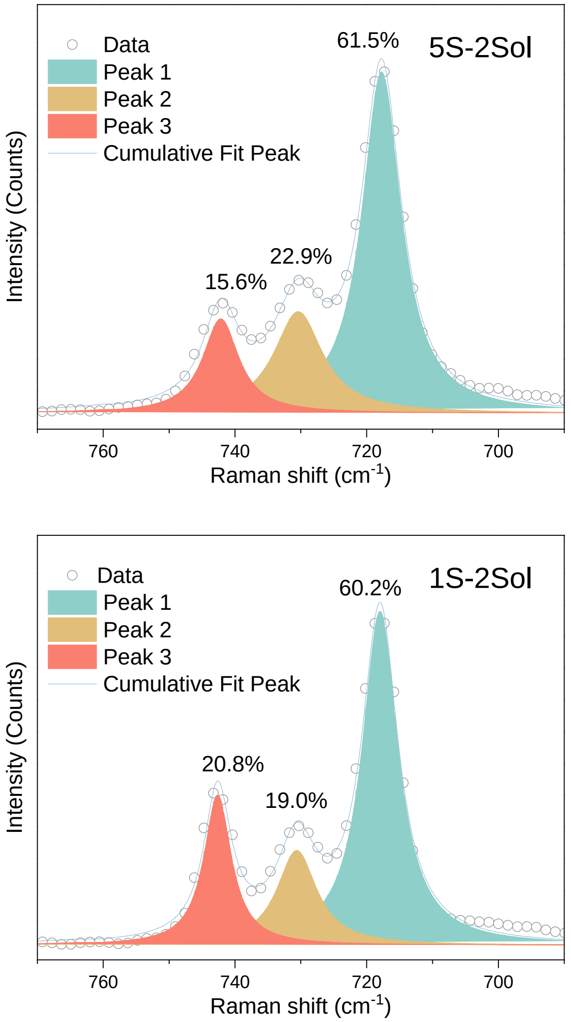

Supplementary Fig. 5. Raman spectra for different electrolyte solutions. The deconvoluted peak 3 represents the Li-ion coordination to solvents. In the same solvent composition, increasing the variety of salts can decrease the number of coordinated Li-ions to solvents.

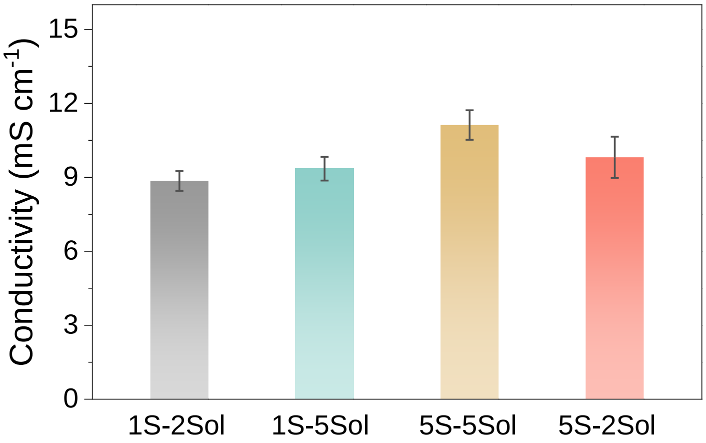

Supplementary Fig. 6. Li-ion conductivity of different electrolyte solutions. The test temperature was 25 ^{o}C.

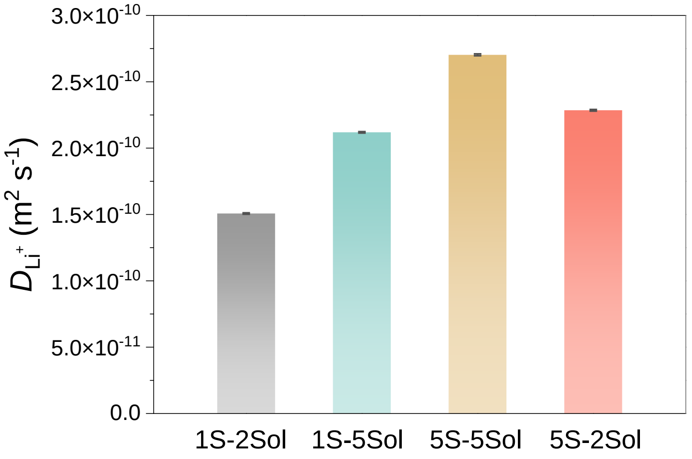

Supplementary Fig. 7. Li-ions diffusion in different electrolyte solutions from ^{7}Li pulsed field gradient (PFG) NMR spectroscopy. The test temperature was 25 ^{o}C.

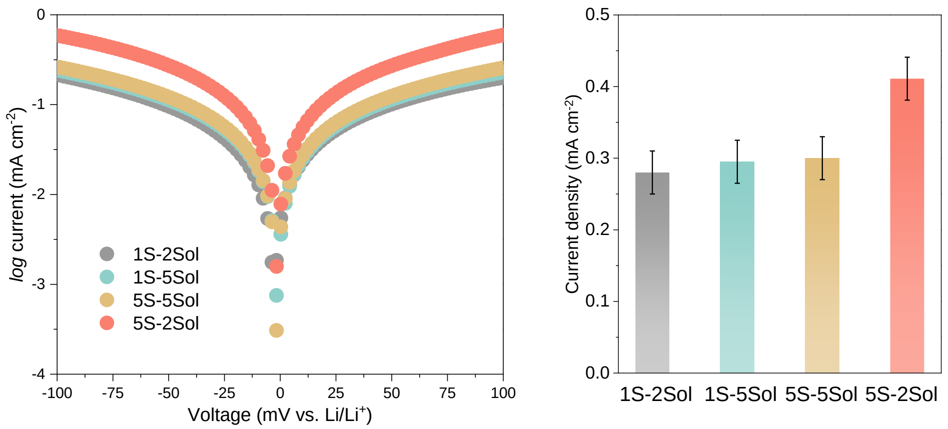

Supplementary Fig. 8. Exchange current density from Tafel plots. Left: Galvanostatic Li plating/stripping in Li||Li cells in different electrolytes; right: the exchange current density was obtained from the linear fitting of the Tafel curves.

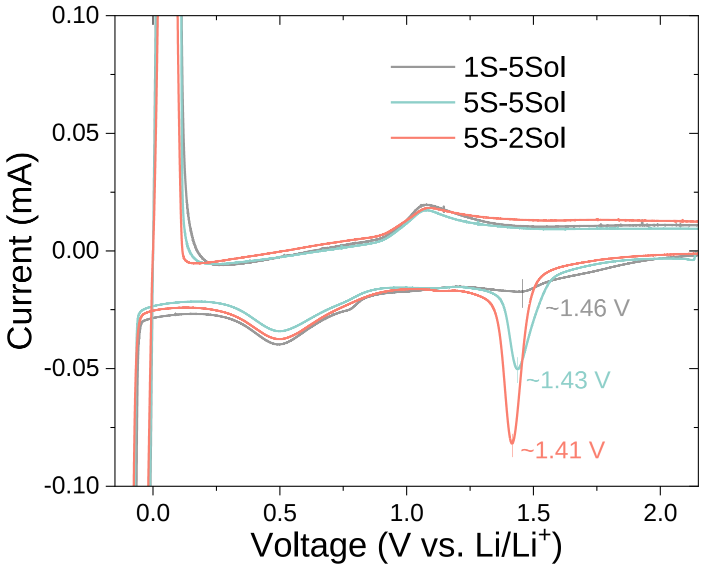

Supplementary Fig. 9. Cyclic voltammetry (CV) curves of Li||Cu cells. The measurements were carried out at a scan rate of 0.8 mV s^{-1} from -0.1 to 2.5 V vs. Li/Li^{+} for different electrolytes.

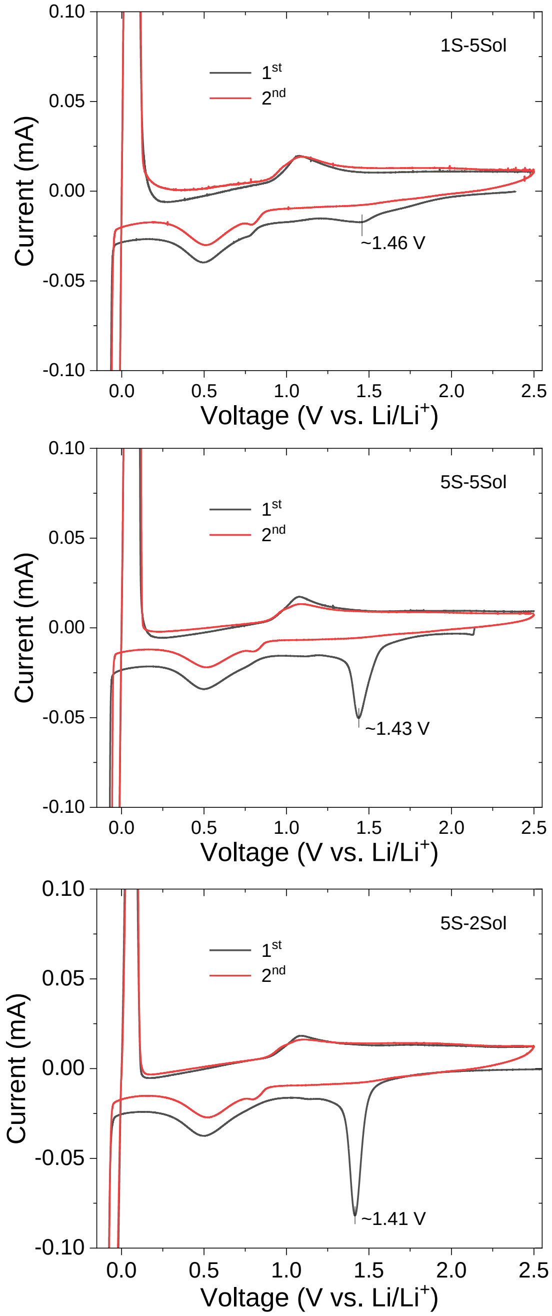

Supplementary Fig. 10. CV curves of Li||Cu cells. The measurements were carried out at a scan rate of 0.8 mV s^{-1} from -0.1 to 2.5 V vs. Li/Li^+ for different electrolytes.

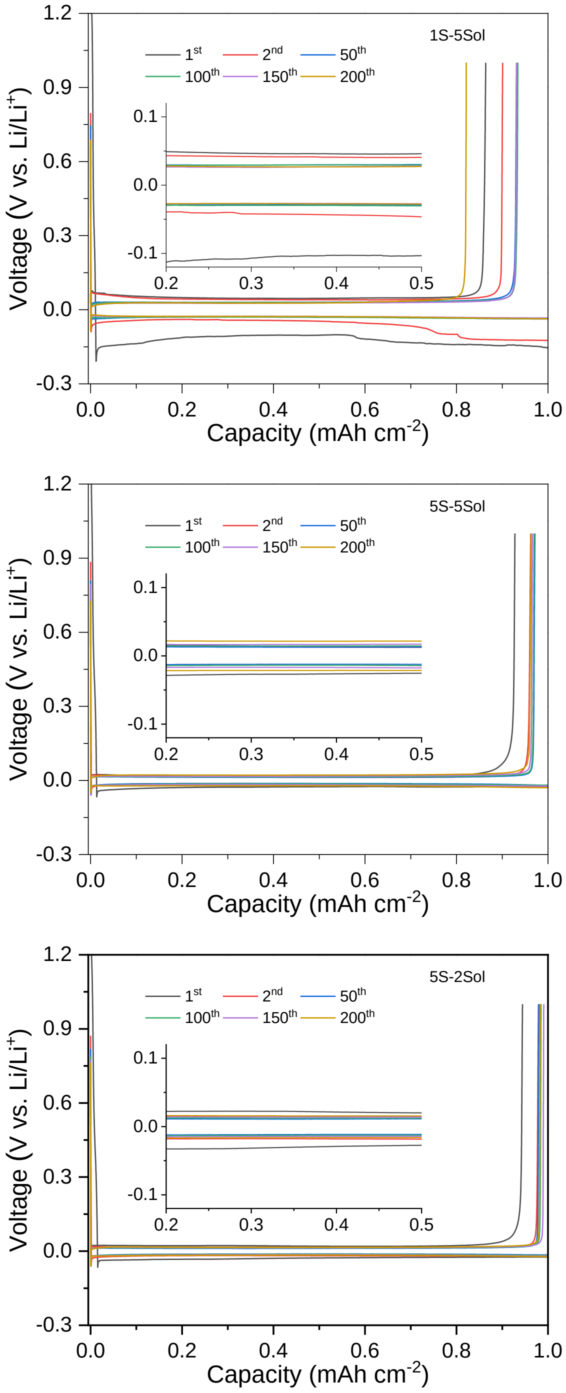

Supplementary Fig. 11. Galvanostatic Li plating/stripping profiles of Li||Cu cells. Cells were tested at the continuous cycling at 0.5 mA cm^{-2} for 2 h (1.0 mA h cm^{-2}). The inserts show the zoomed-in voltage curves of selected cycles.

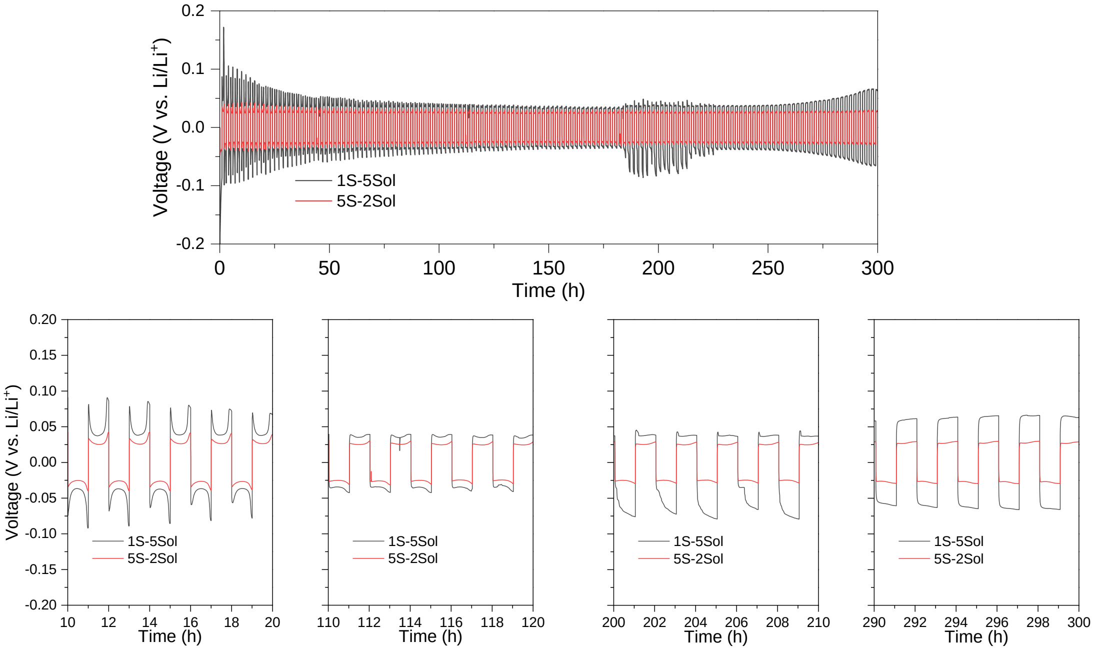

Supplementary Fig. 12. Galvanostatic lithium plating/stripping in Li||Li cells. Cycling of Li||Li cells was tested in different electrolytes at 1.0 mA cm^{-2} to a capacity of 1.0 mAh cm^{-2} and the overpotential of Li||Li cells in different electrolytes at different stages of cycling is shown. The 5S−2Sol electrolyte displays lower overpotentials of ~22 and ~43 mV at a current density of 1 and 3 mA cm^{-2}, outperforming the 1S−5Sol electrolyte of ~42 and ~70 mV, respectively.

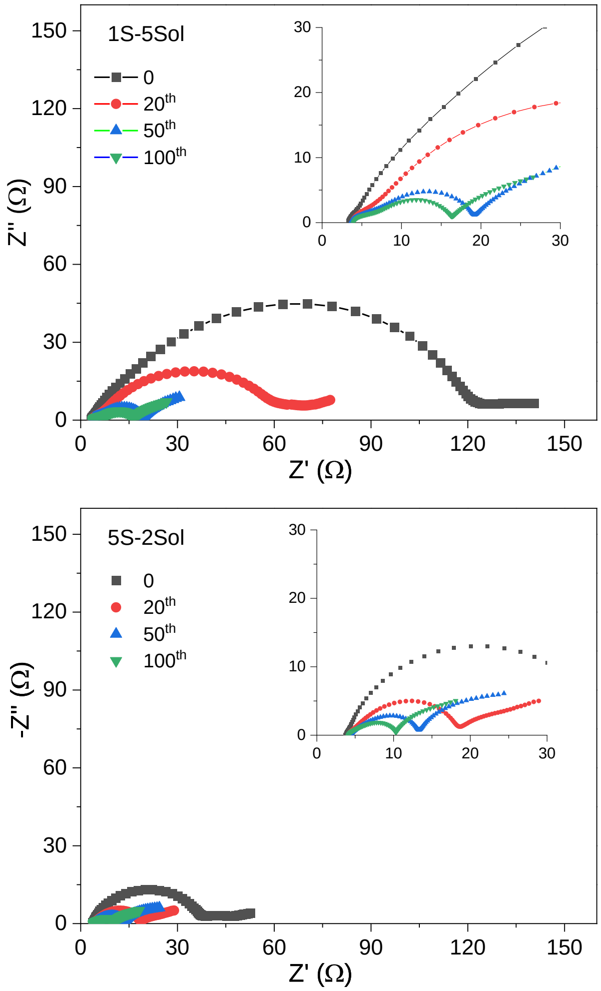

Supplementary Fig. 13. Electrochemical impedance spectra (EIS) presented as Nyquist plots of Li||Li symmetric cells with different electrolyte solutions (indicated in the charts). The Li||Li symmetric cells were cycled under a current density of 1 mA cm^{-2} with each plating/stripping time of 1 h.

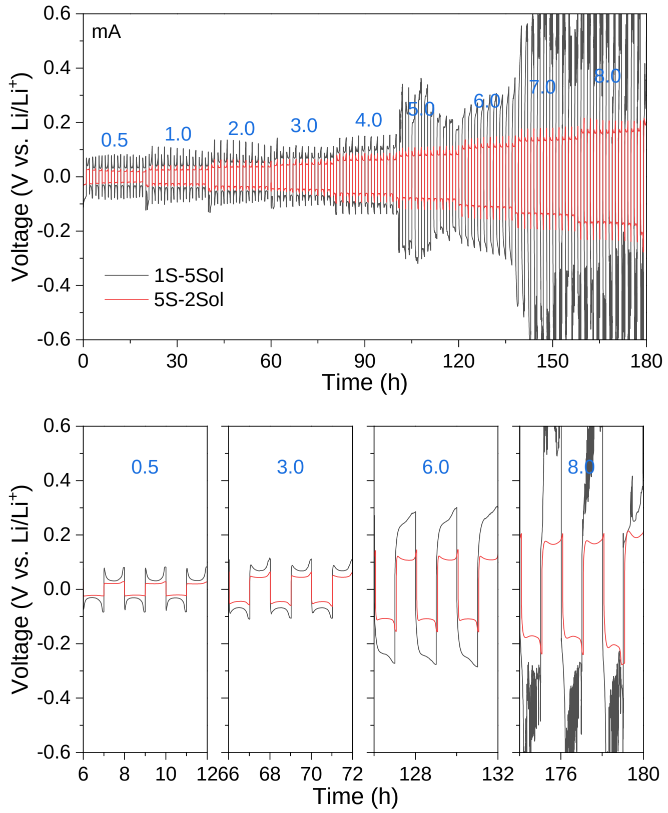

Supplementary Fig. 14. Rate profiles for symmetric cells with different electrolyte solutions. Li||Li cells were tested at a current density from 0.5 to 5.0 mA cm^{-2} with each plating/stripping time of 1 h. When the current density was increased to 5.0 mA cm^{-2}, a short circuit occurred for the cells using the 1S−5Sol electrolyte. In contrast, the 5S−2Sol electrolyte can support the cells to cycle at a higher current density of 8.0 mA cm^{-2} (Supplementary Fig. 14), demonstrating the competitive reaction kinetics of this multi-salt electrolyte. This is consistent with the lower electrochemical impedance as observed during the extended cycles (Supplementary Fig. 13).

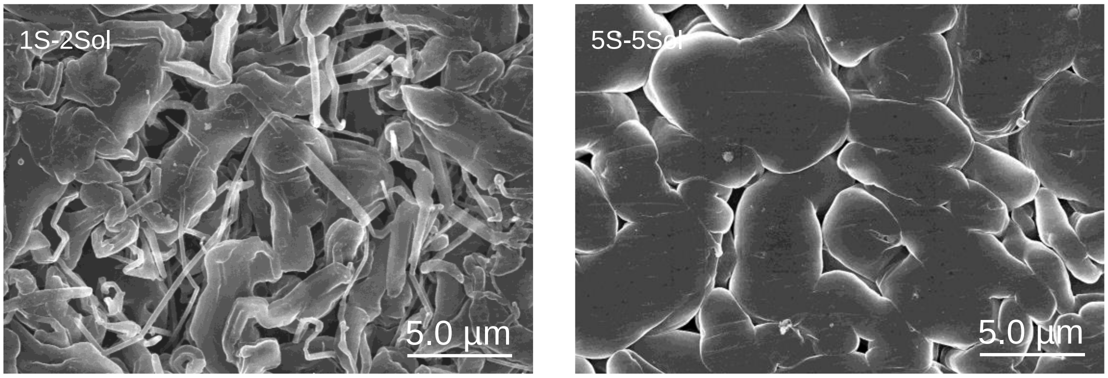

Supplementary Fig. 15. Scanning electron microscope (SEM) images of Cu foil after Li-metal plating in 1S−2Sol and 5S−5Sol electrolytes. Cells were cycled at a current density of 0.5 mA cm^{-2} to 1 mAh cm^{-2} in different electrolytes for ten cycles.

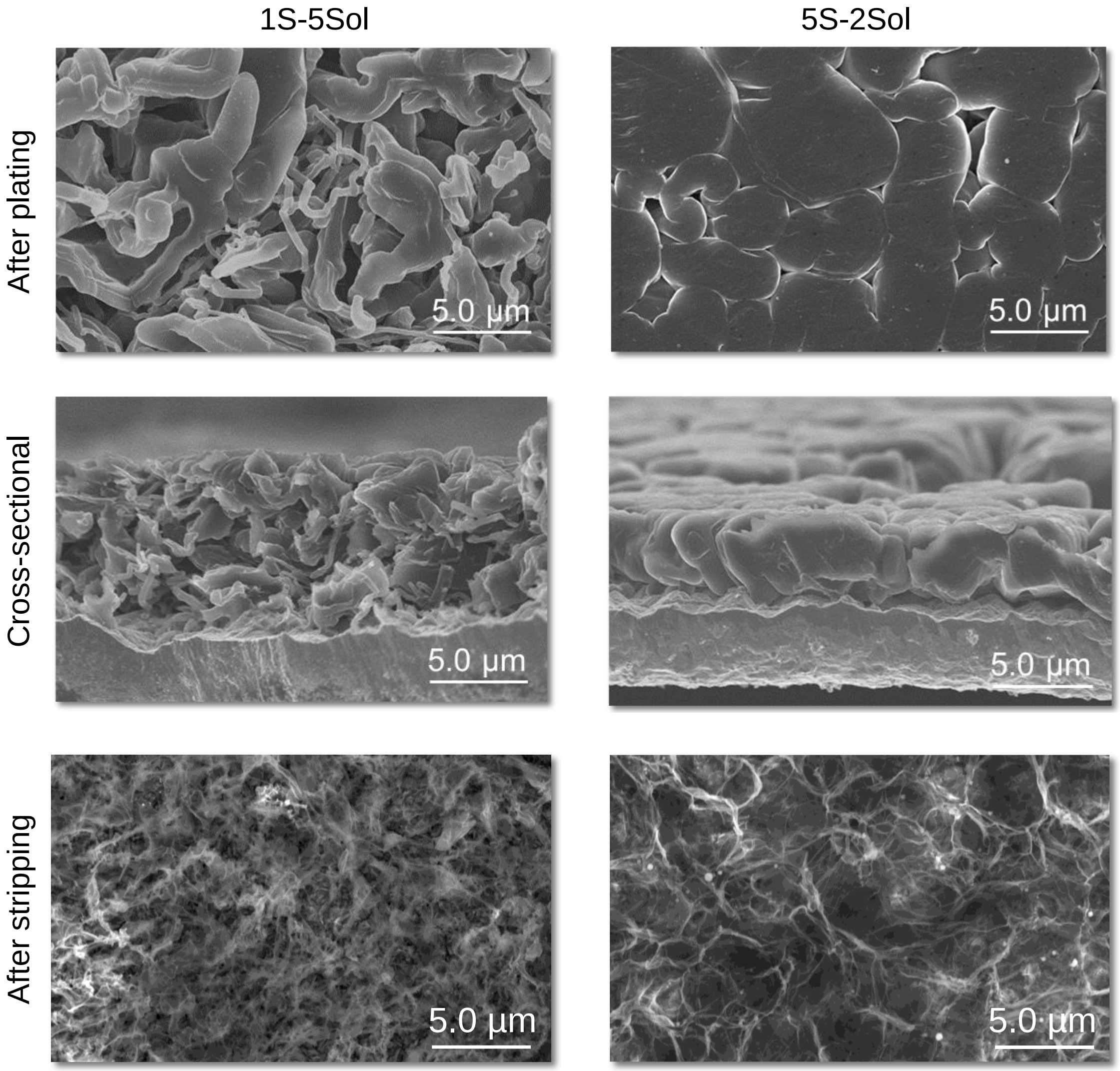

Supplementary Fig. 16. SEM images of Cu foil after Li metal plating and stripping. Cells were cycled at a current density of 0.5 mA cm^{-2} to 1 mAh cm^{-2} in different electrolytes for ten cycles.

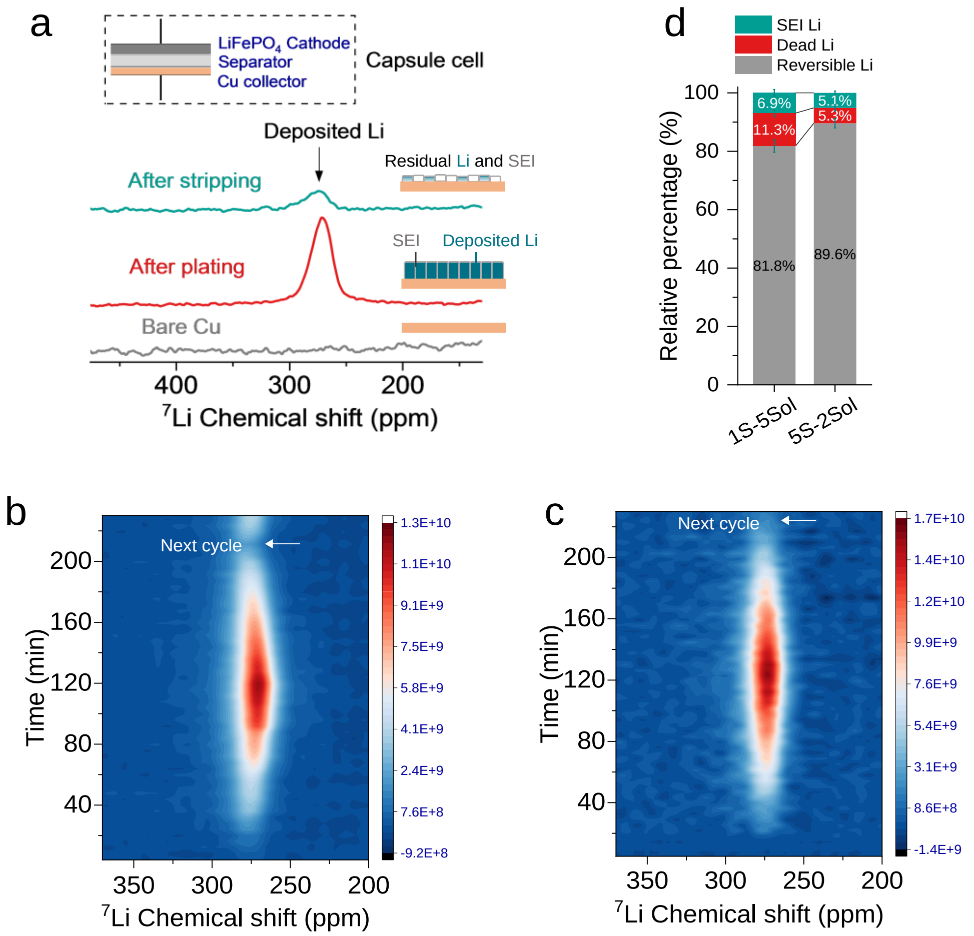

Supplementary Fig. 17. ^{7}Li solid-state NMR spectra after different states during cycling. a, Schematics of operando ^{7}Li NMR measurement using Cu||$LiFePO_4$ cells. Operando ^{7}Li NMR measurement during Cu||$LiFePO_4$ cells cycling with **b,** 1S−5Sol with **c,** 5S−2Sol electrolytes at a current density of 0.5 mA cm^{-2} for 2 h. The arrow indicates the end of this cycle. **d,** Quantifying Li species, including Li species in SEI (blue bars), reversible Li metal (grey bars), and dead Li metal residual (red bars) species, derived from the Li metal integrated intensity ratio and the electrochemical CE. Error bars are calculated from the parallel tests.

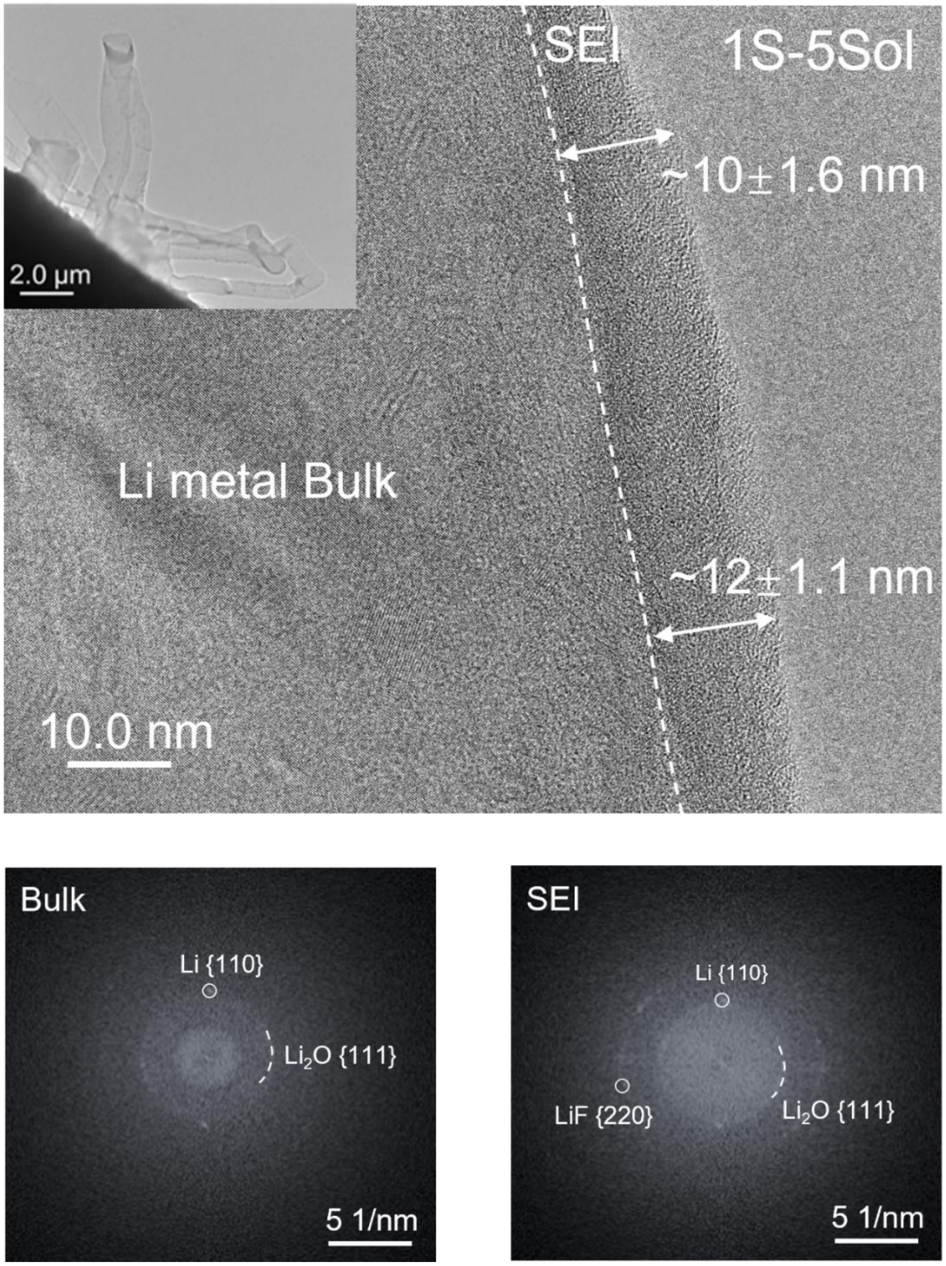

Supplementary Fig. 18. Microstructure of deposited Li metal and interfacial phase from cryo−transmission electron microscopy (cryo-TEM). Li was deposited in1S−5Sol electrolyte. The inset shows the morphology of Li-metal deposits at low magnification. The Selected-area electron-diffraction (SAED) patterns correspond to the SEI and Li metal bulk regions, respectively.

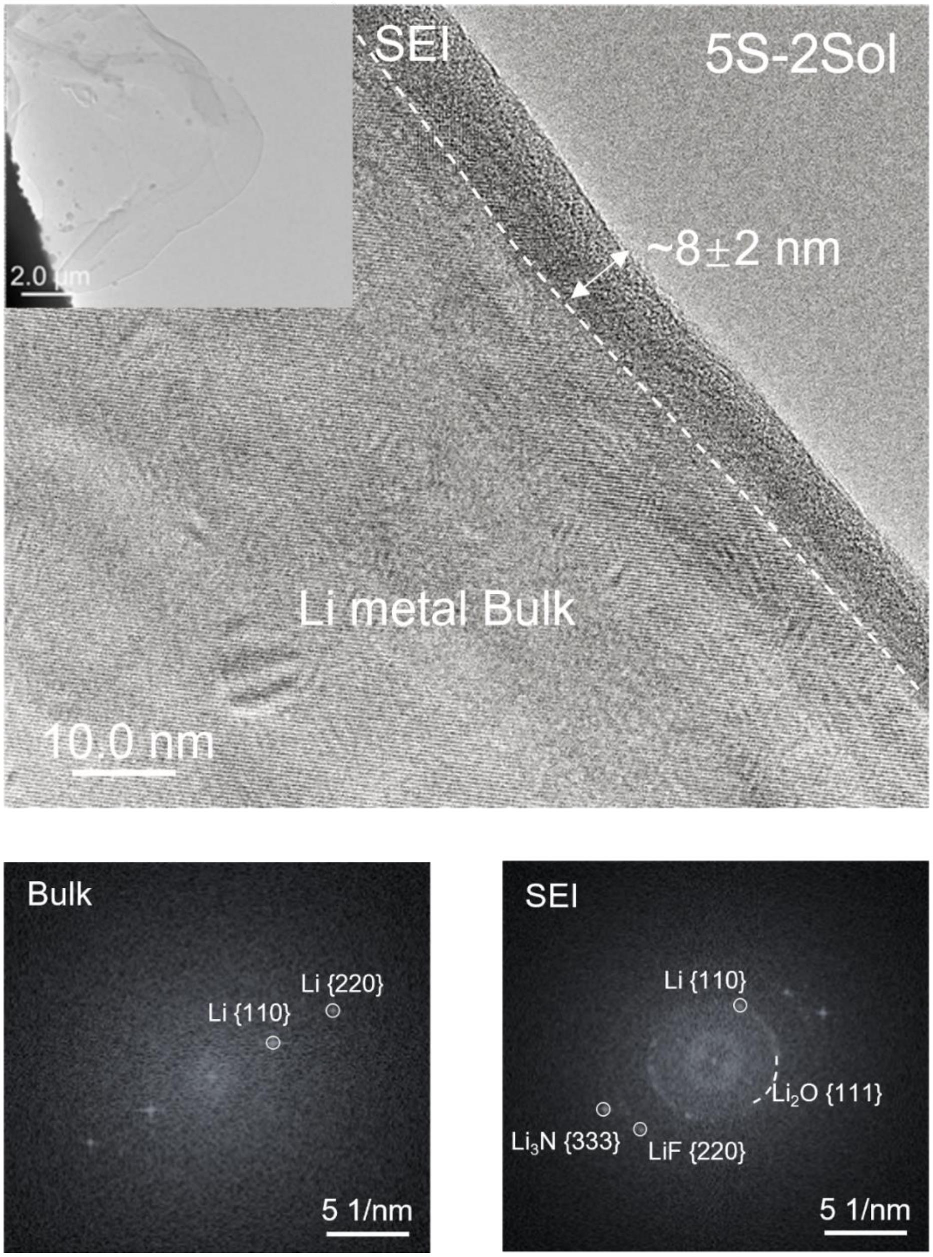

Supplementary Fig. 19. Microstructure of deposited Li metal and interfacial phase from cryo-TEM. Li was deposited in 5S−2Sol electrolyte. The inset shows the morphology of Li-metal deposits at low magnification. The SAED patterns correspond to the SEI and Li metal bulk regions, respectively.

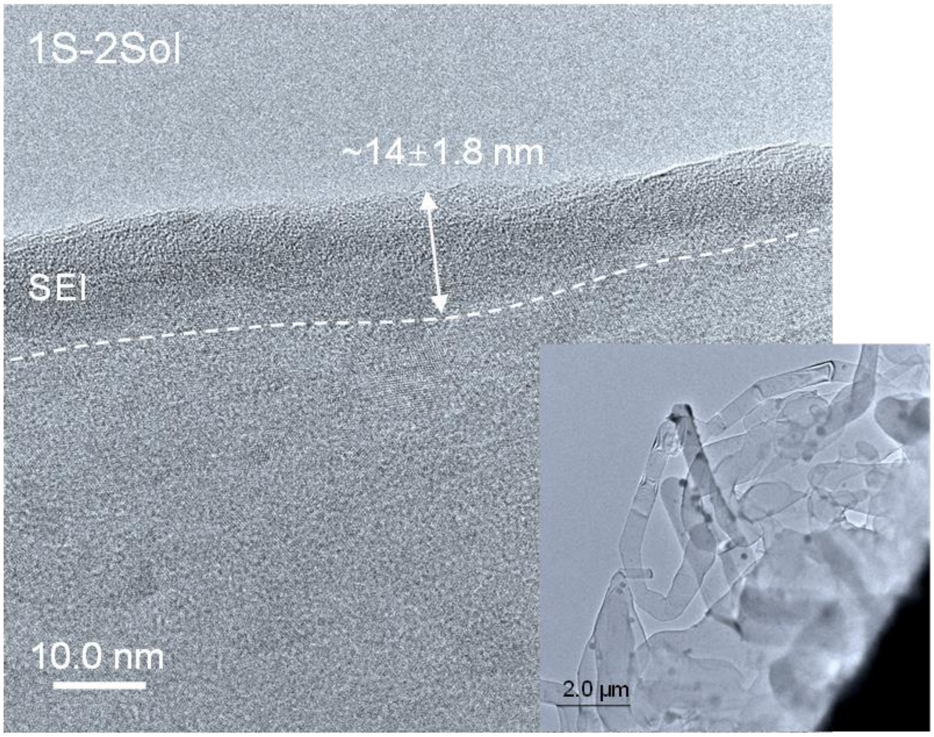

Supplementary Fig. 20. Microstructure of deposited Li metal and interfacial phase from cryo-TEM using 1S−2Sol electrolyte. The inset shows the morphology of Li-metal deposits at low magnification.

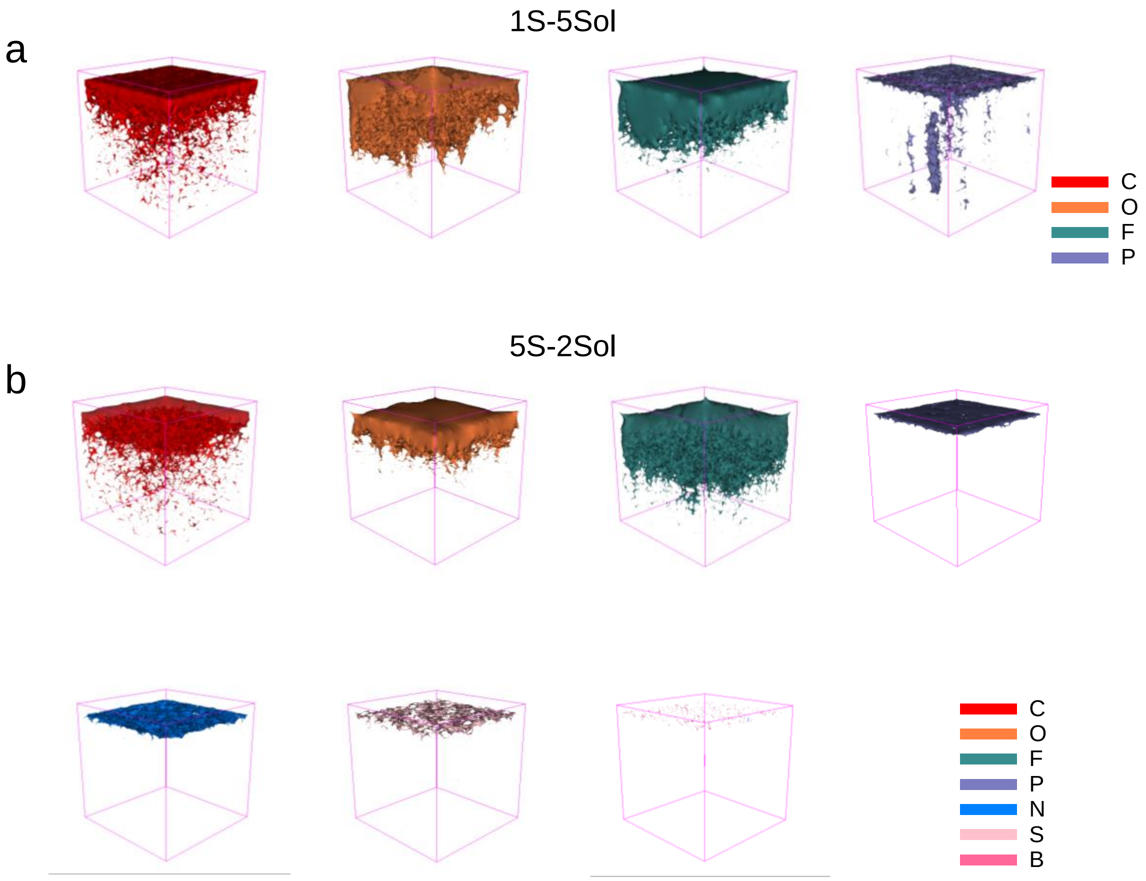

Supplementary Fig. 21. Time-of-flight secondary ion mass spectrometry (TOF-SIMS) analysis of the SEI on Li-metal deposits after 20 cycles at 0.5 mA cm^{-2} for 2 h in 1S−5Sol electrolyte and 5S−2Sol electrolyte solutions.

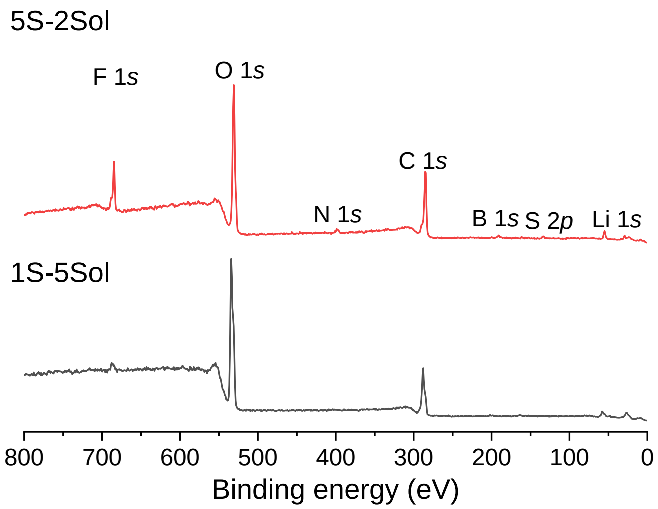

Supplementary Fig. 22. Surveys of X-ray photoelectron spectroscopy (XPS) data of deposited Li metal in different electrolyte solutions. Spectra were recorded immediately after 20 cycles from the surface of Cu foils.

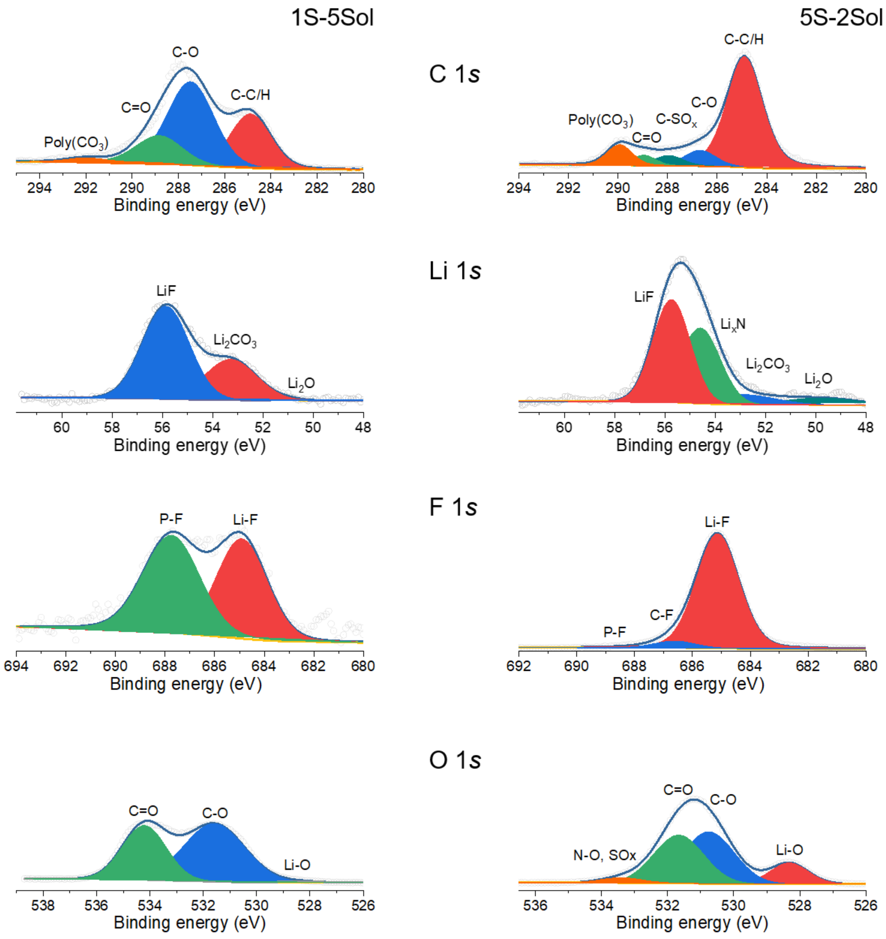

Supplementary Fig. 23. XPS data related to the surface of the Cu electrodes. The left panel relates to a treatment in the 1S−5Sol electrolyte, and the right panel relates to a treatment in the 5S−2Sol electrolyte.

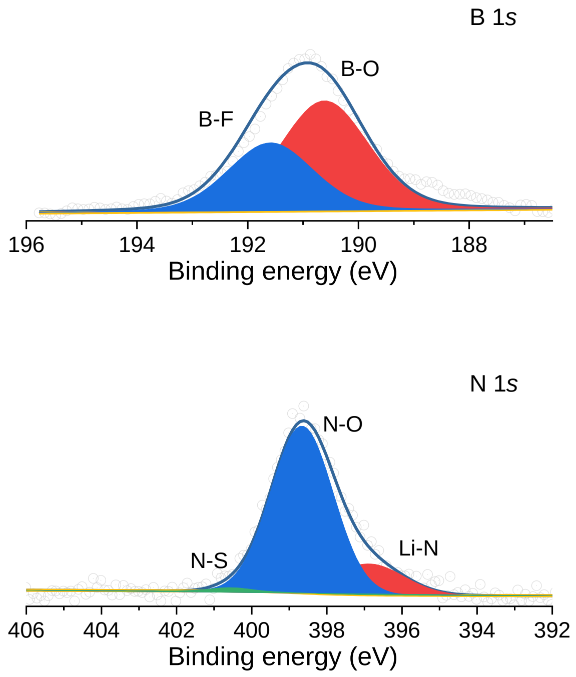

Supplementary Fig. 24. XPS data from the surface of the Cu electrode after being treated in the 5S−2Sol electrolyte.

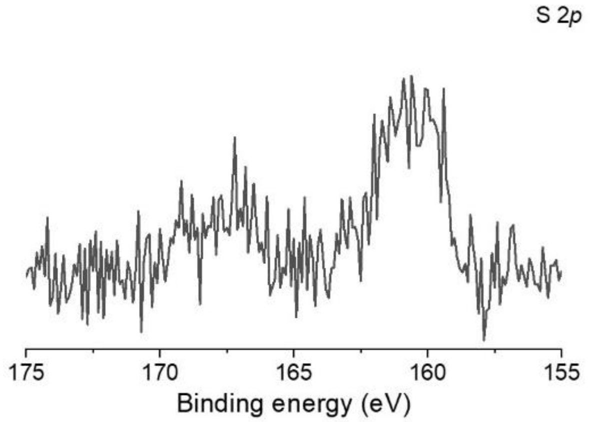

Supplementary Fig. 25. S 2*p* spectrum of the surface of the Cu electrode after being treated in the 5S−2Sol electrolyte.

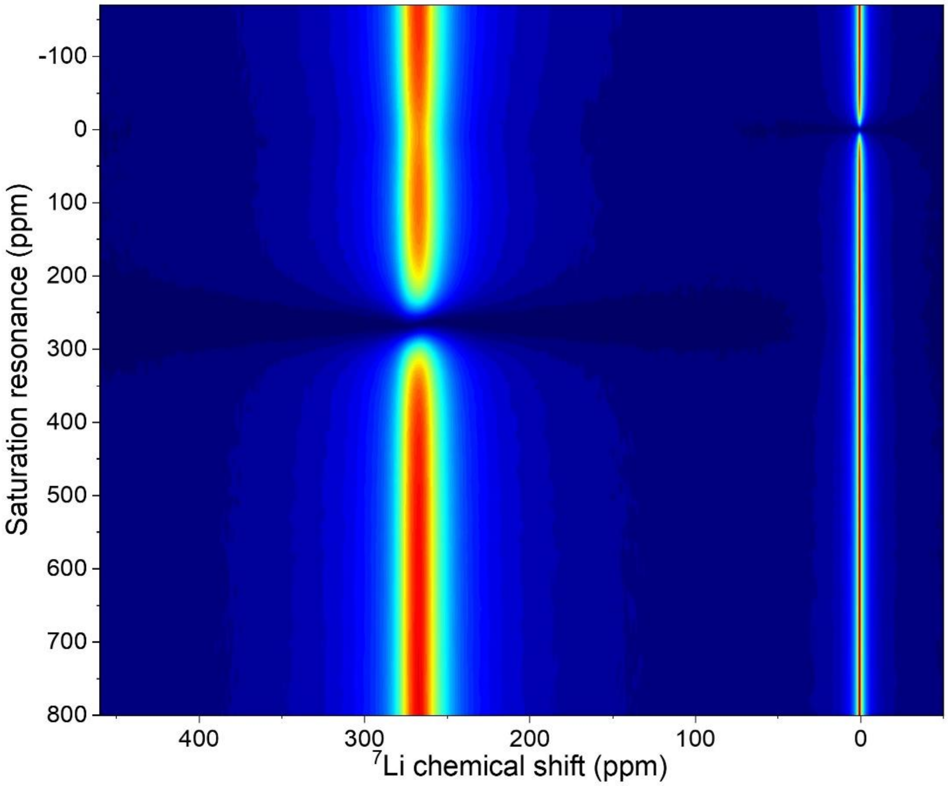

Supplementary Fig. 26. Z-spectra obtained from Li-metal deposits as a function of saturation frequencies in 5S−2Sol electrolyte. These spectra were acquired with a saturation pulse of 0.2 s at 3500 Hz as a function of saturation frequencies.

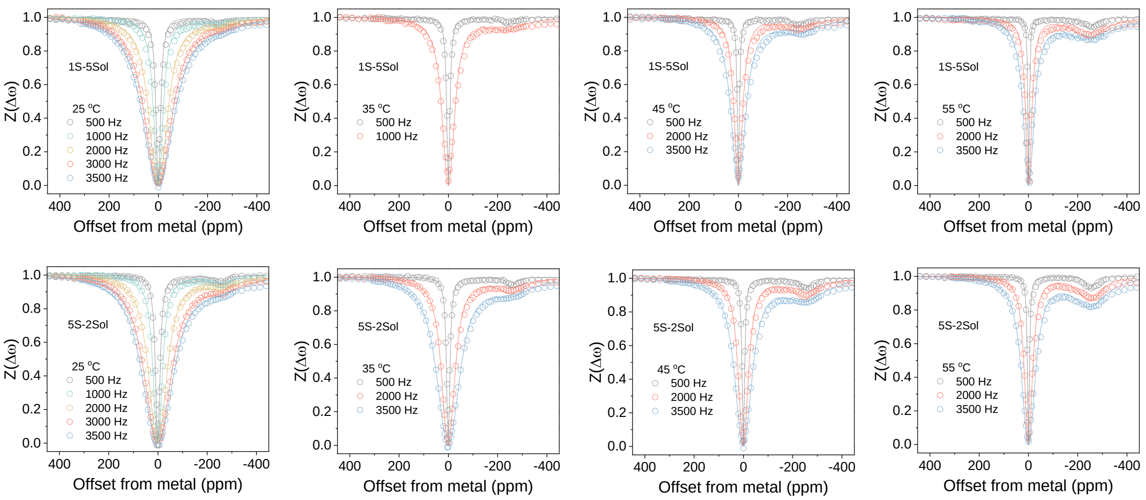

Supplementary Fig. 27. Z-spectra obtained from Li-metal deposits as a function of saturation frequencies for different electrolytes. Z-spectra obtained from Li deposits with a saturation time of 0.2 s at different temperatures with various saturation powers from 500 to 3500 Hz.

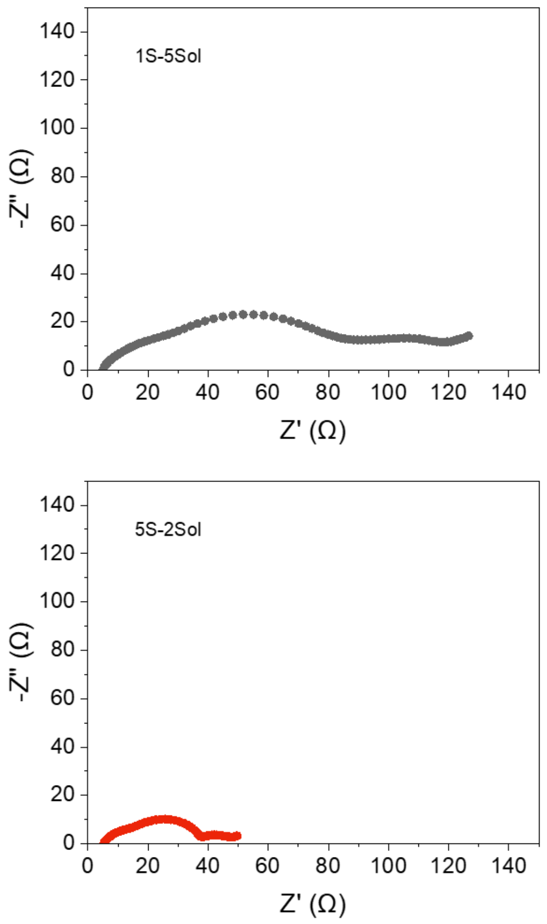

Supplementary Fig. 28. EIS of Li||Cu cells containing different electrolyte solutions (indicated in the charts). The Li||Cu cells were cycled at the continuous Li plating/stripping at 1.0 mA cm^{-2} for 1 h (1.0 mAh cm^{-2}) for 20 cycles.

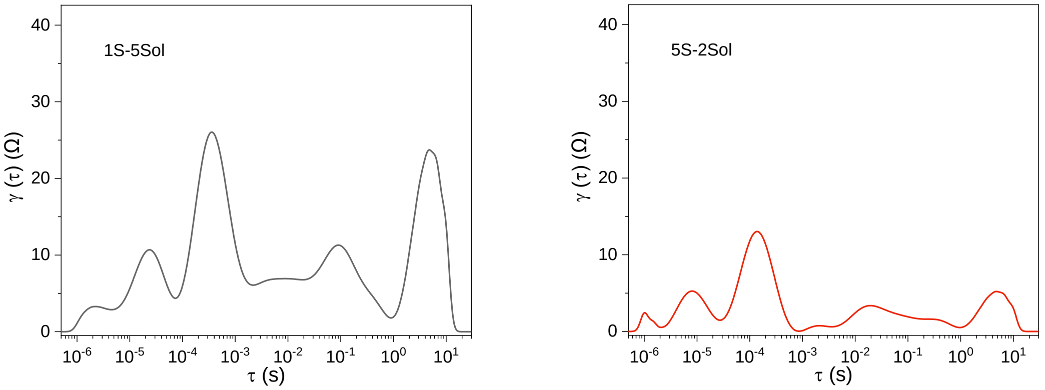

Supplementary Fig. 29. Distribution of relaxation times (DRT) analysis of the cells. The Li||Cu cells were first cycled at continuous Li plating/stripping at 1.0 mA cm^{-2} for 1 h (1.0 mAh cm^{-2}) for 20 cycles.

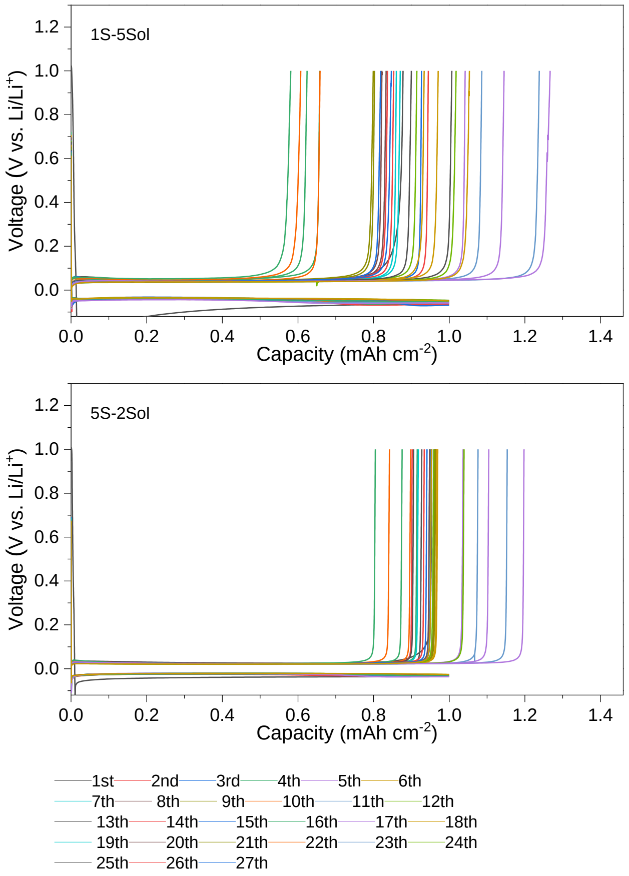

Supplementary Fig. 30. Intermittent charge-discharge curves of Li||Cu cells in 1S−5Sol and 5S−2Sol electrolytes.

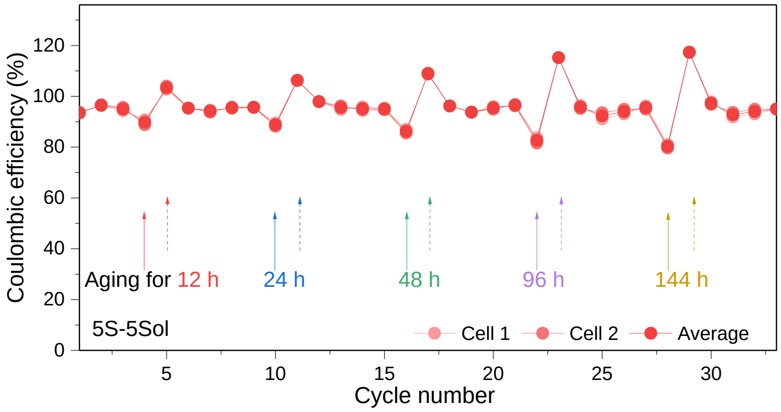

Supplementary Fig. 31. Intermittent CE of Li||Cu cells for different processes and various aging times in 5S−5Sol electrolyte solution. Firstly, Li||Cu cells underwent three cycles of continuous cycling at 1.0 mA cm^{-2} for 1 h to represent the normal cycling experiments. Subsequently, in the aging cycle (marked by solid arrow) Li metal was plated at 1.0 mA cm^{-2} for 1 h and aged for various periods before being stripped at 1.0 mA cm^{-2} to the cut-off voltage of 1.0 V. Following the aging cycle, Li-metal wash plated and stripped at the same current density, representing the recovery cycle (marked by dashed arrow).

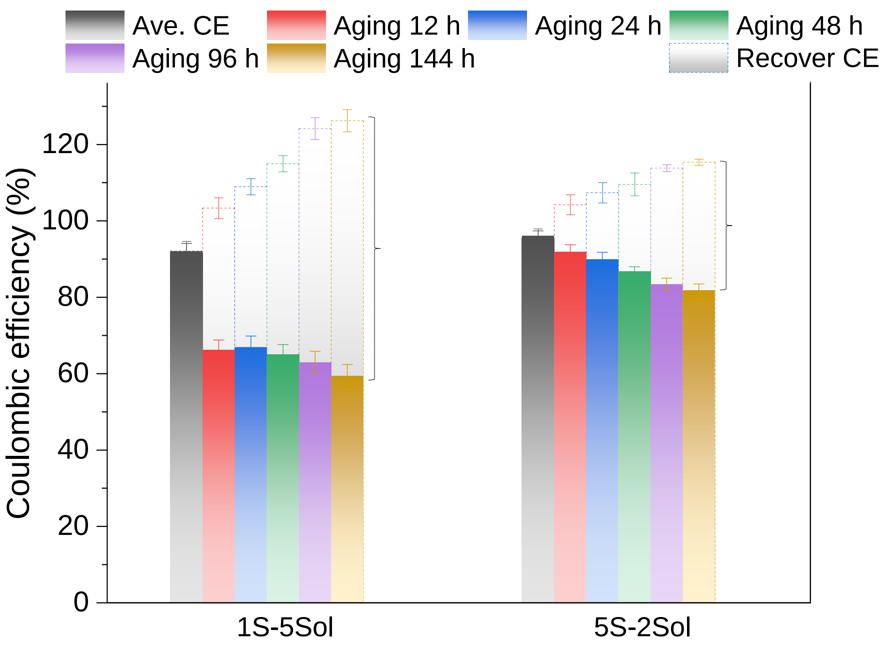

Supplementary Fig. 32. Intermittent CE of Li||Cu cells with the 2 electrolytes (indicated) for different processes and various aging times. Brackets show the CE difference between the recovery cycle and the aging cycle for various aging times.

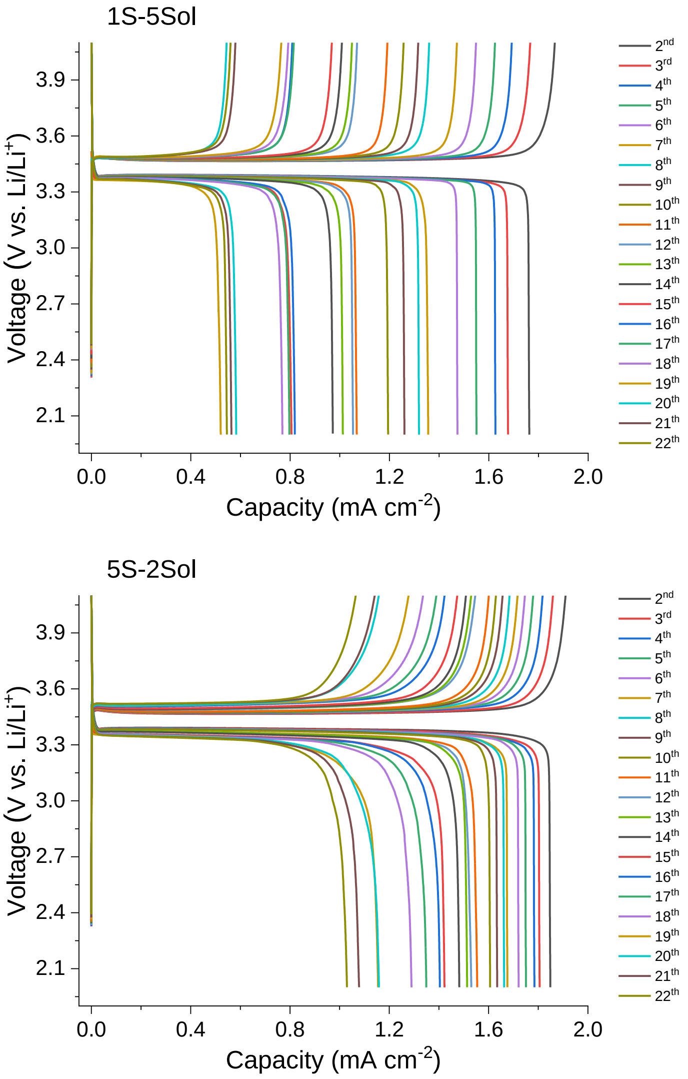

Supplementary Fig. 33. Intermittent charge-discharge curves of Cu||$LiFePO_4$ cells. The Cu||$LiFePO_4$ cells containing the two solutions (indicated) were first cycled in continuous charge-discharge processes at C/5 for two cycles then aged for different periods. They underwent after this stage three continuous cycles using the same current density.

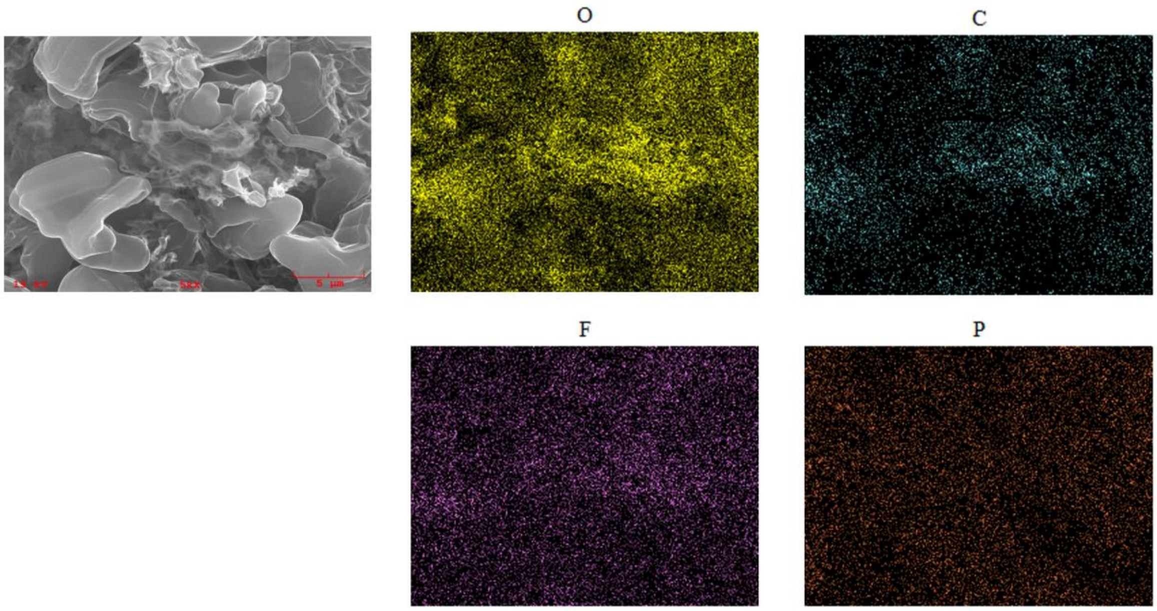

Supplementary Fig. 34. SEM images and energy dispersive spectroscopy (EDS) mappings of deposited Li after aging during 120 h in 1S−5Sol electrolyte solution. Cu||$LiFePO_4$ cells were cycled at 1.0 mA cm^{-2} for 1 h (1.0 mA h cm^{-2}) after twenty cycles and then Li was plated for 1 h.

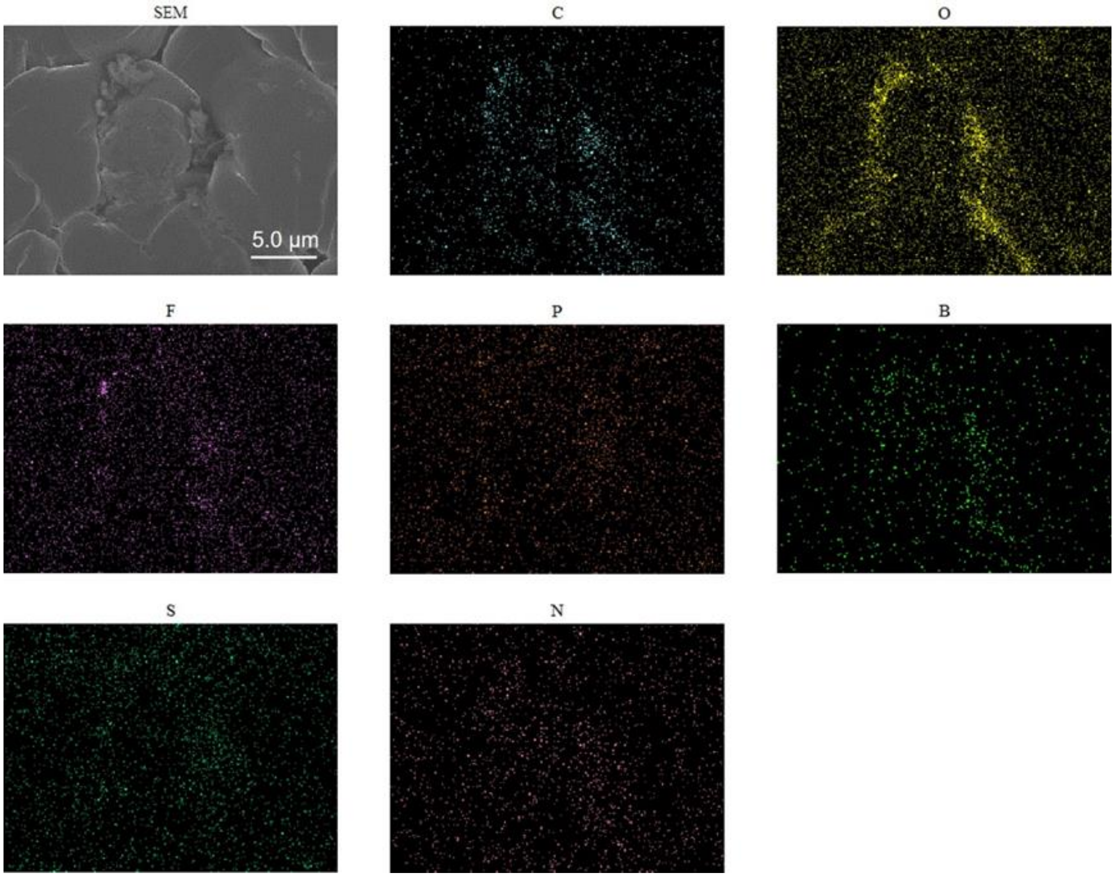

Supplementary Fig. 35. SEM images and EDS mappings of deposited Li after aging during 120 h in 5S−2Sol electrolyte solution. Cu||$LiFePO_4$ cells were cycled at 1.0 mA cm^{-2} for 1 h (1.0 mA h cm^{-2}) after twenty cycles and then Li was plated for 1 h.

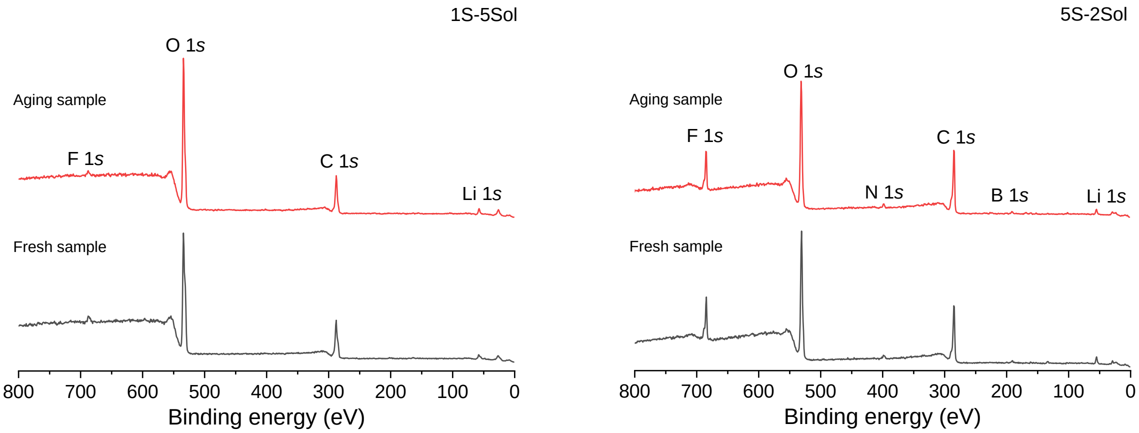

Supplementary Fig. 36. Surveys of XPS data measured from deposited Li metal in different electrolyte solutions (indicated). The fresh samples were recorded immediately after 20 cycles from the surface of Cu foils and the aging samples present the Li||Cu cells aged for 120 h after 20 cycles at 0.5 mA cm^{-2} for 1.0 mAh cm^{-2}.

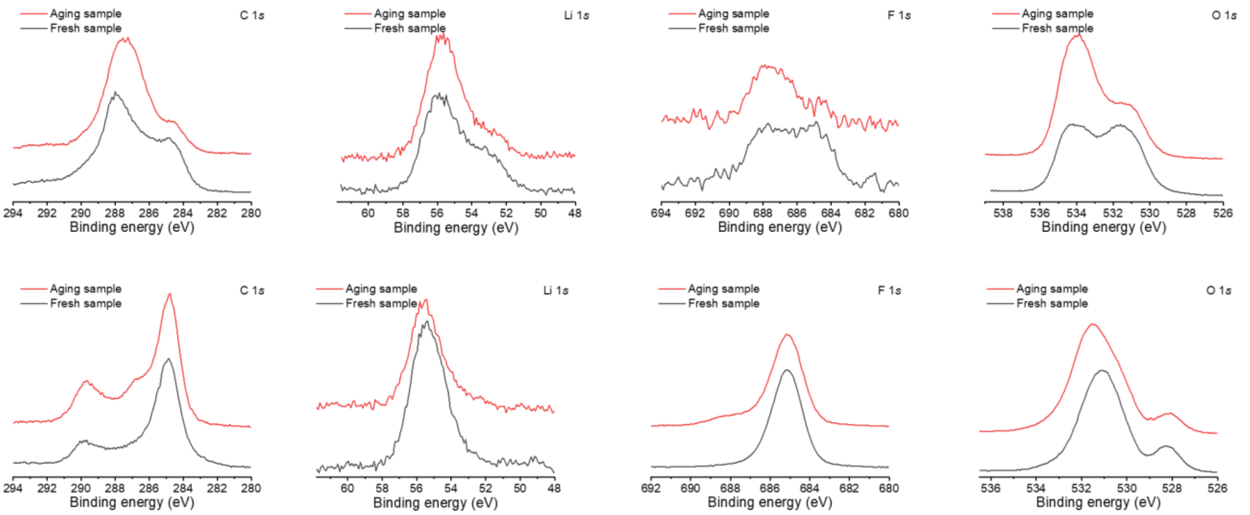

Supplementary Fig. 37. Survey XPS data of deposited Li metal in different electrolytes solutions (indicated). The spectra of the Li-metal deposits with and without resting period were collected. The top panel shows the spectra from the samples after being treated in the 1S−5Sol electrolyte, and the bottom panel shows the spectra from the samples that were treated in the 5S−2Sol electrolyte.

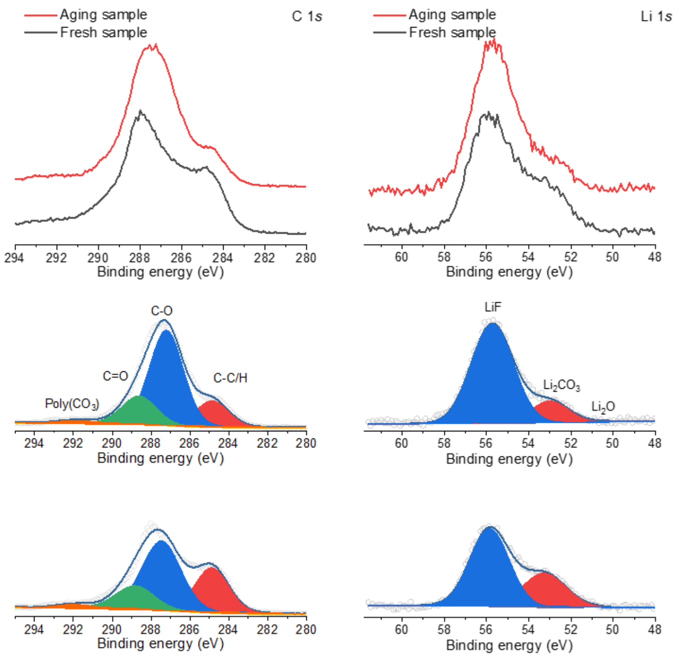

Supplementary Fig. 38. XPS data from the surface of the Cu electrodes treated in 1S−5Sol electrolyte. The panel in the middle is the peak deconvolution of the spectra of samples after aging, and the bottom panel shows the peak deconvolution of the spectra for the fresh samples.

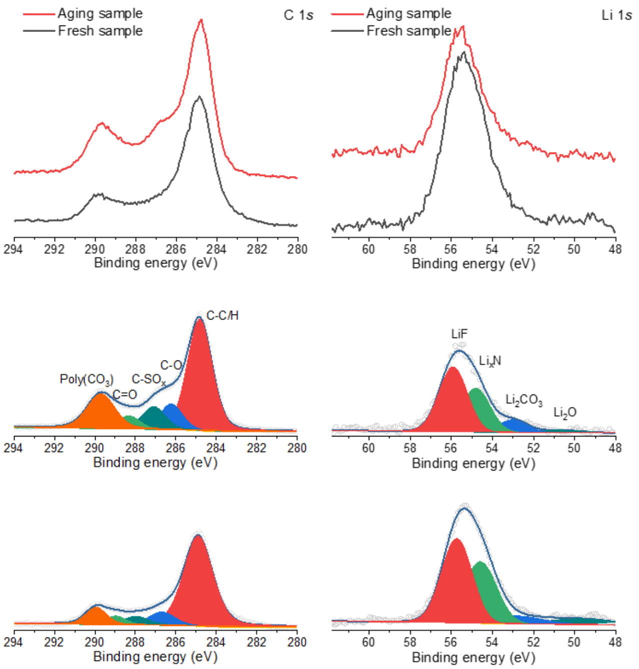

Supplementary Fig. 39. XPS data from the surface of the Cu electrodes in 5S−2Sol electrolyte solution. The panel in the middle is the peaks' deconvolution of the spectra of samples after aging, and the bottom panel is the peaks' deconvolution of the spectra of the fresh samples.

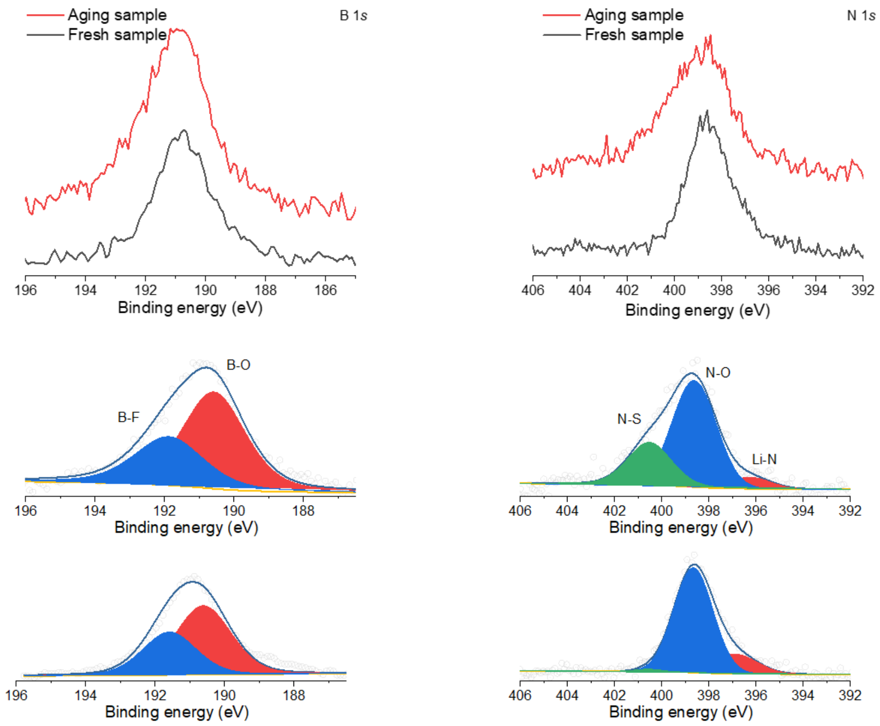

Supplementary Fig. 40. B 1*s* and N 1*s* XPS data from the surface of Cu electrodes treated in the 5S−2Sol electrolyte solution. The panel in the middle is the peaks' deconvolution of the spectra of samples after aging, and the bottom panel is the peaks' deconvolution of the spectra of the fresh samples.

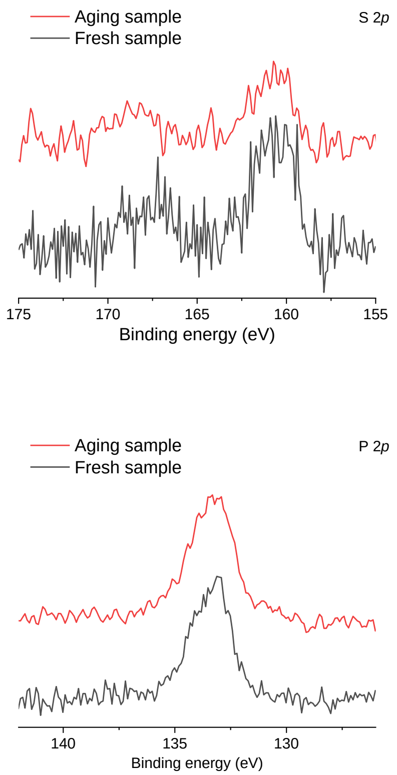

Supplementary Fig. 41. S 2*p* and P 2*p* spectra of Cu electrodes after treatment in 5S−2Sol electrolyte solution.

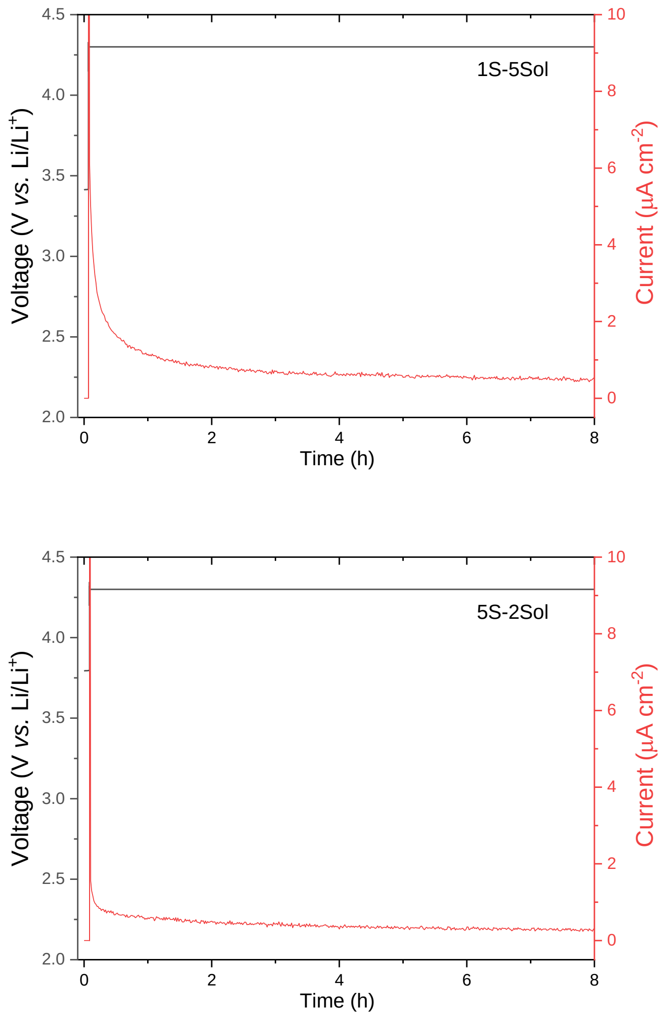

Supplementary Fig. 42. Potentiostatic charge profiles. Li||Al coin cells were used to study the corrosion current of Al foils (cathodes' current collectors) in different electrolyte solutions (indicated) at a polarization potential of 4.3 V vs. Li/Li^+ for 8 h, where both electrolyte solutions showed a stable anodic current under the same conditions.

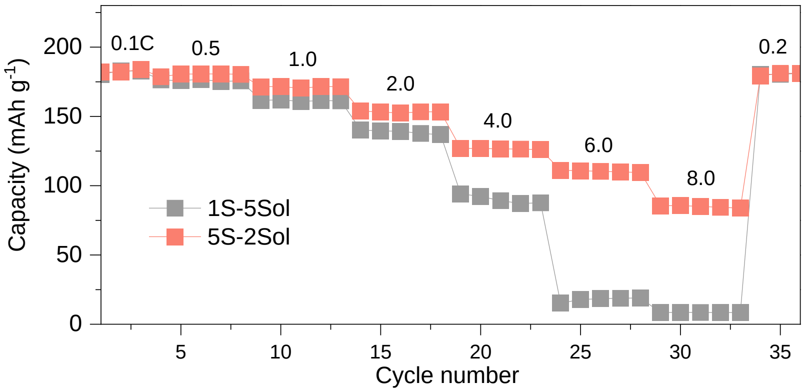

Supplementary Fig. 43. Rate performance of Li||NCM811 cells cycled between 2.8−4.3 V under various current densities with different electrolyte solutions (indicated). The discharge capacities are shown under various current densities in different electrolytes.

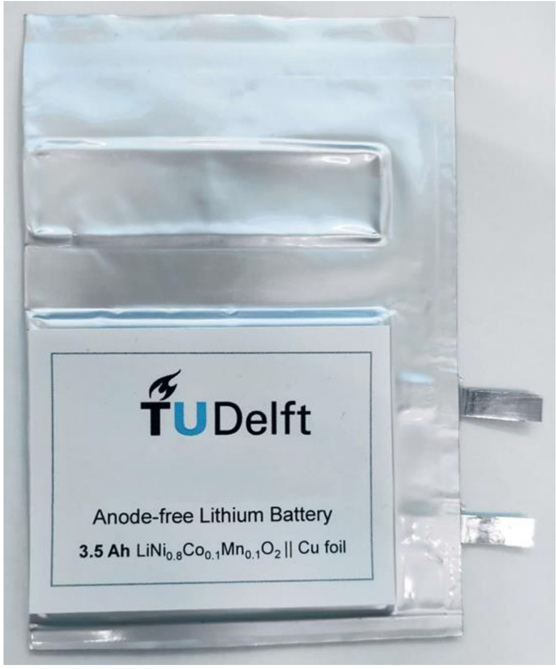

Supplementary Fig. 44. Anode-free NCM811||Cu pouch cell.

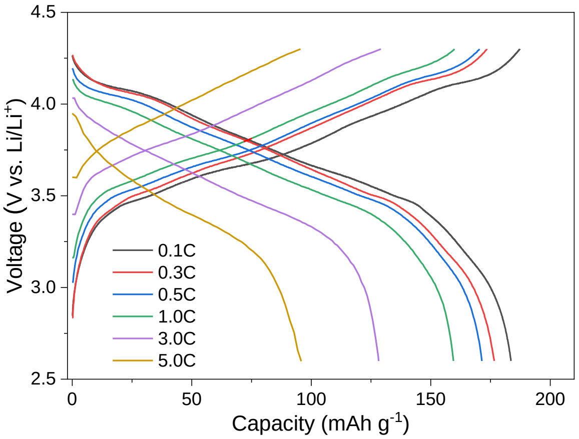

Supplementary Fig. 45. Galvanostatic charge/discharge curves of NCM811||Si/graphite cells containing the 5S−2Sol electrolyte solution in the voltage range of 2.6−4.3 V. The capacity ratio of the negative over the positive electrodes was in the range of 1.1~1.15.

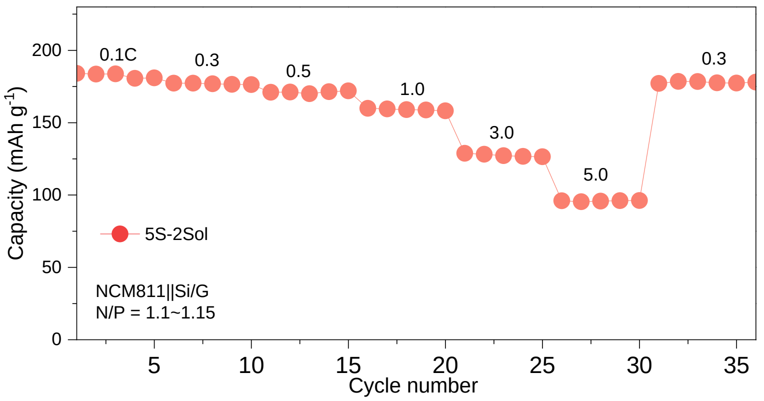

Supplementary Fig. 46. Rate performance of NCM811||Si/graphite cells containing the 5S−2Sol electrolyte solution cycled between 2.6−4.3 V under various current densities. The capacity ratio of the negative over the positive electrode was in the range of 1.1~1.15.

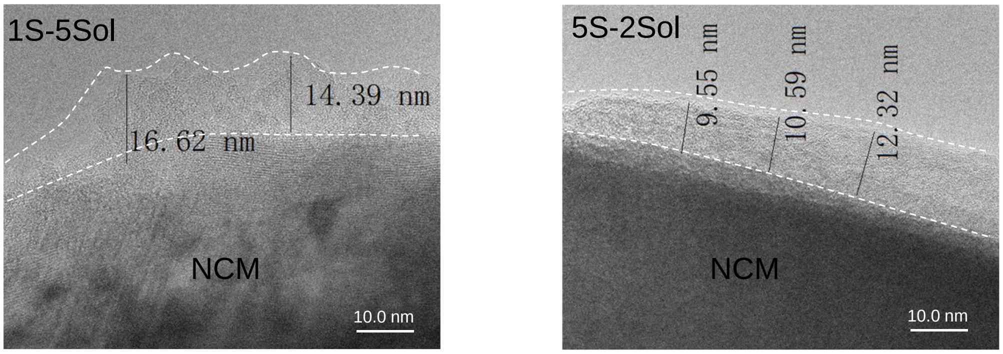

Supplementary Fig. 47. High-resolution scanning transmission electron microscopy (STEM) images of NCM811 cathode after cycling in different electrolyte solutions (indicated).

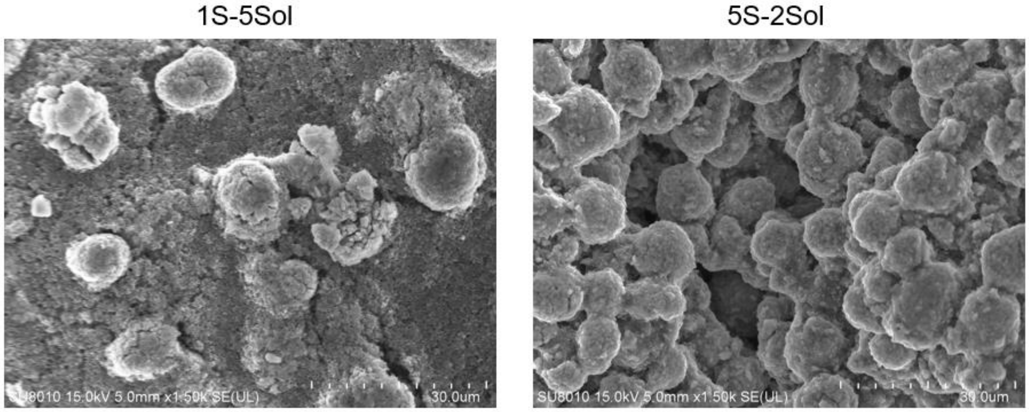

Supplementary Fig. 48. SEM images of NCM811 cathodes after 50 cycles in cells containing Li anodes and the electrolyte solutions indicated. It can be observed that the secondary particles are damaged during cycling in 1S−5Sol electrolyte.

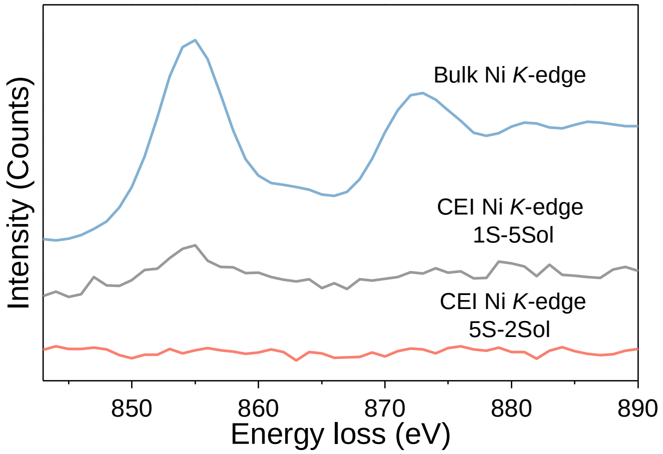

Supplementary Fig. 49. Electron energy-loss spectra (EELS) of Ni *K*-edge related to the surface films of a NCM811 cathode after cycling in the two indicated electrolyte solutions. The bulk spectrum is provided as well.

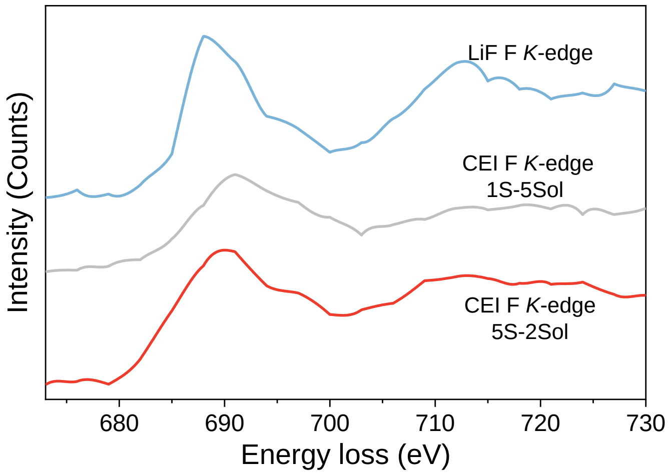

Supplementary Fig. 50. EELS of F *K*-edge related to the surface films on the NCM811 cathodes after cycling the two indicated electrolyte solutions. LiF spectrum is provided as a reference.

Table S1. Fitting parameters and boundaries.

**Parameter**	**Starting value**	**Lower boundary**	**Upper boundary**
$\Delta\omega_{Li}$ [ppm]	0	-5	5
$R_{2, Li}$ [s^{-1}]	1000	0.5	2×10^4
$\Delta\omega_{SEI}$ [ppm]	-265	-215	315
K_{SEI-Li} [Hz]	1000	1	1×10^6
f_S	0.01	0	2
$R_{2, SEI}$ [s^{-1}]	50	0	7×10^4

Table S2. Relaxation parameters from experiments and fitting two-pool BMC solution.

Electrolyte	T (°C)	$R_{1,\ Metal-E}$ (Hz)	$R_{2,\ Metal-E}$ (Hz)	$K_{SEI-Metal}$ (Hz)	$R_{2,\ Metal-F}$ (Hz)	$R_{2,\ SEI-F}$ (kHz)	GOF(R^2)
1S−5Sol	25	7.0	1360	58±7	936±25	30±11	0.76
	35	7.5	780	87±19	492±31	33±15	0.84
	45	7.8	512	129±15	240±12	39±22	0.78
	55	8.2	366	183±21	125±9	41±25	0.73
5S−2Sol	25	7.2	1200	96±8	984±17	28±10	0.86
	35	7.6	820	120±8	511±19	30±14	0.84
	45	8.1	504	168±10	298±12	38±14	0.83
	55	8.4	328	216±13	191±8	40±13	0.83

Table S3. Parameters of anode-free pouch cells.

Cell component	Specification	Parameters
Cathode (NCM with Al current collector)	Active material ratio	93
	Size (cm×cm)	7×6
	Number	12
	Weight (g)	17.216
Collector (Cu)	Thickness (μm)	6
	Size (cm×cm)	7.1×6.1
	Number	13
	Weight (g)	3.16
Electrolyte	Electrolyte/Capacity (g $(Ah)^{-1}$)	2.0
	Weight (g)	7.0
Separator	Weight (g)	0.825
Package and tabs	Weight (g)	3
Full cell	N/P	0
	Discharge capacity (Ah)	3.5
	Discharge energy (Wh)	13.475
	Total weight (g)	31.55
	Specific energy (Wh kg^{-1})	427.1

Table S4. Comparison of anode-free Li batteries performance containing different electrolytes.

Electrolyte	Cathode	Anode	Cycles	Capacity retention	Ref.
2 M $LiPF_6$ EC/DEC/FEC (1:1:2)	NCM111 1.6 mAh cm^{-2}	Cu	50	40%	[22]
1 M LiTFSI+2 M LiFSI+0.6 M $LiNO_3$ DME/DOL (1:1)	$LiFePO_4$ 5 mg cm^{-2}	Cu	100	40%	[23]
4 M LiFSI DME	$LiFePO_4$ 1.7 mAh cm^{-2}	Cu	50	60%	[24]
1 M LiFSI DME/HFE (1:2)	NCM811 3.0 mAh cm^{-2}	Cu	50	74.7%	[25]
0.6 M LiDFOB + 0.6 M $LiBF_4$ FEC/DEC (1:2)	NCM523 16 mg cm^{-2}	Cu	80	80%	[26]
1 M $LiPF_6$ FEC/TTE/EMC (3:5:2)	NCM111 12 mg cm^{-2}	Cu	80	40%	[27]
1.5 M LiFSI DME/TTE (1.2:3 by molar)	NMC622 4.0 mAh cm^{-2}	Cu	100	80%	[28]
1.8 M LiDFOB + 0.4 M $LiBF_4$ FEC/DEC (1:2)	NCM523 16 mg cm^{-2}	Cu	90	80%	[29]
7 M LiFSI FEC	$LiNi_{0.5}Mn_{1.5}O_2$ 1.83 mAh cm^{-2}	Cu	50	54%	[30]
1 M LiFSI FDMB	NCM811 4.2 mAh cm^{-2}	Cu	100	80%	[31]
1 M LiBF4 + 1 M LiDFOB FEP/FEC (1:2)	NCM811 4.64 mAh cm^{-2}	Cu	100	80%	[32]
2 M LiFSI+2 M $LiNO_3$ DME	NCM622 2.0 mAh cm^{-2}	Cu	100	47.3%	[33]
5S−2Sol	NCM811 3.5 mAh cm^{-2}	Cu	100	82%	This work

Table S5. Comparison of prices of Li salts used in the reported liquid electrolytes for Li-metal batteries.

Salt	Chemical name	Product Number	Size (g)	Price ($)	Unit price, ($/kg)
$LiPF_6$	Lithium Hexafluorophosphate	L0146	100	261.00	2610
$LiClO_4$	Lithium Perchlorate	L0379	500	240.00	480
LiFSI	Lithium Bis(fluorosulfonyl)imide	L0281	25	357.00	14280
LiTFSI	Lithium Bis(trifluoromethanesulfonyl)imide	B2542	250	495.00	1980
LiTFO	Lithium Trifluoromethanesulfonate	T1548	25	68.00	2720
LiDFOB*	Lithium difluoro(oxalato)borate	774138	25	335.00	13400
LiBOB	Lithium Bis(oxalate)borate	L0367	25	205.00	8200
$LiBF_4$	Lithium Tetrafluoroborate	L0133	25	142.00	5680
$LiPF_2O_2$	Lithium Phosphorodifluoridate	L0375	25	247.00	9880
$LiNO_3$*	Lithium nitrate	227986	1000	227.00	227

All prices listed here are based on TCI (Tokyo Chemical Industry) pricing data unless noted specifically.

The prices of electrolyte components are based on production trends and may fluctuate in response to market changes.

*Price data are from MilliporeSigma.

Table S6. Comparison of prices of solvents used for the reported liquid electrolytes in Li-metal batteries.

Solvent	Chemical name	Product Number	Size (ml)	Price ($)	Unit price, ($/L)
EC	Ethylene carbonate	E0076	500	29.00	58
DMC	Dimethyl carbonate	C0053	500	43.00	86
DEC	Diethyl carbonate	C0041	500	40.00	80
EMC	Ethyl methyl carbonate	C1342	25	113.00	4520
VC	Vinylene carbonate	V0015	25	237.00	9480
PC	Propylene carbonate	P0525	500	43.00	86
FEC	fluoroethylene carbonate	F0731	25	147.00	5880
DME	Dimethoxyethane	D0634	500	57.00	114
DEGDME	Diethylene glycol dimethyl ether	B0498	500	49.00	98
DOL	1,3−Dioxolane	D5539	500	47.00	94
TMP	Trimethyl phosphate	P0271	500	53.00	106
TEP	Triethyl phosphate	P0270	500	48.00	96
DMSO	Dimethyl sulfoxide	D5293	500	66.00	132
THF	Tetrahydrofuran	T0104	500	26.00	52
FEMC	Methyl 2,2,2−trifluoroethyl carbonate	M3376	25	454.00	18160
FEP	Methyl 3,3,3−Trifluoropropionate	M2783	5	272.00	54400
HFE	1,1,2,2−tetrafluoroethyl−2,2,2−trifluoroethyl ether	T3057	25	66.00	2640
TTE	Tetrafluoroethyl−2,2,3,3−Tetrafluoropropyl Ether	T3069	25	56.00	2240
BTFE	Bis(2,2,2−trifluoroethyl) ether	B1293	5	140.00	28000
TFEO*	Tris(2,2,2−trilfuoroethyl) orthoformate	ATE517251825	1	353.60	353600

All prices listed here are from TCI unless indicated specifically otherwise.

The prices of electrolyte components are based on production trends and may fluctuate in response to market changes.

* Taken from MilliporeSigma.

Table S7. Cost analysis of various electrolytes.

Electrolyte	Salt cost ($/L)	Solvent cost ($/L)	Total cost ($/L)	Ref.
2 M $LiPF_6$ EC/DEC/FEC (1:1:2)	792.94	2974.50	3767.44	[22]
1 M LiTFSI+2 M LiFSI+0.6 M $LiNO_3$ DME/DOL (1:1)	5920.52	104.00	6024.52	[23]
4 M LiFSI DME	10685.44	114.00	10799.44	[24]
1 M LiFSI DME/HFE(1:2)	2671.36	1798.00	4469.36	[25]
0.6 M LiDFOB + 0.6 M $LiBF_4$ FEC/DEC (1:2)	1475.40	2013.33	3488.73	[26]
1 M $LiPF_6$ FEC/TTE/EMC (3:5:2)	396.47	3788.00	4184.47	[27]
1.5 M LiFSI DME/TTE (1:3.65)	4007.04	1782.80	5789.84	[28]
1.8 M LiDFOB + 0.4 M $LiBF_4$ FEC/DEC (1:2)	3680.72	2013.33	5694.06	[29]
7 M LiFSI FEC	18699.52	5880.00	24579.52	[30]
1 M LiFSI FDMB*	2671.36	~4000	6671.36	[31]
1 M $LiBF_4$ + 1 M LiDFOB FEP/FEC (1:2)	2459.00	22053.33	24512.33	[32]
2 M LiFSI+2 M $LiNO_3$ DME	5374.02	114.00	5488.02	[33]
5S−2Sol	1253.15	359.55	1612.70	This work

All prices listed here are from TCI unless indicated specifically otherwise.

The prices of electrolyte components are based on production trends and may fluctuate in response to market changes.

*The price of solvents is calculated based on an average level of the fluorinated solvent.

References

1. Wang, Q.; Zhao, C.; Wang, J.; Yao, Z.; Wang, S.; Kumar, S. G. H.; Ganapathy, S.; Eustace, S.; Bai, X.; Li, B.; Wagemaker, M., High entropy liquid electrolytes for lithium batteries. *Nature Communications* **2023,** *14* (1), 440.
2. Amanchukwu, C. V.; Yu, Z.; Kong, X.; Qin, J.; Cui, Y.; Bao, Z., A New Class of Ionically Conducting Fluorinated Ether Electrolytes with High Electrochemical Stability. *Journal of the American Chemical Society* **2020,** *142* (16), 7393-7403.
3. Rosenfeld, Y., Relation between the transport coefficients and the internal entropy of simple systems. *Physical Review A* **1977,** *15* (6), 2545-2549.
4. Dyre, J. C., Perspective: Excess-entropy scaling. *The Journal of Chemical Physics* **2018,** *149* (21), 210901.
5. Wang, Q.; Yao, Z.; Zhao, C.; Verhallen, T.; Tabor, D. P.; Liu, M.; Ooms, F.; Kang, F.; Aspuru-Guzik, A.; Hu, Y.-S.; Wagemaker, M.; Li, B., Interface chemistry of an amide electrolyte for highly reversible lithium metal batteries. *Nature Communications* **2020,** *11* (1), 4188.
6. Chang, H. J.; Ilott, A. J.; Trease, N. M.; Mohammadi, M.; Jerschow, A.; Grey, C. P., Correlating Microstructural Lithium Metal Growth with Electrolyte Salt Depletion in Lithium Batteries Using 7Li MRI. *Journal of the American Chemical Society* **2015,** *137* (48), 15209-15216.
7. Bhattacharyya, R.; Key, B.; Chen, H.; Best, A. S.; Hollenkamp, A. F.; Grey, C. P., In situ NMR observation of the formation of metallic lithium microstructures in lithium batteries. *Nature Materials* **2010,** *9* (6), 504-510.
8. Gunnarsdóttir, A. B.; Amanchukwu, C. V.; Menkin, S.; Grey, C. P., Noninvasive In Situ NMR Study of "Dead Lithium" Formation and Lithium Corrosion in Full-Cell Lithium Metal Batteries. *Journal of the American Chemical Society* **2020,** *142* (49), 20814-20827.
9. Zhan, C.; Wu, T.; Lu, J.; Amine, K., Dissolution, migration, and deposition of transition metal ions in Li-ion batteries exemplified by Mn-based cathodes – a critical review. *Energy & Environmental Science* **2018,** *11* (2), 243-257.
10. Bryant, R. G., The dynamics of water-protein interactions. *Annu Rev Biophys Biomol Struct* **1996,** *25* (1), 29-53.
11. van Zijl, P. C. M.; Yadav, N. N., Chemical exchange saturation transfer (CEST): What is in a name and what isn't? *Magnetic Resonance in Medicine* **2011,** *65* (4), 927-948.
12. Guivel-Scharen, V.; Sinnwell, T.; Wolff, S. D.; Balaban, R. S., Detection of Proton Chemical Exchange between Metabolites and Water in Biological Tissues. *Journal of Magnetic Resonance* **1998,** *133* (1), 36-45.
13. Zaiss, M.; Angelovski, G.; Demetriou, E.; McMahon, M. T.; Golay, X.; Scheffler, K., QUESP and

QUEST revisited – fast and accurate quantitative CEST experiments. *Magnetic Resonance in Medicine* **2018,** *79* (3), 1708-1721.

14. Zaiss, M.; Schnurr, M.; Bachert, P., Analytical solution for the depolarization of hyperpolarized nuclei by chemical exchange saturation transfer between free and encapsulated xenon (HyperCEST). *The Journal of Chemical Physics* **2012,** *136* (14), 144106.

15. Goerke, S.; Zaiss, M.; Bachert, P., Characterization of creatine guanidinium proton exchange by water-exchange (WEX) spectroscopy for absolute-pH CEST imaging in vitro. *NMR in Biomedicine* **2014,** *27* (5), 507-518.

16. McConnell, H. M., Reaction Rates by Nuclear Magnetic Resonance. *The Journal of Chemical Physics* **1958,** *28* (3), 430-431.

17. Woessner, D. E.; Zhang, S.; Merritt, M. E.; Sherry, A. D., Numerical solution of the Bloch equations provides insights into the optimum design of PARACEST agents for MRI. *Magnetic Resonance in Medicine* **2005,** *53* (4), 790-799.

18. Zaiss, M.; Bachert, P., Exchange-dependent relaxation in the rotating frame for slow and intermediate exchange – modeling off-resonant spin-lock and chemical exchange saturation transfer. *NMR in Biomedicine* **2013,** *26* (5), 507-518.

19. Columbus, D.; Arunachalam, V.; Glang, F.; Avram, L.; Haber, S.; Zohar, A.; Zaiss, M.; Leskes, M., Direct Detection of Lithium Exchange across the Solid Electrolyte Interphase by 7Li Chemical Exchange Saturation Transfer. *Journal of the American Chemical Society* **2022,** *144* (22), 9836-9844.

20. Sang Cheol Kim, J. W., Rong Xu, Pu Zhang, Yuelang Chen, Zhuojun Huang,Yufei Yang, Zhiao Yu, Solomon Oyakhire, Wenbo Zhang, Mun Sek Kim, David Boyle, Philaphon Sayavong, Jian Qin ,Zhenan Bao, Yi Cui, High Entropy Electrolytes for Practical Lithium Metal Batteries. *Chemrxiv* **2022**.

21. Wan, T. H.; Saccoccio, M.; Chen, C.; Ciucci, F., Influence of the Discretization Methods on the Distribution of Relaxation Times Deconvolution: Implementing Radial Basis Functions with DRTtools. *Electrochimica Acta* **2015,** *184*, 483-499.

22. Hagos, T. T.; Thirumalraj, B.; Huang, C.-J.; Abrha, L. H.; Hagos, T. M.; Berhe, G. B.; Bezabh, H. K.; Cherng, J.; Chiu, S.-F.; Su, W.-N.; Hwang, B.-J., Locally Concentrated LiPF6 in a Carbonate-Based Electrolyte with Fluoroethylene Carbonate as a Diluent for Anode-Free Lithium Metal Batteries. *ACS Applied Materials & Interfaces* **2019,** *11* (10), 9955-9963.

23. Qiu, F.; Li, X.; Deng, H.; Wang, D.; Mu, X.; He, P.; Zhou, H., A Concentrated Ternary-Salts Electrolyte for High Reversible Li Metal Battery with Slight Excess Li. *Advanced Energy Materials* **2019,** *9* (6), 1803372.

24. Qian, J.; Adams, B. D.; Zheng, J.; Xu, W.; Henderson, W. A.; Wang, J.; Bowden, M. E.; Xu, S.; Hu, J.; Zhang, J.-G., Anode-Free Rechargeable Lithium Metal Batteries. *Advanced Functional Materials* **2016,** *26*

(39), 7094-7102.

25. Zhang, J.; Zhang, H.; Deng, L.; Yang, Y.; Tan, L.; Niu, X.; Chen, Y.; Zeng, L.; Fan, X.; Zhu, Y., An additive-enabled ether-based electrolyte to realize stable cycling of high-voltage anode-free lithium metal batteries. *Energy Storage Materials* **2023,** *54*, 450-460.

26. Weber, R.; Genovese, M.; Louli, A. J.; Hames, S.; Martin, C.; Hill, I. G.; Dahn, J. R., Long cycle life and dendrite-free lithium morphology in anode-free lithium pouch cells enabled by a dual-salt liquid electrolyte. *Nature Energy* **2019,** *4* (8), 683-689.

27. Hagos, T. M.; Hagos, T. T.; Bezabh, H. K.; Berhe, G. B.; Abrha, L. H.; Chiu, S.-F.; Huang, C.-J.; Su, W.-N.; Dai, H.; Hwang, B. J., Resolving the Phase Instability of a Fluorinated Ether, Carbonate-Based Electrolyte for the Safe Operation of an Anode-Free Lithium Metal Battery. *ACS Applied Energy Materials* **2020,** *3* (11), 10722-10733.

28. Niu, C.; Liu, D.; Lochala, J. A.; Anderson, C. S.; Cao, X.; Gross, M. E.; Xu, W.; Zhang, J.-G.; Whittingham, M. S.; Xiao, J.; Liu, J., Balancing interfacial reactions to achieve long cycle life in high-energy lithium metal batteries. *Nature Energy* **2021,** *6* (7), 723-732.

29. Genovese, M.; Louli, A. J.; Weber, R.; Martin, C.; Taskovic, T.; Dahn, J. R., Hot Formation for Improved Low Temperature Cycling of Anode-Free Lithium Metal Batteries. *Journal of The Electrochemical Society* **2019,** *166* (14), A3342.

30. Suo, L.; Xue, W.; Gobet, M.; Greenbaum, S. G.; Wang, C.; Chen, Y.; Yang, W.; Li, Y.; Li, J., Fluorine-donating electrolytes enable highly reversible 5-V-class Li metal batteries. *Proceedings of the National Academy of Sciences* **2018,** *115* (6), 1156-1161.

31. Yu, Z.; Wang, H.; Kong, X.; Huang, W.; Tsao, Y.; Mackanic, D. G.; Wang, K.; Wang, X.; Huang, W.; Choudhury, S.; Zheng, Y.; Amanchukwu, C. V.; Hung, S. T.; Ma, Y.; Lomeli, E. G.; Qin, J.; Cui, Y.; Bao, Z., Molecular design for electrolyte solvents enabling energy-dense and long-cycling lithium metal batteries. *Nature Energy* **2020,** *5* (7), 526-533.

32. Mao, M.; Ji, X.; Wang, Q.; Lin, Z.; Li, M.; Liu, T.; Wang, C.; Hu, Y.-S.; Li, H.; Huang, X.; Chen, L.; Suo, L., Anion-enrichment interface enables high-voltage anode-free lithium metal batteries. *Nature Communications* **2023,** *14* (1), 1082.

33. Kang, D. W.; Moon, J.; Choi, H.-Y.; Shin, H.-C.; Kim, B. G., Stable cycling and uniform lithium deposition in anode-free lithium-metal batteries enabled by a high-concentration dual-salt electrolyte with high $LiNO_3$ content. *Journal of Power Sources* **2021,** *490*, 229504.
